# Ciliary beating patterns map onto a low-dimensional behavioural space

**DOI:** 10.1038/s41567-021-01446-2

**Published:** 2022-01-10

**Authors:** Veikko F. Geyer, Jonathon Howard, Pablo Sartori

**Affiliations:** 1 B CUBE–Center for Molecular Bioengineering, Technische Universität Dresden, Dresden, Germany.; 2 Department of Molecular Biophysics and Biochemistry, Yale University, New Haven, CT, USA.; 3 Instituto Gulbenkian de Ciência, Oeiras, Portugal.

## Abstract

Biological systems are robust to perturbations at both the genetic and environmental levels, although these same perturbations can elicit variation in behaviour. The interplay between functional robustness and behavioural variability is exemplified at the organellar level by the beating of cilia and flagella. Cilia are motile despite wide genetic diversity between and within species, differences in intracellular concentrations of ATP and calcium, and considerable environment fluctuations in temperature and viscosity. At the same time, these perturbations result in a variety of spatio-temporal patterns that span a rich behavioural space. To investigate this behavioural space we analysed the dynamics of isolated cilia from the unicellular algae *Chlamydomonas reinhardtii* under many different environmental and genetic conditions. We found that, despite large changes in beat frequency and amplitude, the space of waveform shapes is low-dimensional in the sense that two features account for 80% of the observed variation. The geometry of this behavioural space accords with the predictions of a simple mechanochemical model in the low-viscosity regime. This allowed us to associate waveform shape variability with changes in only the curvature response coefficients of the dynein motors.

Biological systems can function despite genetic and environmental perturbations in their molecular components. These perturbations, in turn, affect behaviour at higher levels, from organelles through cells to whole organisms. A key finding is that behavioural spaces, the spaces that embed the whole repertoire of behaviours, tend to be low-dimensional. Examples are the shapes of moving nematodes^1,2^, the swimming trajectories of ciliates and bacteria^3,4^, the postures of walking flies^5^ and mice^6^ and the activities of *Hydra*^7^, which can all be reduced to low-dimensional behavioural spaces. An important conclusion of these analyses is that only a few features define each behaviour, although there can be considerable variation of these features among individuals within that behaviour. A challenge is to map these behavioural spaces onto underlying molecular and biophysical mechanisms^8^.

Cilia are motile organelles that undergo complex shape changes. These changes drive motion relative to the surrounding fluid and allow cilia to respond to external cues. Cilia, therefore, display a simple form of behaviour that makes them a potentially good model system for relating behaviour to underlying molecular mechanisms. A key feature of cilia is that they can beat in the presence of a wide range of genetic mutations of key proteins^9–11^ and can operate over a wide range of temperatures^12^, viscosities^12,13^, ATP concentrations^14^ and buffer conditions such as pH and calcium concentration^15–17^. Thus, cilia exemplify conserved function^18^, namely beating, in the face of large genetic and environmental perturbations. In this work we use variations in beat shapes elicited by these perturbations to construct the behavioural space of cilia.

The beating motion of cilia is powered by thousands of dynein motors belonging to several different classes^19^. These motors drive the sliding of adjacent doublet microtubules^20^ within the axoneme, the structural core of cilia^21^. Sliding is converted to bending by proteins that constrain the shearing of doublets (for example, nexin links or radial spokes)^20,22–24^ and sliding at the base^25^. This biomechanical and structural knowledge forms the basis of mechanochemical models that can successfully recapitulate the oscillatory motion of cilia^26–32^ and synthetic filament bundles^33,34^. The combination of mechanistic models, cited in this paragraph, with phenotypic variability in response to perturbations, cited in the previous paragraph, makes the cilium an ideal model system to study the connection between behavioural spaces and molecular mechanisms.

In this work, we have asked: what is the geometry of the behavioural space of beating cilia subject to a wide range of perturbations, and how do the individual components of this space relate to the underlying mechanochemistry.

## Results

### Quantification of the ciliary beat.

**Fig. 1 |:**
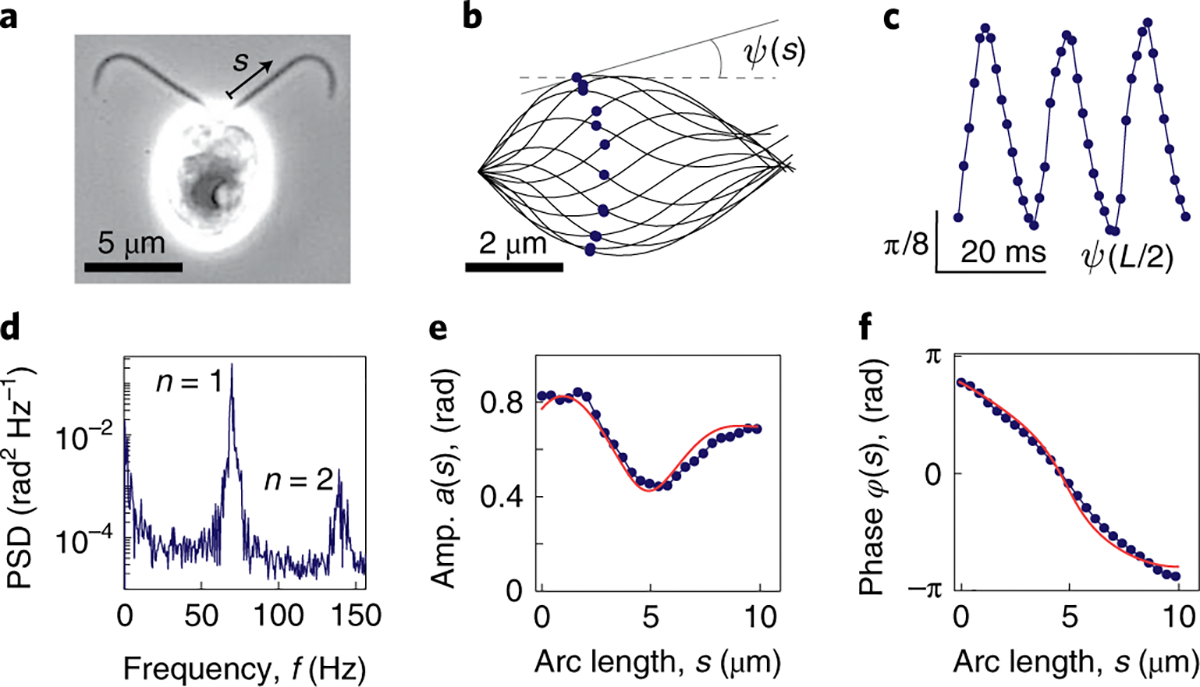
Quantification of ciliary beats. **a**, Wild-type *Chlamydomonas* cell imaged by phase-contrast microscopy. **b**, Tracked waveforms for one beat cycle in the co-swimming frame (1 ms between curves) after the static mode was removed. **c**, Tangent angle at the midpoint as a function of time. **d**, Power spectral density (PSD) of the tangent angle averaged over arc length. **e**, Amplitude (Amp.) of the fundamental mode (*n* = 1) as a function of arc length. The dip in the middle is characteristic of wild-type axonemes. **f**, Phase of the fundamental mode as a function of arc length. The approximately linear decrease in phase indicates steady propagation of the wave from the base (cell body). The red curves in **e** and **f** correspond to the mechanochemical model described in the Methods.

We measured the waveforms of isolated and reactivated *Chlamydomonas reinhardtii* axonemes (Fig. 1a) with high temporal and spatial precision (see ref. ^32^ and Methods). The waveforms, discretized in 25 points along the arc length of the axoneme, were tracked over time for up to 200 beat cycles using filament tracking software^35^ (one beat cycle is shown in Fig. 1b).

The periodic beat of the axoneme was parameterized by the tangent angle ψ(s,t) (with s the arc length and t the time) relative to the co-swimming frame^36^ (Fig. 1b). The power spectrum of the tangent angle (Fig. 1d) showed that typically <10% of the power was in the modes n≥2, which we therefore neglected. After subtracting the static mode n=0, discussed in refs. ^32,37^, we parameterized the beat dynamics as a travelling wave:

(1) $$\psi \left(s,t\right)=a\left(s\right)\cos\left(2\pi ft+\varphi \left(s\right)\right),$$

where a(s) is the amplitude profile, φ(s) is the phase profile and f is the beat frequency. The arc length was normalized by the total length L of the axoneme, so that s∈[0,1]. Although a(s) and φ(s) are often approximated by scalars, such as the mean amplitude $a_{0}\equiv \int_{0}^{1}\;a(s)ds$ and the wavelength $\lambda \equiv -\frac{\pi L}{6}\int_{0}^{1}\;(s-1/2)\varphi (s)ds$, here we are interested in their arc-length dependence, which defines the shape of the beat. Our goal is to characterize variations in the set of parameters 𝒫={a(s),φ(s),f,L}, which we define as the behavioural space of beating. A subset of these parameters, $\left\{a(s)/a_{0},\varphi (s)\right\}$}, defines the shape.

**Fig. 2 |:**
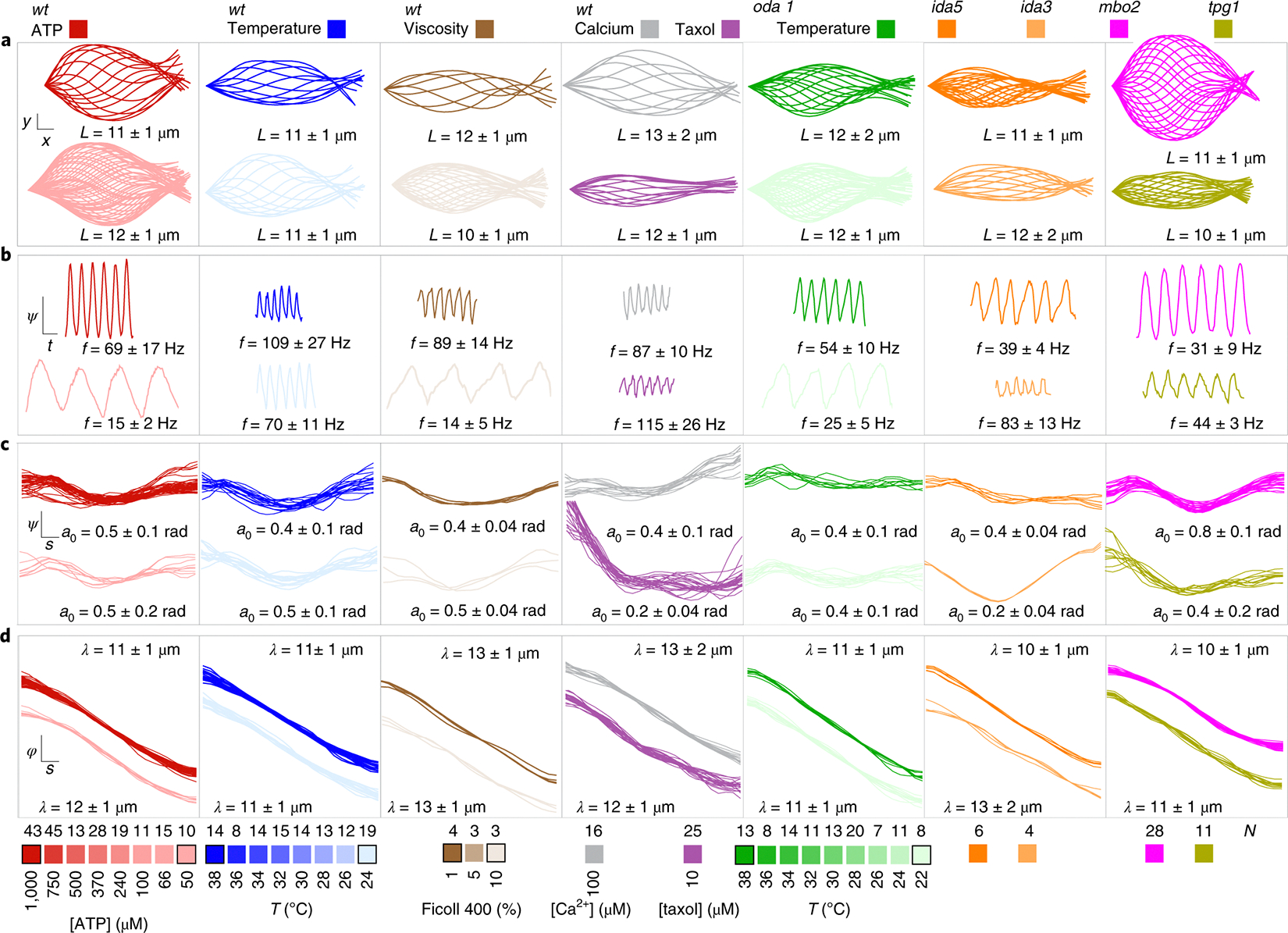
Behavioural variability of the ciliary beat under genetic and environmental perturbations. Each column contains waveform data of a particular cell type and/or environmental condition. Colour legends are given at the bottom. *N* is the total number of distinct axonemes measured for each condition. Data are shown for conditions with black borders. Unless stated otherwise, reactivation was performed under standard conditions (24 °C and 1 mM ATP; Methods). Means and standard deviations of beat parameters are provided in each row. **a**, Sequence of shapes for one beat cycle (1 ms between curves; scale bars, 1 μm). **b**, Tangent angle at the cilium midpoint as a function of time (scale bars, 20 ms and 0.5 rad). **c**, Amplitude profile *a*(*s*) in equation (1) (scale bars, 0.1L and 0.5 rad). **d**, Phase profile φ(s) in equation (1) (scale bars, 0.1L and π/2 rad).

We explored the variability of 𝒫 by observing groups of axonemes under several environmental perturbations (temperature, viscosity, different ATP concentrations, Ca^2+^ and taxol) and genetic perturbations (*oda1*, *ida3*, *ida5*, *mbo2* and *tpg1*) (Fig. 2). Our dataset comprises 498 axonemes undergoing a total of ~40,000 beat cycles. Under all conditions we found beating axonemes, with a percentage of reactivating axonemes greater than 70%. The range of conditions resulted in a wide range of beat shapes and lengths (Fig. 2a), beat frequencies (Fig. 2b), amplitude profiles (Fig. 2c) and phase profiles (Fig. 2d). Therefore, although the beating of cilia is robust against perturbations, the features of its waveform display substantial variations. We now set out to quantify these variations.

### Variations in frequency, mean amplitude and wavelength.

**Fig. 3 |:**
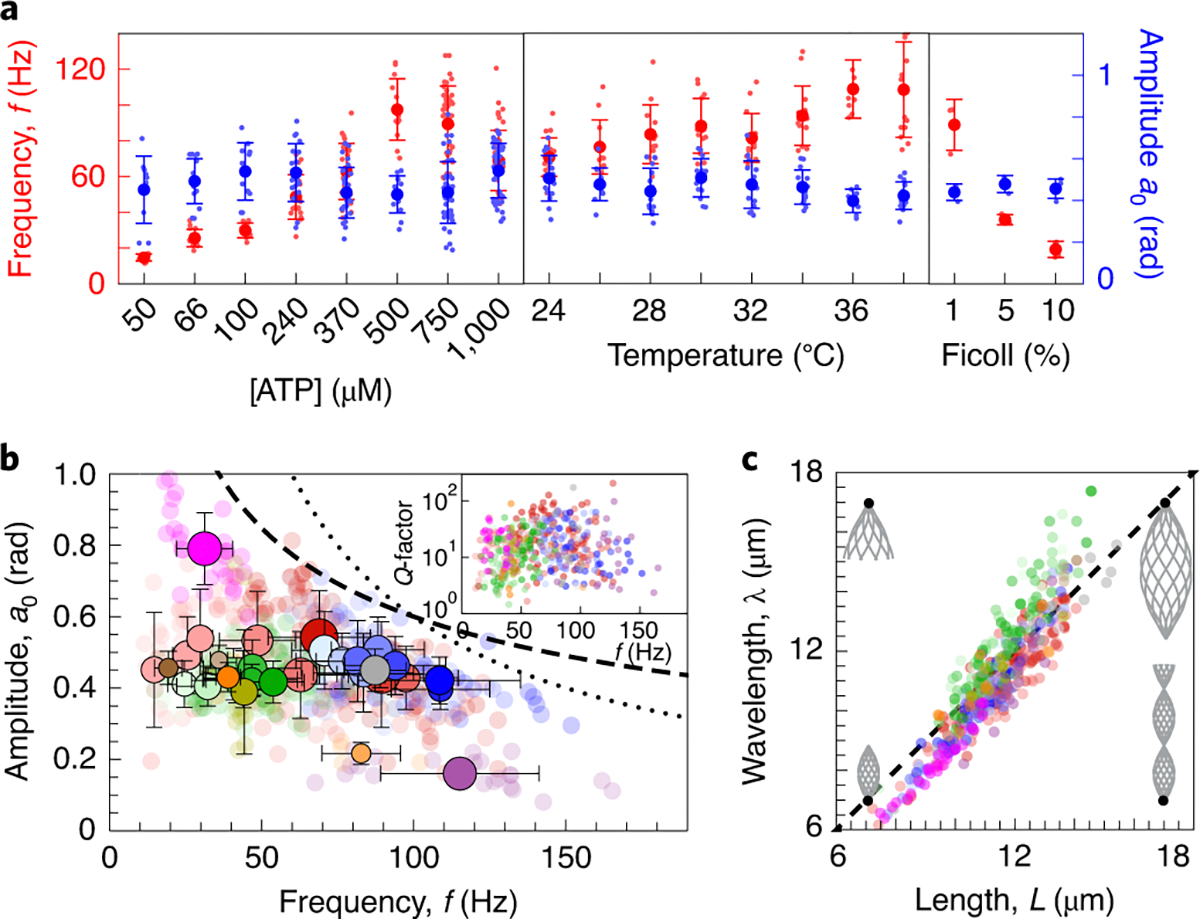
Variations in frequency, mean amplitude and wavelength. **a**, Effect on the mean amplitude and beat frequency of changes in ATP concentration (left), temperature (middle) and viscosity (right). The frequency is strongly dependent on these environmental changes, whereas the amplitude is not. **b**, Scatter plot of amplitude versus frequency. Each transparent circle corresponds to one axoneme, and the solid circle indicates with the radius the size of the dataset. Both the mean amplitude and frequency vary by one order of magnitude. Lack of data with high amplitude and frequency may be due to energetic constraints. Dashed and dotted lines correspond to $1/f^{1/2}$ and 1/f scalings, respectively. Inset: scatter plot of Q-factor. **c**, Scatter plot of wavelength versus length showing that most of the variability in wavelength is correlated with changes in length. Insets: waveforms with lengths/wavelengths corresponding to the black dots at their base.

The beat frequency increases with ATP concentration and temperature and decreases with viscosity (Fig. 3a). This variation encompasses a frequency range of about one order of magnitude, and the between-conditions variance is about seven times larger than the within-condition variance. By contrast, the mean amplitude varies little between these conditions, although it shows large variations within conditions: the between-conditions variance in amplitude is about one-fifth the within-conditions variance. Thus, ATP concentration, temperature and viscosity lead to large changes in frequency with little effect on mean amplitude, in agreement with studies cited in the Introduction.

The mutations *oda1* (which lacks outer-arm dyneins^38^), *ida3* and *ida5* (which lack inner dynein arms f and a, c, d, e, respectively^39,40^), *mbo2* (which lacks microtubule inner proteins and has a symmetric beat^41^) and *tpg1* (which has reduced polyglutamamylation required for axonemal integrity^42,43^), as well as addition of the ion Ca^2+^ and the microtubule poison taxol, did not appreciably broaden the range of beat frequencies (Fig. 3b, 15 Hz to 160 Hz). We note, however, that the *oda1* mutant has a twofold lower beat frequency over all temperatures, as reported in earlier studies (Introduction). The *mbo2* mutation and taxol increased the range of mean amplitudes (Fig. 3b). Interestingly, in *mbo2* the amplitude is high but the beat frequency is low, whereas in taxol (which has not been studied before) the amplitude is low but the beat frequency is high. This reciprocal variation of amplitude and frequency may reflect the energetics of the beat (Discussion).

The variations in the beat frequency and mean amplitude within individual axonemes are small compared to the axoneme-to-axoneme variation. Over the times of analysis, which were typically ~50 cycles, the variances of the frequency and the mean amplitude were considerably smaller than the axoneme-to-axoneme variance (Supplementary Fig. 1 and Supplementary Table 1). It is nonetheless possible that variability within a given condition is due to long-term dynamic effects, for example due to the sampling of axonemes in different states of rundown. Fluctuations in beat frequency over time are so small that the Q-factor of the oscillations can reach values as high as 150 (Fig. 3b, inset), confirming earlier observations^44^. This Q-factor, one of the highest in any biological system, is exceeded by the tuning of sonar responses in the inner ear of the moustached bat (Q up to 1,000 (ref. ^45^)). Thus, the variation in beat frequency and amplitude is not due to short-term variation within individual axonemes, but rather due to differences between axonemes and their response to perturbations.

The lengths of axonemes vary twofold over the entire dataset, from 7.2 μm to 15.4 μm (Fig. 3c). This variation is primarily due to the shorter *mbo2* axonemes and the longer *oda1* axonemes. Remarkably, the wavelengths under all these conditions are almost equal to the lengths, with λ/L=0.97±0.08 (mean ± standard deviation for all 498 cilia) (see analysis relating to Fig. 5).

### Asymmetry and parabolicity are dominant waveform traits.

**Fig. 4 |:**
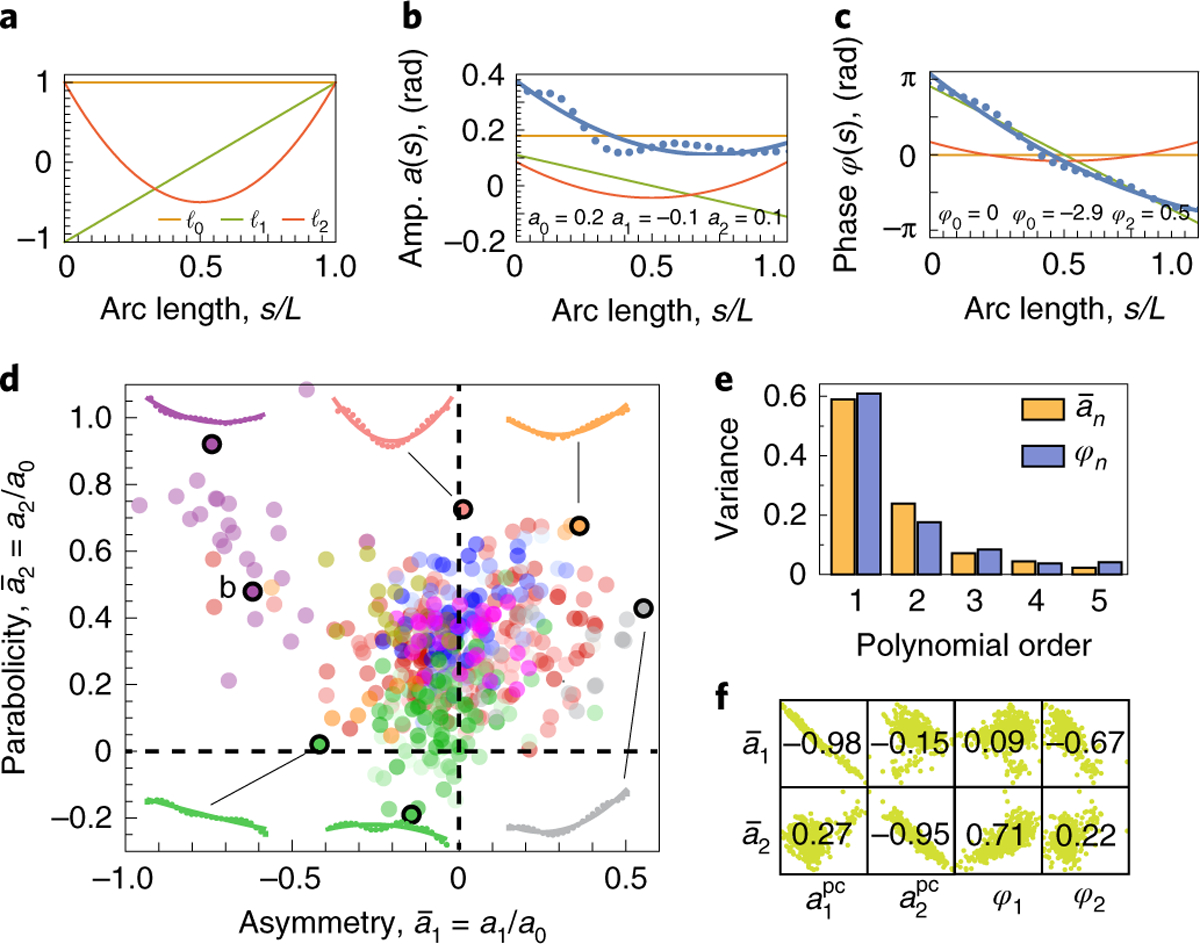
Decomposition of waveform variability. **a**, Shifted Legendre polynomials $\ell_{n}(s)$ for n=0,1,2. The *y*-axis is amplitude. **b**, Decomposition of a particular amplitude profile into the first three shifted Legendre polynomials. $a_{0}$ corresponds to the mean amplitude of Fig. 3, whereas $a_{1}<0$ and $a_{2}>0$ denote decreasing and convex amplitude profiles, respectively. **c**, Same as **b** but for a phase profile. **d**, Scatter plot of the normalized first and second polynomial coefficients for the amplitude. Insets show examples of amplitude profiles for different values of these coefficients. The letter b indicates the data corresponding to the example in **b**. **e**, Fraction of the variance in amplitude and phase profile explained by shifted Legendre polynomials of increasing order. **f**, Table of correlation coefficients of amplitude polynomial coefficients with amplitude principal components and phase polynomial coefficients. Note that ${\overset{‾}{a}}_{1}$ and ${\overset{‾}{a}}_{2}$ are strongly correlated with the first two principal components, $a_{1}^{pc}$ and $a_{2}^{\text{pc}}$, respectively, and are anti-correlated and correlated with $\varphi_{2}$ and $\varphi_{1}$, respectively. The green backgrounds are scatter plots of data.

To characterize the variation in the amplitude and phase profiles of the tangent angle, we decomposed both into shifted Legendre polynomials of increasing order (Methods and Fig. 4a–c). For the amplitude, the lowest order ($a_{0}$) equals the mean amplitude, the first order ($a_{1}$) corresponds to the linear deviation from constant amplitude and is a measure of the proximal–distal asymmetry, and the second order ($a_{2}$) measures the quadratic deviation. For the phase, we set $\varphi_{0}$ to zero (as it is arbitrary), the first order is $\varphi_{1}=-\pi L/\lambda$ by definition of λ (the linear component of the phase profile), and $\varphi_{2}$ corresponds to the quadratic deviation from the line. Because we are interested in shape, we normalized the amplitude coefficients: ${\overset{‾}{a}}_{1}=a_{1}/a_{0}$ and ${\overset{‾}{a}}_{2}=a_{2}/a_{0}$.

Figure 4d displays the space of variations of parabolicity and asymmetry of the amplitude. Most points cluster around ${\overset{‾}{a}}_{1}\approx 0.0$ and ${\overset{‾}{a}}_{2}\approx 0.3$, which corresponds to a symmetric and convex amplitude profile. The exceptions include taxol, *ida5* and *tpg1*, which have ${\overset{‾}{a}}_{1}<0$ corresponding to decreasing amplitudes towards the distal tip, and Ca^2+^, which has ${\overset{‾}{a}}_{1}>0$ corresponding to increasing amplitude towards the distal tip. The *oda1* waveforms have a small parabolicity ${\overset{‾}{a}}_{2}$ corresponding to their linear amplitude profiles. The *ida5* waveforms have smaller parabolicity and negative asymmetry. Thus, despite the robustness of the beat, both genetic and environmental perturbations led to variations in the waveforms.

Parabolicity and asymmetry, the polynomial terms that together contribute 80% of the variance (Fig. 4e), are also the main features of the data: they strongly correlate with the first two principal components $a_{1}^{pc}$ and $a_{2}^{pc}$ (Fig. 4f, left boxes). Furthermore, the asymmetry of the amplitude profile, ${\overset{‾}{a}}_{1}$, is anti-correlated with the parabolicity of the phase profile, $\varphi_{2}$, whereas the parabolicity of the amplitude profile, ${\overset{‾}{a}}_{2}$, is correlated with the asymmetry of the phase profile, $\varphi_{1}$ (Fig. 4f, right boxes). Thus, the dominant shape features are the parabolicity and asymmetry of the amplitude and phase profiles.

### Motor response controls waveform variability.

**Fig. 5 |:**
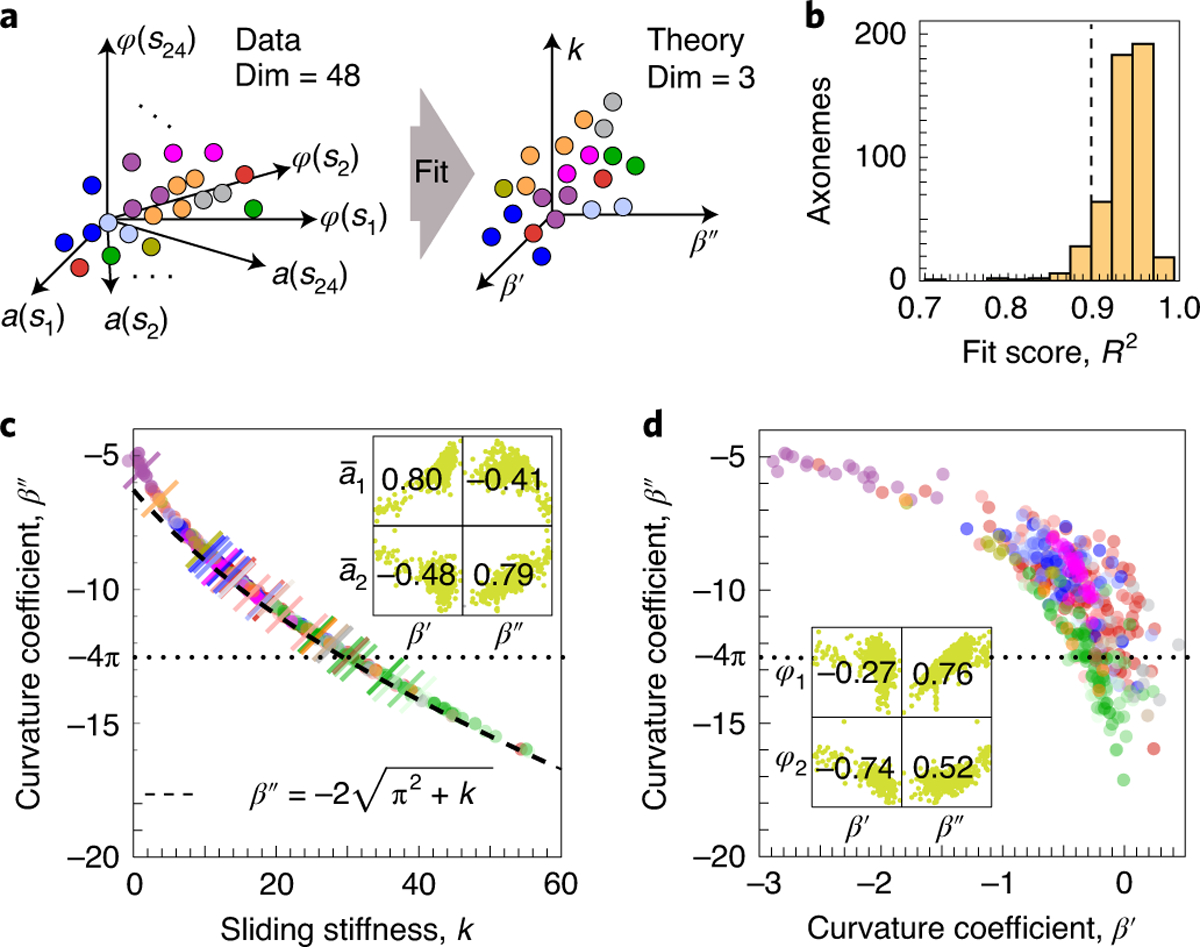
Mapping waveform space to mechanical space. **a**, Experimental waveforms have 48 dimensions (Dim), 24 for the amplitude and 24 for the phase profile. Fitting to a mechanical model, we map these data to a three-dimensional mechanical space, with axes the two response coefficients of the motors and the shear stiffness. **b**, Histogram of the $R^{2}$ of the fits. More than 90% of the fits have $R^{2}>0.9$. **c**, Variability in shear stiffness k and motor response $\beta^{\prime \prime }$. The values of k and $\beta^{\prime \prime }$ are tightly correlated, which corresponds to the low-viscosity limit (dashed line). The dotted line corresponds to -4π and the crossed colored lines correspond to the means of each dataset. The inset shows the correlation between curvature response coefficients and phase asymmetry and parabolicity. **d**, Variability in curvature response coefficients captures waveform variability, with low negative $\beta^{\prime }$ corresponding to low negative ${\overset{‾}{a}}_{1}$ and thus decaying waveforms. The dotted line corresponds to -4π. The inset shows the correlation between the curvature response coefficients and the asymmetry and parabolicity of the phase.

The low-dimensionality of shapes suggests that the perturbations, irrespective of their molecular mechanisms, affect only a few collective properties of the axoneme. To explore this possibility, we asked whether a mechanical model of the ciliary beat, based on that in ref. ^32^, could account for the diversity of waveforms. The model, described in the Methods, contains four non-dimensional coefficients: $\beta^{\prime }$ and $\beta^{\prime \prime }$, which characterize the motors’ dependence on the instantaneous curvature (instantaneous response) and the rate of change of curvature (dynamic response), respectively; k, which is the shear stiffness of the axoneme; and $\bar{Ma}$, which is a constant that comes from non-dimensionalizing Machin’s equation (see equation (4) in ref. ^26^). $\bar{Ma}=2\pi f\xi_{n}L^{4}/\kappa$, where κ is the flexural rigidity of the axoneme and $\xi_{n}$ is the normal friction coefficient. For *Chlamydomonas*, $\bar{Ma}\;\approx \;50$, using the parameters in the Methods. We fitted the mechanical model to the data (see example in Fig. 1e,f). The curve-fitting reduced the dimensionality of the waveform description from 48, $a\left(s_{i}\right)$ and $\varphi \left(s_{i}\right)$ with i=1,…,24 points along the length, to just 3, the model parameters (Fig. 5a). The agreement of the model with the data was excellent, with 92% of the axonemes having $R^{2}>0.9$ (Fig. 5b).

Figure 5c shows that one of the parameters, the sliding stiffness k, is strongly correlated with another parameter, the dynamic motor response $\beta^{\prime \prime }$. The observed relationship between these parameters can be recovered analytically by noting that the ratio of friction to bending forces, which is proportional to the Machin number $Ma\equiv \bar{Ma}/(2\pi {)}^{4}$, is ≪1. Taking the limit Ma→0 gives the curved dashed line in Fig. 5c (Methods). The good match between this ‘dry friction’ limit and the full theory supports the hypothesis that *Chlamydomonas* operate in a low-friction regime (Discussion). Therefore, the waveform is controlled by just the two motor response coefficients, which correlate with the asymmetry and parabolicity of the amplitude (Fig. 5c, inset).

In agreement with the above argument, Fig. 5d recapitulates the variability in waveform features observed in Fig. 4d. For example, the taxol and Ca^2+^ data lie far apart from each other in the ($\beta^{\prime },\beta^{\prime \prime }$) space, just as in the (${\overset{‾}{a}}_{1},{\overset{‾}{a}}_{2}$) space. Furthermore, because amplitude and phase are correlated, the motor response coefficients also correlate with the phase features (Fig. 5d, inset). Parameters $\beta^{\prime }$ and $\beta^{\prime \prime }$ not only control the asymmetry and parabolicity of the amplitude, respectively, but $\beta^{\prime \prime }$ also controls the wavelength. Specifically, in the limit Ma→0, we can show that the curvature wavelength is $\lambda =-4\pi L/\beta^{\prime \prime }$, and therefore λ/L=1, which is typically observed, requires $\beta^{\prime \prime }=-4\pi$, in agreement with the average values in Fig. 5c (dotted line) and Fig. 5d. Thus, the space spanned by the motor response coefficients accurately recapitulates the space of amplitude and phase features, which suggests that all genetic and environmental perturbations are buffered into changes of the curvature regulation of the motors.

## Discussion

### The dimensionality of the ciliary beat is low.

By using a wide variety of environmental and genetic perturbations, we have constructed the behavioural space of ciliary swimming waveforms. Asymmetry (${\overset{‾}{a}}_{1}$ and $\varphi_{1}$) and parabolicity (${\overset{‾}{a}}_{2}$ and $\varphi_{2}$) of the amplitude and phase profiles suffice to describe about 80% of the variation between and within conditions. The dimensionality of swimming behaviour for isolated axonemes is therefore very low: beat frequency, mean amplitude, shape asymmetry, shape parabolicity and axoneme length. Low-dimensional phenotypic spaces have been observed in other behaviours (Introduction), but the range of conditions probed in our study makes the low-dimensionality even more remarkable. It remains open how the dimensionality of isolated swimming axonemes relates to that of intact cells.

### The shape space is spanned by the curvature sensitivity of the dyneins.

The geometry of the shape space (the amplitude and phase profiles) accords with a simple biomechanical model of ciliary beat, which is an extension of a previous model^32^. In this model, dynein motors respond to curvature with an instantaneous coefficient $\beta^{\prime }$ and a dynamic coefficient $\beta^{\prime \prime }$. The model also includes a passive stiffness to the sliding of doublets, k. Note that the typical value k=20 (non-dimensionalized from Fig. 5c), which corresponds to 120 pN, is in good agreement with the measured value of 80 pN from ref. ^46^.

The connection between the shape and biomechanical spaces enabled a remarkable finding: the curvature coefficients $\beta^{\prime }$ and $\beta^{\prime \prime }$ strongly correlate with the shape parameters, ${\overset{‾}{a}}_{1},{\overset{‾}{a}}_{2},\varphi_{1}$ and $\varphi_{2}$:

(2) $${\overset{‾}{a}}_{1}∼\beta^{\prime }∼-\varphi_{2}\;\text{and}\;{\overset{‾}{a}}_{2}∼\beta^{\prime \prime }∼+\varphi_{1}\propto \lambda /L,$$

where ‘~’ denotes correlation. Thus, the different shapes correspond to different curvature sensitivities.

Wild-type cilia have symmetric and convex amplitude profiles, ${\overset{‾}{a}}_{1}\approx 0.0$ and ${\overset{‾}{a}}_{2}\approx 0.3$, corresponding to $\beta^{\prime }=0$ and $\beta^{\prime \prime }\approx -10$. Using equation (2), we can ascribe deviations from this typical behaviour to changes in motor properties. For example, taxol, *tpg1* and *ida5* have ${\overset{‾}{a}}_{1}<0$, corresponding to a beat whose amplitude decays distally. Despite the different nature of these perturbations, we find that all three perturbations decrease the instantaneous response coefficient, $\beta^{\prime }$, and increase the dynamic response coefficient, $\beta^{\prime \prime }$. This means that the sensitivity to instantaneous curvature is increased, whereas the sensitivity to dynamic curvature is decreased. In other words, the phase of the curvature response is altered.

Conversely, Ca^2+^ leads to ${\overset{‾}{a}}_{1}>0$, and so $\beta^{\prime }>0$. The *oda1* mutation has a flat profile, with ${\overset{‾}{a}}_{2}\approx 0$ and ${\overset{‾}{a}}_{1}\approx 0$, and so $\beta^{\prime }\approx 0$ and $\beta^{\prime \prime }\approx -12$. This counters the general belief that outer-arm dyneins affect only beat frequency^47^. The *ida3* mutation has a high parabolicity, that is, a larger dip in the middle, which reflects a less negative value of the dynamic curvature coefficient. The *mbo2* mutation has a waveform very similar to that of wild type, which is remarkable given the absence of a static mode in the waveform of this mutant^37,41^ but is consistent with the observation that the *mbo2* mutations do not affect dyneins^47^. Thus, changes in the instantaneous and dynamic curvature sensitivity of the dyneins can account for the effects of environmental and genetic perturbations, although a causal connection between the perturbations and dynein activity has not been proven. Studying the relationship between shape and model parameters for alternative motor mechanisms (for example, ‘geometric clutch’^28^ or compression instability^29,48^) may give further insight in this direction.

### Elasticity dominates *Chlamydomonas* swimming.

Our results contain three arguments that viscous forces are smaller than elastic forces for *Chlamydomonas* axonemes. First, the Machin number, which is the ratio of viscous to elastic forces and is related to the Weissenberg number^49^, is much smaller than unity (Ma≪1). For a plane wave with wavenumber q=2π/λ, we have

(3) $$Ma=\frac{\xi_{n}\omega }{\kappa q^{4}}=\frac{1}{(2\pi {)}^{4}}\bar{Ma},$$

which ranges from 0.02 to 0.14 under all conditions. Note, however, that there is considerable uncertainty in κ, especially in the presence of taxol^50^ (even if the flexural rigidity of the axoneme were reduced 10-fold by taxol, our models shows that there would be only a small effect on the shape). Second, the data collapse of the fitted k and $\beta^{\prime \prime }$ in Fig. 5c almost coincides with that predicted by the low-viscosity relation (dashed line). This extends our earlier finding that a low Ma accords with wild-type and *mbo2* axonemes^32^. Third, despite the wide variability in frequency and mean amplitude, the total range is ~10-fold for both, there is an absence of high-amplitude and high-frequency beats. The dissipation of elastic energy is proportional to $a_{0}^{2}f$ whereas the dissipation of viscous energy is proportional to $a_{0}^{2}f^{2}$ (ref. ^26^). Thus, if elastic or viscous energy were limited by the energy available from the hydrolysis of ATP by the motors, then $a_{0}∼f^{-1/2}$ or $a_{0}∼f^{-1}$, respectively. The former provides a better bound on the data than the latter (Fig. 3b dashed and dotted curves, respectively), as expected when elastic dissipation dominates.

A puzzling finding is that the Machin number measured experimentally is relatively insensitive to changing viscosity because increasing viscosity decreases the beat frequency, compensating for much of the expected change in Ma. This highlights our lack of understanding of how the beat frequency is selected. The low-viscosity regime is also supported by the literature. In ref. ^33^, the ATPase rate of axonemes was shown to increase in proportion to beat frequency, as predicted for elastic dissipation, and in ref. ^51^ measurements of the flow field around *Chlamydomonas* cilia showed that friction forces are smaller than the estimated bending forces. Furthermore, a low Ma may also explain the different relationship between curvature and sliding force reported in ref. ^52^ relative to that of our model^32^, as the larger Ma of sperm may allow for a different motor control mechanism^31^. We conclude that *Chlamydomonas* swims in the regime of low Ma, that is, low friction. Because the Reynolds number Re, which is the ratio of inertial to viscous forces, is also small, we have Re≪Ma≪1.

### Perspective.

The functional robustness of the ciliary beat, evidenced by its persistence in the face of genetic and environmental perturbations, indicates that the ciliary beat is a highly canalized process^53^. Such processes can tolerate genetic variations, pre-adaptations, which can be selected for if large environmental changes occur^54–56^. This may underlie the diversity of axonemal forms. For example, the number of doublet microtubules in motile axonemes ranges from as few as three^57^ to as many as hundreds^58^. Furthermore, radial spokes and the central pair are missing in some motile cilia, and additional asymmetrically localized molecules lead to planar or asymmetric beat patterns^59^. Axonemes can be long or short, can be solitary or arrayed and can drive cell motility or fluid flow. This diversity of axonemal structure and function likely originates, at least in part, in the strong tendency of motor-driven bending of cytoskeletal filaments to produce oscillations when the bending feeds back on the activity of the motors, as we postulate for dynein. The propensity for oscillation likely underlies the successful reconstitution of cilia-like motility using heterologous motors and filaments^33,34^.

## Online content

Any methods, additional references, Nature Research reporting summaries, source data, extended data, supplementary information, acknowledgements, peer review information; details of author contributions and competing interests; and statements of data and code availability are available at https://doi.org/10.1038/s41567-021-01446-2.

## Methods

### Experiments.

#### Preparation and reactivation of axonemes.

Axonemes from *Chlamydomonas reinhardtii* cells (received from http://chlamy.org) were purified and reactivated. The procedures described here are detailed in ref. ^60^.

Chemicals were purchased from Sigma Aldrich unless stated otherwise. In brief, cells were grown in tris–acetate–phosphate medium with additional phosphate (TAP+P medium) under conditions of continuous illumination (from two 75 W fluorescent bulbs) and of air bubbling at 24 °C over the course of 2 days, to a final density of 10^6^ cells per millilitre. Cilia were isolated using dibucaine, then purified on a 25% sucrose cushion and demembranated in HMDEK buffer (30 mM HEPES-KOH, 5 mM MgSO_4_, 1 mM dithiothreitol, 1 mM EGTA and 50 mM potassium acetate, at pH 7.4) augmented with 1% (v/v) IGEPAL detergent and 0.2 mM Pefabloc SC protease inhibitor. The membrane-free axonemes were resuspended in HMDEK plus 1% (w/v) polyethylene glycol (molecular weight 20 kDa), 30% sucrose and 0.2 mM Pefabloc, and were stored at −80 °C. Prior to reactivation, axonemes were thawed at room temperature, then kept on ice. Thawed axonemes were used for up to 2 h.

Reactivation was performed in flow chambers with a depth of 100 μm built from easy-clean glass and double-sided sticky tape. Thawed axonemes were diluted in HMDEKP (HMDEK augmented with 1% (w/v) PEG 20.000) reactivation buffer. Unless stated otherwise, a standard reactivation buffer containing 1 mM ATP and an ATP-regeneration system (5 units per millilitre creatine kinase and 6 mM creatine phosphate) was used to maintain a constant ATP concentration. The axoneme dilution was infused into a glass chamber, which was blocked using casein solution (from bovine milk, 2 mg mL^−1^) for 10 min and then sealed with vacuum grease. Prior to imaging, the sample was equilibrated on the microscope for 5 min and data were collected for a maximum time of 20 min.

#### Special reactivation conditions.

For the temperature series, the temperature was controlled using an objective heater from Bioptech. Unless stated otherwise, the sample temperature was kept constant at 24 °C. The temperature series was acquired by increasing the temperature in 2 °C steps and letting the system equilibrate for 10 min. After equilibration, the target temperature was checked using an inbuilt reference thermistor. For the ATP series, the standard buffer (without ATP) was augmented with different amounts of ATP (50, 66, 100, 240, 370, 500, 750 and 1,000 μM). For the viscosity series, the standard buffer was augmented with Ficoll 400 (1%, 5% 10% (w/v)), and then axonemes were added to this solution. For the calcium, we used a Ca^2+^ buffered reactivation solution with a concentration of free Ca^2+^ of 100 μM (calculated with the program Maxchelator https://somapp.ucdmc.ucdavis.edu/pharmacology/bers/maxchelator). This concentration was chosen as it converts the asymmetric beat into a symmetric one^61^. For the taxol, the standard buffer was augmented with 10 μM taxol, a concentration that stabilizes polymerized microtubules^62^, and then axonemes were added to this solution.

#### Imaging of axonemes.

The reactivated axonemes were imaged by phase-contrast microscopy, set up on an inverted Zeiss Axiovert S100 TV or Zeiss Observer Z1 microscope using a Zeiss 63× Plan-Apochromat NA 1.4 or a 40× Plan-Neofluar NA 1.3 Phase 3 oil lens in combination with a 1.6× tube lens and a Zeiss oil condenser (NA 1.4). Movies were acquired using an EoSens 3CL CMOS high-speed camera. The effective pixel size was 139 nm or 219 nm per pixel. Movies of up to 3,000 frames were recorded at a frame rate of 1,000 fps.

### Data analysis.

#### High-precision tracking of isolated axonemes.

To track the shape of the axoneme in each movie frame with nanometre precision, the MATLAB-based software tool FIESTA Version 1.03 was used^35^. Prior to tracking, movies were background-subtracted to remove static inhomogeneities arising from uneven illumination and from dirt particles. The background image contained the mean intensity in each pixel calculated over the entire movie. This procedure increased the signal-to-noise ratio by a factor of three^60^. Phase-contrast images were inverted. For tracking, a segment size of 733 nm (approximately 5 × 5 pixels) was used, corresponding to program settings of a full width at half maximum of 750 nm and of a ‘reduced box size for tracking especially curved filaments’ of 30%. Along the arc length of each filament, 25 equally spaced segments were fitted using two-dimensional Gaussian functions.

#### Fourier analysis of tangent angle.

Using the x, y positions of the filament shape we calculated the tangent angle ψ(s,t) at every arc-length position in time. To study only the dynamic modes of the waveform we subtracted the static mode (the 0th Fourier mode)^37^ and Fourier-decomposed the tangent angle. The spectrum of dynamic modes of the tangent angle shows a peak at the fundamental frequency (Fig. 1d, first Fourier mode). Because the fundamental mode accounts for >90% of the total power, we neglected the higher harmonics (n=2,3,4,…) in all further analysis (see ref. ^32^ for more detail) and considered only the first Fourier mode of ψ(s,t), which is denoted as ψ(s) and will be used hereafter for all dynamic quantities. We calculated the frequency f=⟨f(s)⟩ and the arc-length-dependent amplitude a(s)=∣ψ(s)∣ and phase φ(s)=argψ(s) profiles of the fundamental Fourier mode.

#### Polynomial decomposition.

We use shifted Legendre polynomials, $\ell_{n}(s)$, with normalization $\int_{0}^{1}\;\ell_{n}(s)\ell_{m}(s)ds=\frac{1}{2n+1}\delta_{mn}$, to characterize amplitude and phase profiles. Any function g(s) with support [0,1] can be written as $g(s)=\sum_{n\geq 0}\;g_{n}\ell_{n}(s)$, with $g_{n}=(2n+1)\int_{0}^{1}\;g(s)\ell_{n}(s)ds$. With this definition, $g_{0}=\int_{0}^{1}\;g(s)ds$ is the mean over the arc length.

### Theory.

#### Mechanical model of the axoneme.

The dynamics of the axoneme is characterized by a balance of hydrodynamic, elastic and internal sliding forces^26,63^. The sliding forces can be active, exerted by motors, or passive, associated with structures such as nexin links and radial spokes in addition to the motors. To linear order and in frequency space, the force balance equation, which we call the Machin equation, is given by

(4) $$i\bar{Ma}\psi =-\partial_{s}^{4}\psi +\partial_{s}^{2}f_{sl},$$

where $f_{sl}$ is the dimensionless sliding force density and $\bar{Ma}=2\pi f\xi_{n}L^{4}/\kappa$ is a dimensionless constant related to the Machin number by $\bar{Ma}=(2\pi {)}^{4}Ma$. A global balance of sliding forces also holds, $\int_{0}^{1}\;f_{sl}(s)ds=F_{b}$, where the basal force is given by $F_{b}=\chi_{b}\Delta_{b}$ with $\Delta_{b}$ the basal sliding and $\chi_{b}=\chi_{b}^{\prime }+i\chi_{b}^{\prime \prime }$ the complex basal response coefficient with $\chi_{b}^{\prime },\chi_{b}^{\prime \prime }>0$.

We take the sliding force density $f_{sl}(s)$ generated by motors to depend on the local curvature $\partial_{s}\psi (s)$ and sliding $\Delta (s)=\Delta_{b}+\psi (s)-\psi (0)$ of doublets^32^. In particular,

(5) $$f_{sl}=k\Delta +\beta \partial_{s}\psi ,$$

where k>0 is a passive sliding stiffness and $\beta =\beta^{\prime }+i\beta^{\prime \prime }$ is a complex curvature response coefficient. For $\beta^{\prime \prime }<0$ there is forward (base-to-tip) wave propagation^64^. Note that, unlike in ref. ^64^, we allow for instantaneous curvature response via $\beta^{\prime }$, which is key for characterizing amplitude asymmetry (in ref. ^32^, $\beta^{\prime }=0$ was sufficient because only the symmetric wild-type and *mbo2* axonemes were studied). The model does not speak to the mechanism of curvature sensing, which must be very sensitive given that axonemal curvatures are low. Such curvature sensitivity might be realized by strains that distort the cross-section of the axoneme, for example due to the ‘geometric clutch’ model^28^ or to the coupling between twist and bending^64^. As boundary conditions we use torque and force balances: $\partial_{s}\psi (0)=F_{b},\partial_{s}\psi (1)=0$, $\partial_{s}^{2}\psi (0)=f_{sl}(0)$, and $\partial_{s}^{2}\psi (1)=f_{sl}(1)$. Equations (4) and (5) correspond to the solution of a non-linear problem near the point of instability^63^. Note that we have not considered the role of three-dimensional components to the beat, for example via twist^64^.

#### Model fitting.

In previous works, we have used the Machin equation and its variants to reproduce the observed beating patterns of cilia^31,64^. For a given value of $\bar{Ma}$, estimated from experiments, and values for the sliding response coefficients $k,\beta^{\prime }$ and $\beta^{\prime \prime }$, there exists a discrete set of solutions to the system of equations posed by equations (4) and (5), the global sliding balance and the boundary conditions^36,63^. We keep the solution with the lowest wavenumber, because for simple dynamical non-linear motor models it has been shown to be the first one to be excited (see Section 5.2.2 in ref. ^65^), so that for a given set of motor response parameters we can determine a unique theoretical waveform, $\left\{k,\beta^{\prime },\beta^{\prime \prime }\right\}\to \psi_{\text{the}}(s)$.

The procedure to fit the model to the data has been extensively described in ref. ^36^. In brief, we define a score between the theoretically predicted waveform and the experimentally observed one, $R^{2}\left(\psi_{\text{the}},\psi_{\text{exp}}\right)$, so that $R^{2}=1$ corresponds to a perfect matching of theory and experiments^36,64^. We then maximize this score with respect to the three parameters $\left\{k,\beta^{\prime },\beta^{\prime \prime }\right\}$ using a principal axis algorithm with three different initial points: $\left\{3\pi^{2},0,-4\pi \right\}$, which corresponds to the plane wave approximation, and two perturbations around it, {1.7,-2.1,-5.8} and $\left\{3\pi^{2},0.15,-9.6\right\}$. In addition, to calculate $\bar{Ma}$, we used $\kappa =580\;pN\;\mu m^{2}$ and $\xi_{n}=0.0049\;pN\;s\;\mu m^{-2}$ (ref. 64). There is a considerable range of values of these parameters in the literature. For example, in ref. ^66^ the normal friction coefficient was estimated to be $\xi_{n}=0.0015\;pN\;s\;\mu^{-2}$ based on measurements in intact cells, and in ref. ^46^ a bending modulus of $\kappa =800\;pN\;\mu m^{2}$ was obtained using optical tweezers. Because we are in the low-Machin-number regime, our analysis is quite insensitive to changes in these two parameters, which was confirmed by increasing and decreasing the Machin number 10-fold. In the Ficoll datasets we adjusted $\xi_{n}$ following our own measurements by factors of 1.1, 1.6 and 3.2 for dilutions of 1%, 5% and 10%, respectively.

#### Low friction limit.

Although equations (4) and (5) are linear, the boundary conditions result in complicated relationships between the exponential scales in the solution and the motor parameters^36^. We found that such complications are greatly reduced in the limit $\bar{Ma}\to 0$. It is particularly convenient to work in terms of curvature $\partial_{s}\psi$, which can be shown to take the form

(6) $$\partial_{s}\psi =e^{cs}\left(e^{cd(s-1)}-e^{-cd(s-1)}\right),$$

with c=β/2 and $d=\sqrt{1+4k/\beta^{2}}$ where the sliding stiffness, k, is now allowed to be complex, with the imaginary component corresponding to sliding friction. The general condition for the existence of a solution in this limit is simply

(7) $$\tanh\left(cd\right)=-\frac{\chi_{b}cd}{\chi_{b}c+k},$$

As the observed values for the basal stiffness arising from the fits are small, we explore the limit $\chi_{b}\to 0$. In this limit equation (7) becomes tanh(cd)=0, which implies cd=inπ, with n=1,2,… From this we obtain

(8) $$\partial_{s}\psi =exp\left(\beta^{\prime }s/2\right)\;sin\;(n\pi (s-1))\;exp\;\left(i\beta^{\prime \prime }s/2\right),$$

with the sliding stiffness and curvature response coefficients related by $\beta \sqrt{1+4k/\beta^{2}}=i2\pi n$. Note that, in the case that k is real, we have $\beta^{\prime }=0$. The dashed line in Fig. 5 corresponds to the previous equation when n=1, which provides good agreement with the fits for the full model (finite viscosity, non-zero basal compliance and non-zero asymmetry). Note that in equation (8) the amplitude asymmetry is directly related to $\beta^{\prime }$, with $\beta^{\prime }<0$ corresponding to decreasing amplitude. Furthermore, the wavelength can be directly read off from this expression, and is given by $\lambda =-4\pi L/\beta^{\prime \prime }$. This is also in agreement with observations from fitting the full model.

## Supplementary Material

Supplimentary Data

Supplimentary Info

Additional information

**Supplementary information** The online version contains supplementary material available at https://doi.org/10.1038/s41567-021-01446-2.

## Data Availability

The data shown in the main figures are appended to this article in csv format. In addition, all tracked waveforms are available on Dryad (https://doi.org/10.5061/dryad.0gb5mkm2j).
