# Publisher Correction: Ciliary beating patterns map onto a low-dimensional behavioural space

**DOI:** 10.1038/s41567-022-01552-9

**Published:** 2022-02-23

**Authors:** Veikko F. Geyer, Jonathon Howard, Pablo Sartori

Correction to: *Nature Physics* https://doi.org/10.1038/s41567-021-01446-2, published online 10 January 2022.

In the version of this article initially published, in Fig. 2d, the boxes above ‘Ficoll 400 (%)’ should have been labelled 1, 5, and 10 instead of 10, 5 and 1.

In the ‘Data availability’ section, the URL for Dryad should have been https://doi.org/10.5061/dryad.0gb5mkm2j.

In Equation 4, a minus sign preceding the right-hand term was missing, while in the fourth line of the description of Equation 4, subscript b’s were missing. The term should be “χ′_b_ + χ″_b_”.

The errors have been corrected in the HTML and PDF versions of the article.

