# Supplementary material for: Ciliary beating patterns map onto a low-dimensional behavioural space
Source: Nat Phys. Author manuscript; Available in PMC 2025 Jul 10. (PMC12242449; doi:10.1038/s41567-021-01446-2)
Supplement: Supplimentary Data [file NIHMS2088797-supplement-Supplimentary_Data.pdf]

Labels	Length	Frequency	Amplitude	PcAmplitude1	PcAmplitude2	PcPhase1	PcPhase2	LegAmplitude1	LegAmplitude2	LegPhase1	LegPhase2	FitScore	k	Beta'	Beta"
ATP_50_1	11.66	16.94	0.5465614951470660	0.14735505471375400	0.5063822336321360	0.16330110830886200	0.6133340980385780	-0.14941063142703800	0.15321099181112700	-3.1560044016213500	-0.07535873640503610	98.23493106415370	31.428273361534100	-0.14083977863216900	-12.703050340913600
ATP_50_2	13.04	16.34	0.3404167900312260	0.22133371554576000	0.07364548581832790	-0.0456975848301622	0.08912440282501810	-0.11701116213800800	0.3164073221697880	-3.06780917532675	0.10619863336767400	97.69381050392050	19.904081446241000	-0.38398293107271300	-10.597312484229000
ATP_50_3	13.34	13.65	0.19348447931671200	0.42565877445307900	0.15887653396225000	-0.18136160154553300	0.6245217164799860	-0.15989934529480100	0.228928642448641	-3.0531082089695500	-0.03977920601612460	96.52879724370740	24.63480784448660	-0.12366313754088900	-11.589516288602500
ATP_50_4	13.34	13.65	0.19348447931671200	0.4256587744530780	0.15887653396224800	-0.18136160154553200	0.6245217164799860	-0.15989934529480100	0.228928642448641	-3.0531082089695500	-0.03977920601612460	96.52879724370740	24.63480784448660	-0.12366313754088900	-11.589516288602500
ATP_50_5	11.18	15.64	0.48145893778756400	-0.7201961139478790	-0.17554208615154700	-0.31036509665667900	0.33663226272123400	0.20382404290953400	0.34609882400016400	-2.9759622219144400	0.0016958115862526800	95.88586982716770	21.63291059893090	-0.2702701764069360	-11.106361238610600
ATP_50_6	11.14	16.32	0.5074777692662040	-0.6810078609594320	-0.088422410698559	-0.23617905691316400	0.3919722764783920	0.18862196219445600	0.3076324630069080	-3.0084984844710500	-0.007896030031264570	96.63319986945030	23.44123079298550	-0.24759680363315700	-11.43328755337010
ATP_50_7	11.23	16.1	0.5572695773197610	0.04919118966155760	0.33946045781438900	-0.35004273772931200	0.4126017794919630	-0.09699869502977050	0.20721395310108500	-2.9799180477894700	-0.004243549201264910	97.43739824437210	23.711219895835900	-0.20390154816473300	-11.429455075386100
ATP_50_8	12.14	14.43	0.6955750302542540	-0.20411372880157500	0.3828150704169070	0.0385743532142988	0.6783341777621310	-0.022050173779235800	0.17080258299539900	-3.0722145207207100	-0.18985498800273200	95.40444009000510	29.093995592251600	-0.15505627242444100	-12.379538938153300
ATP_50_9	11.58	11.44	0.5229776518425660	-0.459825653816002	0.2435410865901110	0.15909673657952800	0.6928751134236840	0.08804514005726690	0.18477056850934600	-3.119232164071680	-0.1856352756125800	95.60456774456380	32.40670324672790	-0.1603763112362500	-12.922091207545700
ATP_50_10	11.9	12	0.4591745523368310	-0.08701505877579350	0.4455531514863070	0.6595058399206670	0.006876592893361950	-0.05715240119184940	0.14663314117484300	-3.283381122520400	0.1015605614542300	96.91917523355690	30.634282073164400	-0.39607961592791600	-12.529299655466600
ATP_66_1	10.53	18.57	0.31117573738259100	-0.48726972419502400	-0.16384908139732100	-0.7900748752821630	0.28528047892673600	0.13135015127946400	0.3441014364682090	-2.811476558830200	0.021916922163342100	94.86786585069270	15.828190820148300	-0.30806969481653000	-9.960519887850470
ATP_66_2	11.23	24.5	0.586362464128491	-0.5424085673825540	0.08278521070020750	-0.6737865045643310	0.6144990949936950	0.12188827301636500	0.25250560546048400	-2.847482632912500	-0.1363918812892890	94.23430873544360	20.44819545520500	-0.19955140653932400	-10.877573208355700
ATP_66_3	10.56	21.34	0.4782909724926610	-0.9190722874059870	-0.5390667065176190	-1.0087577567800000	0.33612795122030100	0.299293164153381	0.476477696405794	-2.7343120881425100	-0.009146247558241010	91.65033203891280	14.053915052760000	-0.30199548598870120	-9.589161698527370
ATP_66_4	10.6	22.21	0.461114448648114	-0.6937956389733240	-0.48560422384862200	-0.9777949045400870	0.3914117738081870	0.2151996282298320	0.48116721864302900	-2.7446048673422400	-0.03454015925555820	93.33762131597420	13.485624859681700	-0.3102273263815630	-9.459300106842030
ATP_66_5	12.11	23.03	0.6147943644552260	-0.6427794137384410	0.04773157471212620	-0.3745158876183720	0.5344392417471950	0.16023481923343100	0.2592379426778710	-2.946331594833790	-0.09620493408171620	95.32817587854510	22.728390040714300	-0.19030362977069300	-11.301205223994100
ATP_66_6	10.58	21.47	0.48344822197575300	-0.58412527345578	-0.12680233428440300	-0.7565401572361780	0.23816647344547200	0.1590240745669510	0.32970309782845000	-2.820363610281060	0.035594718944999800	94.76662559894150	16.60886940115210	-0.29847514164034500	-10.12623973762640
ATP_66_7	11.67	23.83	0.5282004834126530	-0.40044303093032600	0.0346421074501298	-0.6225965766851990	0.4802369271772160	0.07865169638009580	0.2875433876037330	-2.867550499399790	-0.06501709337083180	95.83631663799440	18.936138203544800	-0.2152570531943950	-10.582114891383200
ATP_66_8	11.43	24.37	0.6164843832311840	-0.3556097972034170	-0.07310529222522110	-0.8027507614533870	0.30699161009249500	0.06519081920915160	0.34619810814946700	-2.808994334000860	0.012032526360822000	95.83796959789700	15.08368419317740	-0.29147215067362600	-9.7996785078948
ATP_66_9	9.459	23.93	0.31220302793449400	-0.7305424681152010	-0.44600229232857500	-1.1181836356743500	0.5692676241455550	0.22528740659009900	0.45942522247667	-2.714394663937580	-0.0696419569478944	93.3984316110865	13.116389638343500	-0.32633419579520900	-9.38246809568544
ATP_66_10	10.8	30.96	0.3605848265005770	-0.35114830989540200	0.5912870432438810	0.7311053810352830	0.05314016006922300	0.020088642926918200	0.056224912714750900	-3.3499928966975800	0.16765274588104900	99.01987069428640	35.1381463828925	-0.32651520059293600	-13.250175754369500
ATP_66_11	10.61	35.39	0.6188173523654380	-0.314298764794989000	0.11724778646365400	-0.3587487875556860	0.39000746318545700	0.043520375860986300	0.26125620097101800	-2.969255971664560	-0.001254774583713540	97.63815111101670	21.763508982247800	-0.21484340362414200	-11.115505795990300
ATP_66_12	11.3	27.44	0.4740389077283560	-0.5193172656380810	-0.37560184166963600	-0.852176576380401	0.3671605789134810	0.147565427057296	0.4517727926065630	-2.7862451712034800	-0.025561206100815800	94.87972017558870	13.99464542825250	-0.2722225544834200	-9.562794793831080
ATP_66_13	10.87	32.77	0.4433567445686430	-0.06610173607364780	0.1966939056563160	-0.34548010415766400	0.2172183092079520	-0.043842254585275400	0.25241287003009300	-2.9826299432992800	0.08802537379635980	98.22777209583740	21.124227699822700	-0.22937284085203400	-10.941960776552200
ATP_66_14	11.79	31	0.4328034551293500	-0.2482075295657790	0.4137811246842690	0.32124896856246300	-0.13239159568072200	-0.0061465647089731700	0.14996874197341100	-3.2030838127895100	0.23621474438817600	98.74027281323930	22.202497389422000	-0.5549659018945690	-11.14598303997280
ATP_66_15	10.47	22.95	0.6182390021975620	0.07543325799100210	0.09844915252404480	-0.5671061071559420	0.056088548694287900	-0.08604559299388030	0.30660437627488900	-2.8981380872102800	0.14288608653643900	97.3652280853071	12.44609419759230	-0.6392753984648570	-9.223577957443090
ATP_100_1	11.11	27.6	0.3368077851619160	-0.32958458156844900	-0.5386060390812260	-1.0393237338629900	0.26478345892420600	0.11185651921803400	0.5201783916017770	-2.7375244918631100	0.05248929589145930	95.35461828165930	10.934042361273300	-0.3513373456085740	-8.854081113218950
ATP_100_2	11.11	27.62	0.33712139683578900	-0.3297924681856410	-0.5404680097222150	-1.0380378662151600	0.265938571916645	0.11195671592015500	0.5210748129519030	-2.738033734857700	0.05220102547328620	95.36351153606970	10.936879910645600	-0.35108002562683500	-8.854768935007630
ATP_100_3	12.2	34.3	0.6701268328307350	-1.1117979940832700	-0.05411048442351370	-0.36013650183141800	0.49432825114452900	0.3215389793488180	0.25981141300413300	-2.956104975056100	-0.07076575559461800	93.50079759942300	23.89216248676150	-0.17154601821689500	-11.515971964052700
ATP_100_4	12.21	35.06	0.6303655231200110	-0.9164957896930400	-0.003788580764824030	-0.3610763528330950	0.2959846606563040	0.2511703139601660	0.26007802261225000	-2.95874037541609	0.018220882635243600	95.10496926008000	22.519595997369300	-0.19897182965752800	-11.26820640520350
ATP_100_5	12.54	25.84	0.7422825773834440	-0.35341443248041500	-0.22194275559021900	-0.8904108205161290	0.38511089157713400	0.07293620955224540	0.41458306644449400	-2.7678272142010600	-0.04461650918459950	94.92675510157960	13.587475830138000	-0.2472507727916390	-9.466844700797600
ATP_100_6	11.43	27.76	0.6351155706407660	-0.5704847266891050	0.0796760082319434	-0.41840220584731300	0.33178731977949300	0.1554031480889460	0.22204609869764200	-2.941563149398040	0.0019844916347613900	95.71823926278390	23.481558891241500	-0.17459214152813200	-11.431131803187800
ATP_100_7	10.61	35.4	0.6180154741598800	-0.31425286819522400	0.11762852187342600	-0.360610955014284	0.3896033200910660	0.043523699042895800	0.2610492923170460	-2.9686628611212600	-0.000993572324454045	97.63719482795080	21.738783268380300	-0.2152040067269300	-11.110999577390400
ATP_100_8	11.3	27.44	0.4740389077283560	-0.5193172656380820	-0.37560184166963600	-0.852176576380401	0.3671605789134810	0.147565427057296	0.4517727926065630	-2.7862451712034800	-0.025561206100815800	94.87972017558870	13.99464542825250	-0.2722225544834200	-9.562794793831080
ATP_100_9	10.87	32.77	0.4433567445686430	-0.06610173607364780	0.19669390565631600	-0.3454801041576640	0.21721830920795200	-0.043842254585275400	0.25241287003009300	-2.9826299432992800	0.08802537379635980	98.22777209583740	21.124227699822700	-0.22937284085203400	-10.941960776552200
ATP_100_10	11.79	31	0.4328034551293500	-0.2482075295657790	0.4137811246842690	0.3212489685624630	-0.1323915956807220	-0.0061465647089731700	0.14996874197341100	-3.2030838127895100	0.23621474438817600	98.74027281323930	22.202497389422000	-0.5549659018945690	-11.14598303997280
ATP_100_11	10.47	23.12	0.5748610229387180	0.07306381693950390	0.09415142215164010	-0.5499905255156590	0.05760798793590680	-0.08497754724418100	0.30822673749450600	-2.90289277519095	0.14014234110244500	97.34745134002470	12.519425535524200	-0.643458502202625	-9.240060641364580
ATP_240_1	12.65	39.54	0.6838782912678240	-0.36822771390940000	0.3207138191611460	-0.014060793581596500	0.005479524146318390	0.04449612958253030	0.17747143819099400	-3.0570668004932700	0.12500117329905400	96.78158086525880	19.9638663935686	-0.4049374510648630	-10.764897947550800
ATP_240_2	12.82	55.71	0.43879909234263100	-0.3340101620209200	0.41329436794983600	-0.18061746358581700	-0.029926826274303300	0.030266892226452000	0.12805654824006600	-3.0336369173370100	0.2114866797154960	97.27650731089300	18.444222180058600	-0.3322123054346290	-10.463973798559400
ATP_240_3	12.94	36.98	0.7776781220336200	-0.3097029291908370	0.17723058211820000	-0.3888640791058950	0.11070948059431900	0.03389803063078370	0.24261082348649100	-2.918481351960890	0.06607344835888680	95.51331722900020	16.081696461758300	-0.3611667167986540	-10.012457344055100
ATP_240_4	12.04	62.79	0.46363704006521500	-1.2203523063559900	-0.18775221327935400	-0.08854805744063700	0.3597491136654410	0.3594063730239720	0.30102458688415800	-3.0291461194252000	-0.028722138916925700	92.97427318539340	25.310716065970300	-0.10548706586070400	-11.758510515913500
ATP_240_5	12.26	26.39	0.5726420630789940	0.8894335131829800	-0.5798654672499640	-1.0910773859239900	-0.0731991727065293	-0.27725583346064300	0.627706022948468	-2.706493079057760	0.16113286199513400	96.41379486522580	3.353250460042540	-1.1719232253222400	-6.493335129133510
ATP_240_6	8.153	69.5	0.4801863034869810	-0.2836134510889770	0.11731456283817200	-0.5899690482963750	0.10182719806999200	0.04734933197518950	0.2435077108326440	-2.8766748942178400	0.10240561305780000	95.90071321664160	15.80004938737770	-0.417882006979157	-9.962077335008160
ATP_240_7	11.37	46.11	0.4035741411554070	0.4727500616584730	-0.31884050354998800	-1.1153510003858400	-0.1659812954801490	-0.1906344213930500	0.5297851715650920	-2.7097225418158500	0.24408980862356700	97.27468702458890	4.66381290844252	-0.8119205179473640	-7.058213851180910
ATP_240_8	13.26	42.66	0.6283469057824600	-0.6916767428422450	-0.39247521608040600	-0.9516605903190840	0.3011273687117440	0.20018995171291200	0.44442396534297100	-2.7304000500103400	-0.009177884925729160	93.07835942126080	12.784589375086800	-0.11092779296702000	-9.2835773917732
ATP_240_9	10.24	48.12	0.6274318327734160	0.28824441239057100	0.1409955772896180	-0.6705034747585830	0.584576659486530	-0.15170692541766400	0.29074425036830300	-2.8091898271951800	-0.18912436427555700	91.90493443531540	14.299030547543300	-0.27292442532927000	-9.562530893261110
ATP_240_10	10.12	57.33	0.4938643546657750	0.0876399625570921	-0.006307446170857940	-0.4768698454394100	0.02674285192167430	-0.08415943454004870	0.3536164964816910	-2.911790454587350	0.13004630640788800	97.28916379851360	11.83020304616550	-0.605499908374181	-9.03105654060622
ATP_240_11	11.67	42.09	0.4519605524022210	-0.011262554120174400	-0.8877202353128890	-1.214390772139170	0.1218941032984050	0.01216953479513890	0.7259983420897100	-2.6359388357392300	0.03960065930541460	93.88800638111070	5.728354031878840	-0.5802674637855010	-7.421120764865520
ATP_240_12	13.71	47.28	0.5112729162345040	-1.0139872854711900	-0.09320502477532870	-0.08279689057829630	0.22352944704408300	0.28432393671065	0.28987809819925100	-3.0284691290813000	0.03453753660810110	94.37886369533250	23.48653933016400	-0.06715860066944490	-11.43171696732310
ATP_240_13	9.897	42.51	0.5740417914348530	-0.4197267847310840	-0.21720877571542100	-0.9613908547291920	0.27431962195035000	0.09744785163384500	0.4024352864165280	-2.747791686956540	0.021647599648321100	95.03352866449530	12.863797744215400	-0.3205115177537680	-9.31299488504753
ATP_240_14	11.28	36.08	0.4771199733884420	-0.11145226098659100	-0.1135233243708750	-0.6427242279251790	0.06603570275236720	0.00012030506551452200	0.3740798684853570	-2.8414002681422700	0.08202499687799200	95.83211850683390	13.390596210747400	-0.35200856192145300	-9.398131348956270
ATP_240_15	9.098	48.75	0.49754180368232100	-0.0207117428306351	0.4935355104833820	-0.2803660037924690	-0.41852447266584800	-0.06542698510268480	0.11129648836026400	-2.982190280496600	0.3327676063576110	95.97238179541600	15.384253996266400	-0.7764759685180130	-9.815134528543910
ATP_240_16	9.535	39.72	0.7093038277125550	0.08862778423554	-0.3460375856490860	-1.1550165355683400	0.15707420927631400	-0.07314714652953910	0.5167141197247220	-2.690303390174260	0.08807802107548490	96.06372107486150	7.375943129756700	-0.5898328585310360	-7.927911952704100
ATP_240_17	11.27	67.88	0.26709936187145100	0.0849966282699521	-0.1640648935208680	-0.6525599520774470	-0.51195194805767	-0.05763833122792990	0.40742959738995200	-2.863516991567040	0.39023059113023100	96.99225677103940	7.84333051323388	-0.853375720613049	-8.067388281656240
ATP_240_18	11.55	42.05	0.7106685316763310	-0.2554139248714980	0.14438783441135800	-0.5207270251154350	-0.12203128417425700	0.026017398522236200	0.253381072255989	-2.8814360014962900	0.16844347269880500	95.49841051421930	13.372973905106200	-0.5196110371805340	-9.437167471485050
ATP_240_19	10.28	72.29	0.29248481671052800	-1.1767192161218100	0.16252638817139000	0.04302634496530230	0.4813650490385270	0.33411756233693200	0.14788718292334800	-3.089256947973140	-0.053676477120504400	94.27267631199180	29.541559786859000	-0.17649070189373000	-12.47338342232820
ATP_370_1	10.73	76.33	0.3612529681590910	0.18224174297226200	-0.13571342208112100	-0.24265739266794200	0.09663193067773840	-0.1088803470530950	0.4286353986433550	-2.9630709239349400	0.025285362272342100	94.97840959879170	16.093263927861300	-0.3192745850958260	-9.81831527813196
ATP_370_2	12.74	77.94	0.4760268219224350	-0.9843422444222140	0.24328028075446600	0.4512942310609710	0.20060360374783000	0.27526221900777800	0.10314287666284600	-3.2056714783430600	0.020758889883123500	94.73677944220810	33.77667605437220	0.004128345162317980	-13.128930438398200
ATP_370_3	11.46	69.65	0.367743971768323	-0.7305543933611740	-0.2145922876334880	-0.3679236573331150	0.29116777245566200	0.22467041806267600	0.3372079508219610	-2.9417613487364900	0.009858765916211860	94.81730349654890	21.26828402821980	-0.059520748369690200	-11.020515418206000
ATP_370_4	9.38	54.85	0.5022884868234680	-0.04757680211545670	-0.35200728192146400	-1.0437077701150900	-0.4034830905220600	0.002650130334991250	0.4622131048824450	-2.712777061474550	0.29920986035780000	94.14073311697460	6.622236750080020	-0.8399708289573300	-7.759364914590030
ATP_370_5	10.09	40.95	0.5967171767414350	0.05086883076362470	0.1751974009866400	-0.6084362007336490	-0.1615741554918520	-0.07886949583315470	0.2626850333292960	-2.85858927419163	0.1949550124317720	95.49824384701240	11.051898237443100	-0.7424540499263730	-8.901334232322900
ATP_370_6	12.02	71.85	0.3968158224055540	-0.9500060879736190	0.3415205570491860	-0.2638357651161000	-0.038296360262594300	0.2669153624161650	0.06461340283692020	-2.9765819947373500	0.1506017866464280	92.48301902274720	23.867054908411100	-0.11025024996832200	-11.499709651448700
ATP_370_7	9.78	50.33	0.28717861163846900	0.24924687900080600	0.11186186913053900	0.026341662052359200	0.15973591222847400	-0.12067666613635300	0.2854066326298070	-3.1036389932761700	0.0984358684092513	97.47338034608130	25.226683765554900	-0.24867631222100500	-11.594867715475800
ATP_370_8	7.217	55.35	0.317606899104276	0.7643660158247100	0.1634933579133000	-0.43507328516243300	-0.18659368748714200	-0.2943275389063370	0.306960841901903	-2.92052214859613	0.1876427683600240	94.58702787450080	9.062336303545400	-1.1869103472177000	-8.28600250053321
ATP_370_9	8.639	45.03	0.5390200060300330	-0.08126655279156980	-0.09318653957747740	-0.7654262596049850	0.8198175531109340	-0.02174652827365920	0.3735156094585990	-2.8188968338554500	-0.20779654269422500	95.45479562137520	15.209181613966500	-0.2783355059059040	-9.820280331125300
ATP_370_10	12.35	47.16	0.6735322226082210	-0.645996894409744	0.27569883946115800	0.021648663275143800	0.20842208982705500	0.13793360796126300	0.16673115144108500	-3.0634216409033700	0.025028557668156200	95.85770748054530	24.90821928493740	-0.21199289980332250	-11.683417118177600
ATP_370_11	12.91	48.64	0.35604325522992700	-0.3927475513254040	-0.0601176774662732	0.32491300944565300	-0.260698113151208	0.06019264344089030	0.3526571513448990	-3.202509568282240	0.29723956787171900	97.58905628298960	18.625864207430400	-0.5020165749871880	-10.442604027602800
ATP_370_12	8.75	95.51	0.29635779818109200	0.4804278877098170	0.4550891616904270	-0.45277268927417600	-0.363555558478322	-0.20325460233015600	0.13559335281208500	-2.942938795778900	0.34440578918872300	95.7930940681552	12.26932604143830	-0.833946100717978	-9.027295673685790
ATP_370_13	7.424	75.88	0.4760890198294950	0.15049467744132500	-0.3925732173689760	-1.4022720121772200	0.04430984337075990	-0.049556777863711000	0.4918738548442340	-2.60897776317937	0.1323498570554130	94.43118921692420	6.225822181276310	-0.6592120823443130	-7.585351030086360
ATP_370_14	12.52	51.07	0.5665560184768020	0.6144339857187860	0.14341489209882400	-0.24192420066792000	-0.21804822353984300	-0.2620417947739790	0.3307247426404750	-2.979550857667360	0.213088545012564	97.08570507476260	9.446177529062600	-0.934288695164033	-8.373112965798170
ATP_370_15	12.82	59.1	0.3871545641683520	-0.6206978782582720	-0.9862318218727400	-1.0028949324387100	0.28312606316609600	0.24639017030894900	0.673702096096761	-2.7191322885536200	-0.015477035236892800	91.5148052858022	10.08316546481820	-0.09764435517330120	-8.624738360726710
ATP_370_16	11.69	58.09	0.337274503642643	0.3284848899661720	0.5727654899991420	0.18159033633928000	-0.10500147168797200	-0.16870056688551400	0.07795356811372110	-3.1462950782817500	0.2032968029360510	96.71696554061440	26.776702496899400	-0.233187962292891	-11.844308181865300
ATP_370_17	11.43	55.32	0.21186680701022100	1.5557469018939900	0.012081597706716200	-0.2506184400868380	-2.395630013375230	-0.5538923627968080	0.4410654597303260	-3.001530633046890	1.1920978467942300	82.13143153230540	3.8114814683791400	-1.7649356637397500	-6.652392212648010
ATP_370_18	11.82	39.93	0.5250195669015800	0.6824218846536500	0.20344378732784000	-0.6658782383374740	-0.4158266035307240	-0.29939802564969200	0.32469291135231400	-2.8823796937212300	0.39455776980022900	97.8553151611258	8.850039899850970	-0.9909081619628260	-8.15341042686332
ATP_370_19	13.68	36.98	0.6364374300173630	-0.4306705929976230	0.47631669683536600	0.28325918062589500	0.07306327613509940	0.06752120441225490	0.09999363728148980	-3.170793178591540	0.12387861138748800	97.7197877710327	26.82487635265250	-0.226151549640591	-11.979074521968900
ATP_370_20	9.591	55.43	0.3692322836181040	0.9564407758737250	0.5815481485715280	0.08738796292094650	-0.22941724565009000	-0.39754099925745300	0.16777318335809700	-3.076187219886410	0.17803609038460700	91.02878931807590	13.911312723250400	-0.8383220661715460	-9.472173019553000
ATP_370_21	10.61	84.2	0.39688314048436600	-0.5107590236528210	-0.025714421033862000	-0.1294351552027730	-0.021985447622565100	0.12791416092217000	0.2869011277133900	-3.0093381678030700	0.10914917726773200	95.47325671210540	18.792828323288200	-0.3271671644530900	-10.552502645349300
ATP_370_22	12.14	78.23	0.46927232396177000	-0.8190956260123880	-0.20266457339856800	-0.06604602925313720	0.10232052275685600	0.24999300019800700	0.32667091313874900	-3.0339634579512500	0.07005671699226060	94.80470940334690	22.372824711917200	-0.05963973676229480	-11.226018280410300
ATP_370_23	14.05	81.19	0.3554120609913900	0.13292902875570700	-0.12755690035514600	-0.09934276677191120	-0.3400761924376830	-0.08685313356589170	0.3987696007340890	-3.00409815947375	0.21687541674868100	95.47302055130060	10.898511795121300	-0.3886753061220480	-8.647790117194940
ATP_370_24	12.6	65.26	0.4879305292361640	-1.1192680888656100	-0.716124952925241	-0.9052119432995930	0.24522360223845500	0.3777160225357180	0.5321215116472540	-2.7402203334394700	-0.017313490023139800	89.49442958128870	14.073523496825500	-0.0011122104858655000	-9.57170493190962
ATP_370_25	13.45	82.71	0.3686852523471450	-0.30076854440402800	-0.6508921469074730	-0.6655809446904230	-0.25104541177047800	0.08214079363248880	0.59689105626061	-2.839562624077190	0.24361384411154700	96.02181358897690	7.536114964485790	-0.20773434703018200	-7.9635343986383000
ATP_370_26	11.86	74.6	0.26738470196800900	-0.5594197583913160	-0.37592669472688600	-0.023027449291082800	-0.014125650017616500	0.1798611348514590	0.41836335495002000	-3.0275185597613500	0.06996237655979040	93.41678928746000	23.072862563313700	-0.003648191142434180	-11.29657379231420
ATP_370_27	13.4	75.45	0.4595367348969080	-0.2872211631122850	-0.7306282548819510	-0.4756329279723450	-0.050880004305802400	0.08850434445664440	0.6219316783115370	-2.875139258599830	0.11151634962462200	94.76613089071960	8.29565707374109	-0.32651505201607800	-8.180803933492400
ATP_370_28	11.51	50.08	0.6582280418210810	-0.013894339547017200	0.09230217229050420	-0.4660423457950910	0.1004471364508040	-0.06017769681861 7200	0.3002271329135370	-2.888949557153670	0.05927908015304090	95.27666023202410	12.044736465784700	-0.5783525717688630	-9.128169955952170
ATP_500_1	9.056	122.7	0.4143245039116710	-0.18305675359736600	0.20980416721781600	-0.11377849890468200	-0.17377631659914000	0.020664354700557700	0.20020494030939600	-3.043430449036150	0.21662321614147600	96.3703182983821	24.11787611427370	-0.2364134629051160	-11.393703674124300
ATP_500_2	10.32	91.4	0.5374967356026350	-0.21336466657069500	-0.23043147229606400	-0.46927670442602000	0.07240646438654610	0.03789941428938890	0.41639397061771400	-2.9221618275480800	0.11718646616153000	97.16603927843100	13.332709344915400	-0.357076600981526	-9.407751868690880
ATP_500_3	12.35	95.15	0.388543546230607	-1.179888597308470	-0.7717408073134750	-0.20223105617347200	0.3988023798478090	0.44109823861606000	0.49963295097504700	-2.975427681826380	-0.10463672508599400	87.74100468930720	24.879012587380200	0.19961246969668600	-11.663618544036900
ATP_500_4	10.9	104.5	0.2911992422102050	0.26543635634461300	0.13679692177278000	-0.09650206682954640	-0.05167739717083550	-0.10188787637961400	0.23282752359080500	-3.0518679733737500	0.17673249205275100	95.93649489159350	18.748052139217800	-0.28438454114689100	-10.345031031256300
ATP_500_5	10.36	90.96	0.46476498715606400	-0.2868841238786590	0.36478786853549500	0.37708574648945900	0.4710765494873080	0.0584671864988973	0.10212999437894300	-3.185661320322590	-0.1091320992336810	94.72805624668390	36.936740861307000	0.028933650273152800	-13.585055228744400
ATP_500_6	11.13	117.1	0.38081943090916200	-0.5830019219264960	0.20634836466281100	0.42438288298160900	-0.04479734227220230	0.15191144097887500	0.16362131505109900	-3.2029574287326000	0.1277929334276410	95.34753817282050	25.27938988844870	-0.24481790202848100	-11.700668459646800
ATP_500_7	11.47	123.7	0.2589162416715600	0.6371357970618330	0.2626587082136880	1.205338081091510	-0.56872141389371	-0.2873254642471880	0.2955519056295900	-3.4462216149933900	0.2853771635583470	87.90809691816920	34.09296417816270	-0.04548558644131430	-13.043499124410000
ATP_500_8	11.14	93.86	0.49655644312252700	-1.2216486836892200	-0.42942077571488500	-0.5887486920652960	0.009695759823390150	0.40512261773818500	0.3828520467658840	-2.8634307389810100	0.10860325611293700	90.16517024310360	18.057458619620300	-0.06452987644240740	-10.412367934268000
ATP_500_9	11.32	90.3	0.4419034685404530	-1.1368515522263600	-0.5451814438105380	-0.6937151526323810	0.3637522424820630	0.3745127737903610	0.4552398714871360	-2.831278409139800	-0.037003781873919200	90.93401502574490	17.031197091240500	-0.01661261541579790	-10.200222218849300
ATP_500_10	11.05	109.7	0.5010103912379	-1.1737674821594400	0.10174278423240900	0.3824363042821110	0.2571698114209320	0.3416787707874280	0.1699172802127330	-3.204226968679900	0.028498933467042800	94.63968546692420	32.58331138579750	-0.06407856789614370	-12.947651332734500
ATP_500_11	10.53	74.11	0.3473468701221620	-0.4022422111033970	-0.8761977150788310	-1.088721628136470	0.125791148890242	0.1615463316780270	0.6587015802967680	-2.6758224241456600	0.009707157213251560	91.21352817504460	8.728227390904400	-0.3208650838586450	-8.283744900138820
ATP_500_12	12.19	83.17	0.5279572343896770	0.14009467432079500	-0.326272740666658	-0.01613963840349370	0.14630542441978400	-0.0829399314487309	0.5013608358970610	-3.0510366456314700	0.04677092965398590	96.39350538707060	11.332985939265400	-0.6141141081935950	-8.877429077209830
ATP_500_13	13.3	70.31	0.47338920791392300	-0.17539552778699700	-0.3324651760155760	-0.14568242916746200	0.2477051322221380	0.035099253039360100	0.46024274785865100	-2.9725021102197900	-0.07580152357341340	93.1736663200894	18.455389629257100	0.023011933395220400	-10.319885757264800
ATP_750_1	11.41	102.1	0.3763035253027610	-0.1362902105997050	-0.3369058620926050	-0.10060153881890800	0.0554111690642525	0.04379286537840410	0.42810217791207900	-3.00240731283668	0.060331276566822500	94.96125680961230	17.507974547285500	-0.17737116372549300	-10.09919874690510
ATP_750_2	11.56	94.04	0.5012587414604420	-0.5578597183752530	-0.3572540003995710	-0.07547417713403060	0.04661224055124150	0.1747515341802790	0.4133864917458570	-3.0180356434756	0.07295168447921980	94.43104883797940	18.085210458928700	-0.17009850993053600	-10.402118102689800
ATP_750_3	12.1	110.3	0.1580989343285740	2.3409589696699	-0.3309200254103470	-0.2943265362303150	-1.4489946627666800	-0.7369875560832090	0.5760712088974090	-2.903061622008900	0.632992767735629	91.32969387441740	0.8177805998896460	-2.512361011192100	-5.290924739903920
ATP_750_4	12.77	59.99	0.46472649020875700	-0.058722155102608400	0.2898851463583480	0.9066678145570410	-0.20301099889823700	-0.040319850620861400	0.19090050577035100	-3.32295814719268	0.12979007381625000	93.97621826631200	30.33196981477140	-0.19274769444669300	-12.465407074761100
ATP_750_5	12.35	59.98	0.8067508748618030	0.29204775496820300	0.0925993807514079	-0.7443996980797590	0.005160005171580850	-0.16824644836634100	0.34199448230500900	-2.795492841978690	0.1012506787847260	94.93904438657500	7.6808811556782200	-0.6562321023294910	-8.026031029670570
ATP_750_6	12.19	55.34	0.6311582418500550	0.1954740124252270	0.4080457553536410	0.2868391799449520	0.2272046239821280	-0.1327769558465300	0.16357272334028500	-3.126281611415720	-0.03486244399896850	94.44565356429420	23.489670492112800	-0.24833472858574000	-11.258166299445900
ATP_750_7	11.94	97.39	0.4462086810783040	-0.23187830503756700	-0.2663762617240920	-0.015814437360436900	0.015583093249787400	0.06817390486392550	0.39570421244826000	-3.034089723868560	0.07046307835667110	94.59592531843350	13.955951707406600	-0.4104100212635250	-9.50017165295672
ATP_750_8	12.56	88.97	0.33691314814180200	0.3797089041718020	-0.29705821056852	-0.4227623346968240	-0.30802672554024200	-0.11161072309243500	0.4350112129720100	-2.926446724409310	0.2714932337433950	96.78000536154000	8.676829335574480	-0.5833600657822210	-8.023309939979460
ATP_750_9	11.73	103.3	0.2961000705065750	0.5051092755498240	-0.5284601006062020	-0.6301922906228160	-0.5843898057510190	-0.14451741083848500	0.5600418448309620	-2.8226373211790900	0.34052322031975200	95.41068168090460	4.123999191015770	-1.1155782766404200	-6.897506405341280
ATP_750_10	12.98	97.77	0.4657171522458820	-0.6016610620674020	-0.34031888064629500	-0.22833271341925000	0.14564628457128500	0.17721037602672200	0.41217048175457300	-2.9912297102278600	0.07923764254665840	96.55318479501780	17.64731883551050	0.1067360904240650	-10.305051445045600
ATP_750_11	10.95	94.5	0.4617394789142280	-0.6391239031243670	0.1973671424411880	0.2569252882581060	0.29193341138134300	0.16001352427143300	0.170397304984503	-3.1414430376051600	-0.015519279328928600	95.79736104088430	31.333896581843600	-0.03275502429104700	-12.745860559642200
ATP_750_12	13.41	48.43	0.3985924096838680	0.6254851820490380	0.3582188208699690	0.5302082816682920	0.6502205536028700	-0.27436635672441600	0.23381633586821500	-3.196180124890740	-0.25934966039053600	89.70913346035280	28.70921337490590	-0.025733707153539100	-12.171493724408000
ATP_750_13	9.754	108	0.31584709931349500	0.9463082754560270	0.024067944428922600	-0.2492679718630360	-0.038176901597262100	-0.33601505789015200	0.368502202913187	-2.953492699872010	0.08220943590402810	94.27715165962850	6.980752601767260	-1.1065790639316800	-7.609596658370880
ATP_750_14	10.51	59.09	0.4518315429098850	0.8760595458666030	0.4789076528970840	0.06078544800431260	-0.24991487701020400	-0.3958720909678370	0.23936030704392700	-3.1098461736031600	0.2781227468153820	96.3419915891043	12.373770466412600	-0.8623267944091440	-9.112733021165840
ATP_750_15	10.46	77.93	0.3240041107897150	0.36346541882065000	-0.1259905162288550	-0.5919224276225090	-0.2539045837167650	-0.1458916385689750	0.40726527230241400	-2.8680760351038200	0.22763313486431200	96.40291313218770	10.70346734276230	-0.6220254741383420	-8.57387800492667
ATP_750_16	11.49	99.88	0.3393668124174510	-0.4212022917733340	-0.32087049456547800	-0.39148985183713800	0.3084716089256620	0.16121452455698000	0.3603871293829960	-2.9272669664998100	-0.0036509090461173800	93.87049719845780	19.411356194076900	0.08986077505886510	-10.636606246359800
ATP_750_17	12	104.2	0.43810701992123000	0.20321120040285900	-0.2859547188810460	-0.6727372524061810	-0.23320902376728900	-0.08830028872664530	0.45628467532348800	-2.8342343948930400	0.2341262183515000	96.85105481839000	6.989333544577070	-0.48045076196786500	-7.701497456790550
ATP_750_18	11.64	112.5	0.41212649900690900	-0.5362193907187670	-0.6273756258713210	-0.540139328546395	0.0886721457467665	0.18353110463105300	0.5372546494679140	-2.8508881208173200	0.05456454821062880	93.81850767538620	12.381396558847	-0.012506242571553500	-9.165734065182000
ATP_750_19	13.35	68.1	0.4471154337169990	-0.49383655956078300	-0.992394961810106	-0.5223246439622160	0.020534637125076800	0.20696558922894500	0.6766793095067440	-2.8753360517442900	0.10258529913576400	92.72722477621540	12.738638752601000	0.03836604250936010	-9.217342051920370
ATP_750_20	12.6	112.9	0.3129677899408620	-0.5626712150893200	-0.3330940251697090	0.09185307735526910	0.1063908050827020	0.1743642957909050	0.3920369980250060	-3.055805166638820	0.018961850952022600	93.56917529943110	24.191684922968600	0.2481500799341860	-11.463450208869400
ATP_750_21	11.49	100.8	0.34732566586323200	-0.8788820391288700	-0.077100118680551	-0.4382230898872120	0.4112428539740780	0.26117180792897100	0.2636471741241130	-2.8939585514312500	-0.0800945871991763	91.73227572010450	21.15387596488140	0.06517436142297870	-10.995484066810200

**ATP_750_22**	12.19	101.3	0.34748947309179100	0.2624780432704310	-0.004018754375988450	-0.10879929496117000	-0.1742947483233710	-0.12164891539021500	0.3311203319457420	-3.0324281038585100	0.2267197319033150	98.16149606725590	11.973457511849300	-0.521554530448604	-8.926664567902330
**ATP_750_23**	9.59	114.6	0.3272510659615260	0.5435452744626470	0.4497244195344020	0.4494721847856910	0.25629028731433300	-0.24926927332066700	0.17474413874706100	-3.21044067034501	0.00510803962665618	95.69475147759760	26.276056598401400	-0.24540312262290600	-11.75660728230910
**ATP_750_24**	8.756	127.7	0.3057631804924560	-0.7140583939172230	-0.5078384500274620	-0.9944840087953510	0.1083212030618890	0.23082718754948200	0.4738822138979840	-2.752358946802700	0.11965530822216100	94.26482572125080	12.30557591873690	-0.2585951810853920	-9.183709320123010
**ATP_750_25**	11.36	105.4	0.2455561816291210	-0.4312615599449620	-0.2929973274003600	0.02676457251143270	0.3106294770013730	0.1432189729161830	0.3693631339874290	-3.029841540668250	-0.08866817717510750	91.78228118647730	24.60771596205630	0.10583449840812800	-11.571308349815200
**ATP_750_26**	10.06	91.3	0.1874421863953730	2.1126586093524100	0.11222246407942200	-0.21632872775753500	-0.8960652028493280	-0.7340872699161130	0.43295999804635300	-2.9661436107577000	0.45026192136495300	90.80882786256280	3.000698091390520	-1.989893833675530	-6.336370942905430
**ATP_750_27**	10.39	72.03	0.5037548088511010	-0.6274291306902740	-0.6782522161955930	-1.4615751291853000	0.05044908986491880	0.22627347742911900	0.5474397118622660	-2.56016786513688	0.0790843216583427	89.22320685814650	8.469860664186670	-0.32648764476488600	-8.232460857062790
**ATP_750_28**	12.7	93.82	0.4451530759788850	-0.24373093718067900	-0.27880694581307900	-0.27876655401085300	0.06439063839110600	0.07929255802487750	0.3836070829569020	-2.9247370597052700	-0.0025654174092915700	91.50469369564800	19.039135122168200	0.12552953917406200	-10.445161503916100
**ATP_750_29**	11.24	92.28	0.4064398797209570	-0.7762723440029370	-0.12547336843929700	-0.2547908703925040	0.37740277869951100	0.24296569680417100	0.27275204559955500	-2.9587367054105000	-0.08170883541081770	92.18497267752540	24.846292449679400	0.04216289686203300	-11.658248457188900
**ATP_750_30**	10.17	105.5	0.4000223513532180	-0.1481550730016800	0.09689337833548540	-0.35283003389542700	-0.03943529744073530	0.02886158746617790	0.22643508405434500	-2.941080487966680	0.13925549139668300	95.00915896707690	19.680971219931600	-0.15513981540156200	-10.585907119071300
**ATP_750_31**	11.62	117.4	0.3399157187695500	-0.5192703373526950	-0.5864835947620100	-1.0796257377162000	-0.1481722023932400	0.1670481114641950	0.5306031105503580	-2.707325943559600	0.22264207869581100	94.28375375822150	8.361267155625190	-0.08727762523748390	-8.173586631587820
**ATP_750_32**	8.398	127.7	0.17083751911480500	0.7398851428485210	-0.23904250924746900	-1.3379404083521200	-1.2673771023462300	-0.23788595610259100	0.4395327286868400	-2.6189012490928400	0.6762264881065700	92.83881557061950	2.4309795718677200	-1.6363073382770900	-6.2859690868178200
**ATP_750_33**	13.3	61.36	0.6777210787550030	-0.31324932972780700	0.019766248426143800	-0.15349280377456200	-0.03618979472879110	0.05946176189349820	0.2886098421246670	-2.987137367897120	0.10048102650872900	95.19770432749920	15.46250088730270	-0.3179425683190170	-9.847491749224860
**ATP_750_34**	13.25	61.6	0.6679314685685440	-0.25322898458422900	0.029643944284609900	-0.1296066511520800	0.03601028822313510	0.03624505442241860	0.2933361120588890	-2.992902394595510	0.061463108857601900	95.0943166941834	15.491145341077000	-0.32946295571604500	-9.851067655865870
**ATP_750_35**	12.87	73.18	0.5850264328031150	-0.931389243561785	-0.513663705056791	-0.3803607486065520	0.2600894027435940	0.29600133845170500	0.4631215368301460	-2.8923070795272300	-0.06580775356678890	90.43068391187790	19.06432430727330	0.09464649833406270	-10.591245399741200
**ATP_750_36**	12.87	73.18	0.5850264328031150	-0.931389243561785	-0.513663705056791	-0.3803607486065520	0.2600894027435940	0.29600133845170500	0.4631215368301460	-2.8923070795272300	-0.06580775356678890	90.43068391187790	19.06432430727330	0.09464649833406270	-10.591245399741200
**ATP_750_37**	12.52	59.58	0.7067573835028420	0.15806094541001900	0.13257021770394000	-0.3575011432031650	-0.06278784442979110	-0.10198486384687100	0.28140801067388100	-2.9113034042234200	0.10045513474048200	94.69032786172560	14.603298334740300	-0.39822215158086300	-9.482316896805860
**ATP_750_38**	12.58	59.58	0.7068774948315170	0.15762958759698000	0.13305557931397600	-0.3572284301924480	-0.06294079844039060	-0.10188268011086900	0.2811010131046800	-2.911240384914860	0.10066672814210500	94.69673263314990	14.585110609897300	-0.39376952211839200	-9.477717934396880
**ATP_750_39**	11.6	99.37	0.33030871363317500	0.272456595456271	0.2844660307479760	0.5044912027183980	-0.37666320665083200	-0.12337448647634700	0.18212389397968400	-3.205282337913350	0.22696066579164600	93.33318253469870	16.944947302656100	-0.465127554667063	-10.08938444227950
**ATP_750_40**	8.517	76.56	0.503739643125599	-0.354320655553657	-0.3871413576652860	-1.3751127395608000	0.308475571382439	0.10869169410154600	0.45154049426575500	-2.629713580110750	0.04341634279589740	93.83423300230790	9.326793614627980	-0.39745918581380100	-8.462928083999350
**ATP_750_41**	12.71	101.8	0.5070706836587100	-0.4967240632791270	-0.5526416174402090	-0.3773507514327310	-0.0404016811265521	0.15556911596391000	0.5176420641667610	-2.90743570240386	0.10847579430104300	94.33403024958470	11.510990394662500	-0.13524305852500100	-8.98110908887499
**ATP_750_42**	13.58	105.5	0.2607495238188490	1.0298627069175600	0.053895531990637800	0.09588157054512200	-0.5939807043845390	-0.373711190943979	0.36456268210485	-3.073382994419050	0.3312074031700420	94.44614857988790	5.849825728543020	-0.7527916221927820	-7.283880368989320
**ATP_750_43**	10.18	67.95	0.5881067945686650	0.31827506724012800	0.17368003540887400	-0.30258149433472800	-0.04878496651198710	-0.16224093333475400	0.27793941692688400	-2.946025191459060	0.11689024557524600	95.48019168288640	13.694839495298200	-0.6687440357568930	-9.317446354755960
**ATP_750_44**	13.66	74.41	0.4715404972339100	-0.28699444328841500	-0.5476597076269540	-0.2633892855577270	-0.05508600404063730	0.1019485892290030	0.5103476262801390	-2.938273735036030	0.06599399680661850	91.89946869672580	15.332864041271600	0.04208939473377280	-9.64611256957897
**ATP_750_45**	11.57	103.8	0.5126958380701840	-1.1306852699150400	-0.1792036131307720	0.35619662068727900	0.26934599960251100	0.35226066518186600	0.27906243362230100	-3.168387154480070	-0.005554857960439890	93.38502105634840	30.035082607157500	0.003910111162482988	-12.542038972001500
**ATP_1000_1**	10.51	51.42	0.4979095695039000	0.14935854841186200	0.2675893043140410	1.1011830044066300	-0.080189902916341	-0.10251818892473500	0.2135449764805240	-3.4224032958578500	0.11300132879607000	94.38134076971120	37.71904333746910	-0.20917709359432000	-13.60326761409670
**ATP_1000_2**	11.03	49.7	0.606675525075245	0.3638614394235190	0.20622522387457900	-0.3383347832217400	-0.16655164633943100	-0.16921049150746800	0.26002021797261900	-2.9481640391831400	0.1758471715591500	95.54065849035630	13.234792012889600	-0.6869217460777850	-9.211076425135790
**ATP_1000_3**	11.04	73.23	0.3957552838626200	0.16939829560666900	0.3825566796096340	0.27389933354179200	0.10492791649609300	-0.10188820998209700	0.14586882697244300	-3.188176816010980	0.127956266800679	97.80551898282240	29.906601371458000	-0.13093618056861300	-12.374835530360000
**ATP_1000_4**	11.19	51.38	0.6182485074693090	0.1424937359649310	0.11869159626284100	-0.11272908705461300	0.07869058330809600	-0.09620768998616830	0.2897957928786400	-3.0328344528762700	0.08842748167829170	97.10274471997360	19.239278158247400	-0.3745591581777800	-10.460825881845600
**ATP_1000_5**	11.29	49.03	0.6785254010616550	0.4114428997458450	0.3863072926991200	-0.14126987136689300	-0.06515977760107730	-0.20731837256312100	0.20433398228511800	-3.01941587895809	0.14137206347161100	96.00335088514240	15.281918888906300	-0.6456816944428660	-9.693472321945230
**ATP_1000_6**	11.97	46.51	0.6494529933111670	0.12735260073759500	0.33911897171917000	0.07152857047235040	0.021592673332096200	-0.11676805443414300	0.20619223975608100	-3.092825656503340	0.11341807219934700	96.91961715284370	19.610609408195300	-0.47549228938738600	-10.571152666821900
**ATP_1000_7**	12.18	48.32	0.6998185847166260	-0.04829689847093440	0.4021214434392630	-0.15090564548153500	0.12925761163084100	-0.046220046376769900	0.1402568385567910	-2.9977303672148500	0.025656887560355900	94.19871726117210	24.529903279609000	-0.14041163825136900	-11.476384602276700
**ATP_1000_8**	13.61	51.03	0.607938434164297	-0.408293112051267	0.25481704452348500	0.3953860965298830	-0.0119281343302413	0.07356805937589460	0.18264590482390500	-3.1810430300031200	0.079968697863615	95.18488633997820	23.526508664408300	-0.31120926733959600	-11.36563135510380
**ATP_1000_9**	13.65	62.81	0.6139887410784240	-0.9205940004504800	-0.012065421763853800	-0.2762495278574980	0.0032444098486863200	0.28504365661044200	0.21694623031943500	-2.9542427653083700	0.07869304355121160	91.43621393377460	22.87411067529360	0.06723367041209410	-11.31298522878930
**ATP_1000_10**	13.68	47.59	0.6662513123168550	-0.3001344375441840	0.34902658769034300	0.26954040120786900	-0.1503241743482220	0.03670858100898260	0.15064499914847700	-3.15273708226482	0.17321492817518100	96.33553082211840	21.88721957108350	-0.3467856075985500	-11.03727653454510
**ATP_1000_11**	12.91	97.68	0.39082278210414300	-0.6221191959594270	0.3491202689258330	1.1711608253484600	0.8617117040621460	0.21081465639966900	0.00549139932603385	-3.4595905960513300	-0.25148364076178500	93.15707986698690	54.164947780050700	0.24068493435348700	-15.95843284284190
**ATP_1000_12**	11.55	95.76	0.43498532045696800	-1.1316098964295000	-0.5066694633298440	-0.32111353507225300	0.516343580496770	0.4087335408893440	0.3765905928778290	-2.9408167590920800	-0.11817199773999800	89.71699858866110	24.074587881056800	0.09861037790345240	-11.527499299743200
**ATP_1000_13**	9.765	92.88	0.503288669571012	0.24204357324291700	-0.0644617752505128	-0.5437753943662130	0.23503947531665800	-0.09436448808758320	0.339473169944996	-2.8696699416983200	-0.0001720297511077770	94.93943827529650	13.681245119610500	-0.3210629275481720	-9.332772630090270
**ATP_1000_14**	10.78	120.6	0.35059686378541700	-0.00701257648314023	-0.17581558978397300	0.286174540551391	-0.15760507943539800	-0.024584652514255600	0.394567762873837	-3.145507726153280	0.16227676302240100	96.5994330585317	15.11031520838910	-0.5589295909456930	-9.70427302780778
**ATP_1000_15**	12.46	101.2	0.3833582337955740	-1.095568006665050	-0.3213178335635570	0.20684677253619900	0.6075538489525420	0.3830368089774300	0.30279379365003300	-3.1378299854255000	-0.12494164375169500	91.7640686813213	33.25554695975410	0.2216698515830960	-13.048416861259000
**ATP_1000_16**	11.95	62.45	0.6514357486218290	0.14341800921373800	0.18822863919568200	0.5946945076503860	0.05631776001350810	-0.11376977331451800	0.2699978328868910	-3.266578866200960	0.10505107207623300	97.64644869527670	24.89994156120100	-0.30436302799271900	-11.540199263816900
**ATP_1000_17**	12.11	63.49	0.7146197013007010	-0.09668604100990570	0.06837062069948930	0.048142647291585300	-0.18287658363592300	-0.01760571269678050	0.28768629033870900	-3.0535785323613600	0.15752744371369100	95.85455136120960	15.340352141753800	-0.6020953339681810	-9.761338937922930
**ATP_1000_18**	11.31	90.52	0.39998689307911200	-1.012127240009130	-0.4994836883648320	0.13067894611653700	0.656033056278796	0.3635227565239180	0.3980331506607400	-3.1080763402655700	-0.17690363060493500	90.60639077059100	31.41097882417890	0.08049749014553870	-12.757054090118100
**ATP_1000_19**	10.91	62.75	0.5566661297777820	0.020662061022046500	0.10772352031471300	-0.07379543904163900	-0.1906849898285110	-0.0457886921602906	0.2676085234008630	-3.0236411143999300	0.15971890903887900	94.8384112026115	14.672085120853600	-0.7218520684387860	-9.628388755998
**ATP_1000_20**	12.24	85.68	0.31158352060934100	-0.07427240103182580	0.10520783548732500	0.8717403877324330	-0.10197911852918600	-0.0016983348849982300	0.24377030394468400	-3.349393594659060	0.1345071729636650	94.98252000155840	34.34726174189850	-0.03225763898893060	-13.075623605933600
**ATP_1000_21**	11.92	61.69	0.6835736575633080	0.175179012376164	-0.1171481601711050	0.014120296568307900	0.03132803938758300	-0.09178095046750740	0.39366648226537	-3.058416371395040	0.08042591425831210	96.32872205110160	14.812809521232500	-0.5674776680817980	-9.575829278308180
**ATP_1000_22**	11.6	69.25	0.33880886206810800	0.27993529274613400	-0.11842555518563800	0.09344403868143000	0.11378669031926000	-0.10677606718550000	0.38016764264535500	-3.094070777600140	0.04635790122530670	95.39176735028240	19.215777628683000	-0.30439285304427400	-10.441184037792600
**ATP_1000_23**	10.9	75.89	0.5829783888819480	-0.37557564579384900	0.09006966046886100	-0.25618269828814800	0.29089515646239800	0.08092709420050940	0.2456368335937240	-2.974686530197700	-0.025561869577364300	95.15261257557050	24.483864905368000	-0.04406238623164450	-11.583459531298400
**ATP_1000_24**	11.68	69.46	0.5548845303257420	0.2624502488745500	0.28034487924942100	-0.23760256565233400	0.2459365026648740	-0.14250411428851200	0.22062050426459100	-2.960242286520260	-0.035221805146263100	93.92289973697430	18.49994756532140	-0.24018185617053100	-10.30289954274670
**ATP_1000_25**	9.611	80.04	0.3823442679463390	0.2588471907393420	0.018451294677263100	0.1215033979141800	-0.0163807421265535	-0.10028460760005000	0.30169181006965600	-3.0874932566871000	0.0759813493263671	94.10075785688430	19.306643516957800	-0.46423594835928	-10.483796687721500
**ATP_1000_26**	11.39	65.55	0.7251407797946670	0.3097693639675480	-0.10840247814328000	-0.6853706101100250	0.11934991383306500	-0.14356251337176600	0.40875595746905200	-2.8244787372535900	0.04495745413617730	95.2936716485412	11.052146849639900	-0.43340741943362700	-8.697428916997250
**ATP_1000_27**	10.99	62.27	0.5346119937966660	-0.09638863482022790	0.5055175462890950	-0.03311446159441130	0.3541486705020400	-0.036726933197638800	0.08895085279321330	-3.046912839122410	-0.06129499782762760	94.08848382801580	29.716268491273700	-0.04496954514531130	-12.42345613693350
**ATP_1000_28**	11.77	94.92	0.38693066283382500	-0.9010585180628550	-0.527718929851664	-0.010070532190394000	0.5281290873430990	0.3461792781166260	0.3983917187669720	-3.0399972621716900	-0.1439952834677190	89.1913349107058	28.018404480485500	0.14734483972259300	-12.199058759925700
**ATP_1000_29**	10.74	58.15	0.6282865954902020	0.33434015532122500	-0.021401782815357500	-0.11312086327020300	-0.04363982006628750	-0.16172051385376600	0.3726018122362350	-3.023768278759680	0.14507437653977500	97.65493041024090	12.03525641263320	-0.8394168419852330	-8.9972355437430800
**ATP_1000_30**	11.05	65.6	0.6491955965887920	0.16282934332870500	0.08990025407493790	-0.2236133304999040	0.09980598972547930	-0.11075277604357100	0.3112383049361260	-2.9695081552067800	0.03373973779644290	94.79799820728730	16.096129744706000	-0.48399850196431000	-9.826520318572070
**ATP_1000_31**	11.12	63.94	0.6773702586562700	0.2658102501055340	0.11479328999032400	-0.19184667295338200	0.16394778976408300	-0.14319033964338900	0.3076898150207990	-2.9972305473419700	0.038887362182781100	96.29254678152070	17.03339352726280	-0.4000434213417770	-10.008301134025100
**ATP_1000_32**	10.75	68.38	0.6123055880607600	0.1783759752875860	0.14767014295227200	-0.18094812179982500	-0.00063483399751609	-0.112341954129335	0.2785595689402510	-2.9996803071172400	0.10770623300826900	96.01233053511930	16.215205902732500	-0.5443718835934970	-9.866304021247060
**ATP_1000_33**	11.57	63.32	0.7145525842533480	0.033185462824518100	0.07507810907818560	-0.14317430176366100	0.01041521922893060	-0.05008209787096940	0.28597121132062400	-2.9992110716516200	0.07647404078215040	95.04662372767530	18.029218401693900	-0.39092719017426200	-10.219669304954500
**ATP_1000_34**	10.75	65.12	0.5775260826184500	0.08885844903466560	0.2865038865346640	0.5603573863886550	-0.007780634105912720	-0.1019579228669310	0.22793127035599000	-3.2493559004252200	0.1097670557694080	96.87225582489400	25.327662627824300	-0.38061335292524500	-11.631347233480700
**ATP_1000_35**	10.22	67.22	0.6032563179233970	0.5588640223706840	0.07640035151133980	-0.10353541840725700	0.1399999950762300	-0.22962700046089600	0.33931333349007300	-3.0243207040731900	0.03599281082944650	95.51646067296290	13.05859705417150	-0.7210328431001830	-9.18170836935234
**ATP_1000_36**	10.16	73.2	0.6376964021437980	0.39953176441550500	0.07824298780105200	-0.32905099891298500	0.028662776773082300	-0.1750422309527520	0.3227650696456870	-2.9616973421681900	0.11332026320216500	96.55347454457230	13.951772543204800	-0.5683476445071230	-9.349226165929330
**ATP_1000_37**	11.18	78.85	0.374127007261	0.1262636298738660	0.2901262532071110	0.6225188223090770	0.037536177438623600	-0.07070450418889090	0.17143825366640400	-3.267043162428550	0.0778346670974632	95.13003787865060	33.8924465078853	-0.08287294211142960	-13.01251519020480
**ATP_1000_38**	9.36	76.43	0.3969993653379320	-0.0392967613467379	-0.2646668203875550	-0.8153802875934850	0.07271732799180770	0.012136981593726700	0.40625011612317100	-2.762811620207360	0.02626694478147110	91.75028509358980	11.449451656981200	-0.36571280193262700	-8.909669216937160
**ATP_1000_39**	11.26	63.27	0.2956700799935780	0.163602239143554	-0.28407590473714700	-0.051101518004718200	0.27150322107240300	-0.021168399260753500	0.38713881880795400	-3.021369766100280	-0.06063787271115880	91.93648039959020	20.60244674605640	-0.13880975958158900	-10.755115856934000
**ATP_1000_40**	12.3	44.84	0.6077899719211640	0.13619589849621400	0.4089463762477020	0.6115737527467720	0.2990295631713980	-0.13022274927067500	0.18290272122738300	-3.2633152234371700	-0.0376299559524957	95.9174135020445	31.189034656340200	-0.18032695402252900	-12.583118862790600
**ATP_1000_41**	12.35	63.13	0.4199609542170680	0.6053608127314070	0.825666461539274	0.8264172952662900	-0.5564776717284960	-0.32672573423250900	0.06838282157230540	-3.3788692034147900	0.43510192908245100	93.0986039475674	32.753104005190000	-0.18751256326256400	-12.853955119477200
**ATP_1000_42**	10.27	69.73	0.634884038541586	0.3524719679725690	0.04484871151845430	-0.4656429265089770	0.12062476881322000	-0.17242135238341000	0.35297755601430300	-2.9125113278526600	0.07592221923310540	96.49803148085030	13.102627720568400	-0.5452096746713170	-9.163919598545130
**ATP_1000_43**	10.72	60.72	0.42064923272561500	0.17547875383140300	0.4163997174567870	0.16486748001838500	-0.042883571931548400	-0.13204961521092400	0.17296928395921300	-3.111900682966720	0.11440803594121000	96.10857872973670	20.243634395412700	-0.4916197510240120	-10.698724118002500
**T24_1**	10.36	85.35	0.4276384736854880	0.15131278032089000	0.00088499644009973	0.47455134847445600	0.1491370490608100	-0.08213923268554000	0.32748212506665300	-3.2077482844606800	0.0138954960245459	95.43658675158200	22.467784365101600	-0.36625529854578000	-11.090012970736500
**T24_2**	13.46	65.17	0.35003138520811900	-0.5079712113269510	-1.010301976865400	-0.14671895051693300	0.21555449999306700	0.24814060150685600	0.6470843064348460	-2.976946474476590	-0.05285230539653450	88.99907601782420	20.35763776188320	0.16947543796812400	-10.788975858586600
**T24_3**	11.52	73.07	0.47843861831541500	0.15632848580731700	0.33320690191093900	0.8847921092599680	-0.27423380972705200	-0.10142697767182600	0.1780981896987470	-3.3447919405345300	0.18901129605969800	94.32787022191950	33.06934779764070	-0.17915836023696200	-12.89053958133740
**T24_4**	10.71	85.69	0.3020583251482440	0.25482696144643600	-0.30291310156960500	0.9206612400552060	-0.35277345095864100	-0.08714140965180190	0.4522892524089830	-3.322204371500090	0.15018687896257100	86.02312544548780	21.27176883202430	-0.48013999338021500	-10.960086288896800
**T24_5**	9.306	63.43	0.6018277372313010	-0.16784628173445300	-0.15677173017484600	-0.8729772009566650	-0.1056537575889380	0.02869780417135340	0.3698199010333780	-2.773739321941400	0.17357625671654300	94.9491918536846	10.324728706159000	-0.5105112002573190	-8.71425219388635
**T24_6**	10.23	70.47	0.4289259859531090	0.40054236836533300	0.33816195008699300	0.6087449265590400	0.05226301252378600	-0.1760414129459410	0.18194366633925300	-3.2267056388650300	-0.009895084531599750	91.21785719886770	26.894586775346700	-0.30219688088988090	-11.872484615320000
**T24_7**	10.37	95.22	0.4527554617588880	-0.5106172758974590	-0.6783845926375640	-0.6726086845008980	-0.10241087750059300	0.18664394332505400	0.556380735584322	-2.8021411403455200	0.09220383355201990	91.00150029072870	11.68532409449750	-0.2686963724829120	-9.020780031394530
**T24_8**	10.87	75.09	0.5759988764374620	-0.33770456242470700	-0.1856446750896950	-0.46177238343400800	0.07749334296398620	0.08874609430160210	0.3651123577946760	-2.892077350408160	0.0552594100824956	94.44536932419130	14.37651112110790	-0.3277029238100820	-9.645723682639160
**T24_9**	11.51	71.06	0.6422290331040920	-0.20596361382396000	-0.1708601885773480	-0.15892577818590900	-0.0716943421182308	0.029098924726180200	0.39138096196928600	-2.9980391016414900	0.13746877990995700	96.24429010249440	13.42915057547680	-0.5573741637879990	-9.411951629153910
**T24_10**	11.33	70.41	0.5488786092320250	0.051245796770284100	0.09617076060539580	0.505785050918597	0.016471716359427300	-0.05250557552040430	0.26963998912754800	-3.1946719233543700	0.026709697665035600	93.56164127980170	22.339565436105100	-0.38532900340344430	-11.088971708372600
**T24_11**	9.093	67.1	0.44035183790727400	-0.46879972526592500	-0.7904682110765370	-1.0182510884623600	0.25259814502972700	0.1781675481512220	0.6135829321477680	-2.709636517978470	-0.02337055208300200	92.16673297320660	10.733624364719200	-0.34511612631947300	-8.805272059695720
**T24_12**	10.6	68.12	0.40742490160075900	-0.5694516089113600	-0.2326271742303290	-0.6831892869968310	-0.16674995440587900	0.17748155546801800	0.3568780344085370	-2.8334963494485900	0.17798439022221960	93.15945597789220	15.522069132052000	-0.22527580343018700	-9.88408452340343
**T24_13**	11.48	67.95	0.42482876459116500	-0.07175797224273910	-0.4149433918684230	0.3630561796745270	0.15482951797220500	0.031003475494400300	0.4646258444260940	-3.147989388189730	-0.03024005263212450	92.60562924731760	16.606242781606200	-0.5575076332695850	-9.990946321261760
**T24_14**	13.56	60.15	0.6491935957681510	-0.011619783184991400	0.12297120720278500	0.10197071385970000	-0.056700063796344000	-0.07248166991400210	0.2987343024448990	-3.087488265781100	0.13896172266936700	97.18983222363470	15.503672497409800	-0.46564888841323500	-9.779893963400340
**T24_15**	8.907	81.48	0.5157154092769140	-0.20170874685750100	-0.552959546577008	-0.7880552628289220	0.27529236260918600	0.0769597666744899	0.5218316461755310	-2.7738740629602900	-0.05332219787493150	92.40068439072140	11.329870808565400	-0.35147982692855500	-8.922969895911910
**T24_16**	12.4	59.06	0.5008053349267650	-0.2895764567924120	0.34724338396090700	0.5953602694961270	-0.11894381031863400	0.0114312700130636	0.17694984938765600	-3.25068266719831	0.15759723489867200	97.19247283991090	23.873478418058200	-0.38199594876828600	-11.434034892981600
**T24_17**	8.662	50.67	0.4344320898610340	0.234300815587312	-0.056137048358037800	-0.8510993863060010	-0.04637837102003350	-0.11046622753752000	0.36143343911306200	-2.7693240897135100	0.11990943603667900	94.24230082736450	9.262568577083560	-0.6834498909564290	-8.35053216213643
**T24_18**	10.54	59.08	0.6899841703776160	0.32696702484098800	-0.18312467790041700	-0.640715074104944	0.22585166032299400	-0.14641132229211000	0.43990157628760400	-2.8318748554879100	-0.005790758423259710	94.8089687428585	7.773807646815450	-0.7677508995211750	-8.043342305763360
**T24_19**	10.61	74.04	0.6312298919950970	-0.13009692650044700	-0.18629260795852700	-0.3087522397678030	0.07612109875576140	-0.00015468378429411500	0.4107836998312280	-2.9478689308106500	0.06674614481530870	95.83806790275830	12.180038823868900	-0.5776038825577870	-9.155103410299130
**T26_1**	8.6	87.23	0.4326941574834310	0.35261727090088200	-0.1319910002115380	-0.6893276364296780	-0.01972257156812150	-0.14165495139238300	0.4026637184411110	-2.8171798905738100	0.09207785876913190	94.63471809411160	10.138742917168500	-0.6606632610768620	-8.464865494054890
**T26_2**	10.39	62.15	0.5041163799164080	-0.11177173155441200	-0.06384907983472440	-0.4545693001112930	-0.3709188478376710	0.00176176146665560	0.3382987817713950	-2.8950986764333000	0.24645026565356500	94.6030609475934	10.909813568801800	-0.7699924682076010	-8.846145275138110
**T26_3**	8.541	66.06	0.49793869809422800	0.026604452112669400	-0.20395923665858500	-0.9510723853684130	-0.12196116026662900	-0.03959894496954460	0.4172494807931090	-2.743682156647920	0.17152305805308800	94.69845816687080	7.908466740726230	-0.7017488319217080	-8.098837234356040
**T26_4**	11.01	76.35	0.546957881895611	-0.3540531345346770	-0.03770796545761470	-0.28409932880509500	-0.3021428084939850	0.08486077831954360	0.2994424495078570	-2.957813494763730	0.22620103767131400	94.53465541408690	14.398487803541700	-0.5220705633545960	-9.634724844315570
**T26_5**	9.673	110.1	0.32409781198362800	-0.8463680966365610	-0.722340825192133	-0.8178943888608490	0.2979329092085400	0.2968115232715700	0.548018920616818	-2.7748231311777300	-0.03821582880023820	90.87872801298900	13.98807641915440	-0.1501643627576810	-9.554569948999750
**T26_6**	8.772	99.83	0.3989183208549550	0.41876786663207800	-0.28144222716467900	-0.3192262881514880	0.09433879434398810	-0.14160086736080300	0.45310309961233100	-2.934439703602140	0.03101144988071130	94.56737487262370	10.60874870647740	-0.8059178979396040	-8.571571782320930
**T26_7**	9.781	70.21	0.4285971572126370	-0.040898258940600500	-0.20207528668525100	-0.5661884089117810	-0.30548183733812200	-0.020130204864175000	0.421161085184885	-2.8653030346822200	0.23891848393110500	95.34013596088500	9.179852525911870	-0.8243694139055790	-8.43741623398409
**T26_8**	10.42	69.24	0.4784511791112230	-0.5547564679306770	-0.8337035144469060	-0.912834751301297	0.020599824136626600	0.19644399477955200	0.6374159892384360	-2.740721454003220	0.08754363039648150	92.70404097870510	9.84934719916379	-0.3397714061515770	-8.58520412795542
**T26_9**	9.875	70.26	0.46695831174037300	-0.19192514411752100	-0.29405604376466600	-0.5713974898874990	-0.04320169874430390	0.04550390217666680	0.4292063998711220	-2.8614336722135200	0.11996933213096500	95.07764864779740	11.497512638426700	-0.4882750810229840	-8.989144422578980
**T26_10**	11.1	77.27	0.4657640511025920	-0.07226427885421170	-0.30155687392477800	-0.29144922007322600	0.19469707522000800	0.026580036905741900	0.4146836518340340	-2.9285354774745000	-0.03659050153615560	92.56576844739030	17.335697509755000	-0.17057267816774900	-10.110718485004900
**T26_11**	11.23	63.61	0.4664289437196340	-0.07592246212959440	0.13094266310767600	0.1138097245006530	0.20252812980454100	-0.02089144924074470	0.2500388640718810	-3.0896812039071600	0.00364561496592114	95.6215756158869	23.457466031121400	-0.22615436147483000	-11.261557790424400
**T26_12**	11.85	65.17	0.6408616593663070	-0.15268856887899300	-0.05974462467216620	-0.08945328936432630	0.016446867485256000	-0.002829663252730770	0.3559078441629320	-3.007661518974110	0.06754692900322660	95.31181141283100	14.167733038835800	-0.5531017264929680	-9.56371768999402
**T28_1**	8.785	124	0.40147539380969900	-0.6674891055892960	-0.172682472307633	-0.4449009344720340	0.17473841714550600	0.19278434237123700	0.33571969845243400	-2.899095568171560	0.015324731471097400	93.43212957998140	18.277647332553200	-0.22523567852233900	-10.458235087571800
**T28_2**	10.26	103.3	0.36582288310411000	0.10178046148635100	0.21745690145152600	0.5794263701131480	0.16742493185498800	-0.05798260442256840	0.1979474408405080	-3.24295520124308	-0.0056610891783401300	93.6991638971321	33.268089523556700	-0.08520392695186170	-12.918372939967900
**T28_3**	10.76	77.05	0.6005189620277730	-0.14528808337636100	-0.3150349487848640	-0.41895449241928700	0.030429369842261400	0.012699206002017000	0.4652427113107950	-2.908100619156520	0.08794137688851430	95.81217591115260	10.496543795945300	-0.5945996242116590	-8.762594723271090
**T28_4**	11.11	90.12	0.2848005683852890	0.2642625556041910	-0.6013723255937170	-0.07646969084462920	0.03861021036311000	-0.05871032081624440	0.5596379953649110	-2.9855900371858300	-0.009140610379615170	91.45353356799340	9.539199240627220	-0.7685587019808990	-8.295189470906570
**T28_5**	10.18	81.55	0.48718983700976800	0.07882136439443250	-0.1823614978732180	-0.2359983288766840	0.05986642334720650	-0.06447806049176790	0.42169126666564600	-2.9746877693220500	0.08195520804724720	96.59798919914520	10.755134710712500	-0.7400540664798770	-8.803230321429210
**T28_6**	11.47	81.04	0.5693670724781670	-0.15822277962294900	-0.15453049186498900	0.04590844637426220	0.013304431696568900	0.010823558742161100	0.3861162135915620	-3.063611718630310	0.09046470817854200	96.19769722152550	14.447008947062700	-0.5557862855164490	-9.60680838361006
**T28_7**	10.84	59.82	0.5990152365878280	-0.25303137512065200	-0.2364392504662940	-0.4608693091501290	-0.011917061408396000	0.04487197724572900	0.42091200873314100	-2.891995036791590	0.09818880200980370	95.10952572592300	11.98497188124980	-0.5394304835328510	-9.122489055285750
**T28_8**	11.77	76.8	0.4596515180509990	-0.33337244273601800	-0.030430486796117900	0.48806535756196400	-0.03378900102692520	0.07127253401697990	0.3061112099183260	-3.2157886526926000	0.11391308433789800	96.26983187304180	23.94571500701410	-0.31212716926301900	-11.385843143155600
**T28_9**	13.27	70.8	0.33253759192802600	-0.4148581728063460	0.22169834919912200	1.2665732385534300	-0.05269569542069650	0.08002440494829170	0.19747018110181400	-3.4629564235720800	0.09700847825396990	95.09470729204280	37.55828758669030	-0.042623188279482100	-13.586258153894300
**T28_10**	12.24	74.37	0.36072386336900400	-0.185927417118709	0.16363717433317200	0.7732813634619540	-0.09606115861112390	0.021053044100500500	0.2206405872798430	-3.2970009799705900	0.12143273179768300	94.70424439832410	28.935010630889800	-0.2003715306066100	-12.237373558585700
**T28_11**	13.65	82.48	0.5265368237847210	-0.7362536198456770	-0.3564985151528850	0.14540766192810700	0.25346320323928700	0.23497606281128900	0.39452394951757900	-3.087613241090750	-0.027797045264873800	93.81519066396210	25.101473113859800	0.22121620847864600	-11.690192295029100
**T28_12**	10.64	94.01	0.3377264279372080	0.5141798881477260	-0.35614625838339000	-0.32541869504608700	-0.12053261662951100	-0.18689180791359900	0.5184642013125640	-2.9369261739870300	0.13667427586418600	96.09865534897610	8.539058866968470	-0.9046722796553410	-8.045653283372910
**T28_13**	11.7	70.25	0.37283672388677000	0.16352453553544500	0.5113764042952560	0.876770298797598	-0.22862515420481500	-0.1073118076540650	0.0874689050933941	-3.3202269563779900	0.11171486386052500	90.99690667956640	35.79852612350140	-0.10930786618250900	-13.30407338014160
**T30_1**	10.93	85.64	0.6373977386645530	0.019865006387842300	-0.4876816137983700	-0.3787036516417820	0.0295133514498935	-0.0273993297009269	0.5486206908484770	-2.923065486959530	0.09524220603078160	96.18412125067630	8.62947937513898	-0.7117937443044480	-8.280973318191530
**T30_2**	12.06	67.55	0.6083396150164370	0.10990191627100600	-0.19428866530778800	-0.2068424583992040	-0.13375724185322800	-0.07010041665272240	0.42124798401716200	-2.9741813484706000	0.14990402686127400	96.37816337802160	10.291163086840300	-0.741158225471755	-8.644155405549050
**T30_3**	7.715	81.11	0.5320819394771240	0.3246999300535020	-0.5002088974476680	-1.2688636998136800	-0.0976593456668943	-0.11850517937319300	0.5744845612502480	-2.652290907936270	0.18970570235145000	95.76418726162750	5.02176620066801	-0.838193922736264	-7.193374277768150
**T30_4**	8.225	86.17	0.5326479804122460	0.05678111615529130	-0.3535037868993130	-1.1033427902442100	-0.13792519254566500	-0.03183109904103720	0.4762275462745430	-2.6985433872097500	0.18913122575649500	94.90401567432990	6.917255080671160	-0.6620461616280590	-7.791145319923210
**T30_5**	11.68	81.88	0.6334635201839760	0.07247479343013030	-0.08393822379391830	-0.16405583768521400	0.03099302157560860	-0.0769308169384931	0.38127572635273400	-2.9785011713530700	0.06616521873536030	95.42799426113780	11.507181636798900	-0.6341352301053400	-8.944919195578560
**T30_6**	10.53	111.9	0.440856924350929	-0.9413355287158590	-0.7107651607510980	-0.7577891312879080	0.3130336621755890	0.3211389548411760	0.5424282624686650	-2.7947291864066300	-0.03953781800208530	90.9508537002335	14.730739020718000	-0.05443393447403440	-9.712906371277550
**T30_7**	11.57	72.67	0.5372550189601970	0.2380300492104130	0.05167020849377800	-0.21518143256613800	-0.5853458869868470	-0.12365896227285700	0.32514899563565300	-2.9734236837293300	0.3303199209975870	95.45815098938730	10.849688788261300	-0.8282365025577740	-8.72909637997797
**T30_8**	11.04	86.12	0.5172746190889930	-0.05117247245641940	-0.09805297692128570	0.1666648255272430	-0.20597604233689500	-0.03201029608784340	0.3768156755821100	-3.1033043695490000	0.18329471359969300	96.5219585826697	13.845829269123700	-0.6784068470587110	-9.434880588839570

T30_9	11.26	114.1	0.3654113182219310	-0.20482188669109500	-0.2921777497642000	0.09910746839662030	-0.10261944241748200	0.05854468787333150	0.40761089760829800	-3.0598243362445000	0.0883026811072294	93.24788617172170	13.892806298947700	-0.5393753626137140	-9.448110993421140
T30_10	9.422	83.18	0.4754981200831690	0.0656655380858219	-0.22607646313460900	-0.5432204703789840	0.012314429592802000	-0.0397286861302205	0.41471965633259200	-2.851284941827580	0.05531854952163190	93.51464216466550	11.132995801284900	-0.5871330363518820	-8.791330232019240
T30_11	13.28	66.89	0.5295045148128650	-0.2811199719705590	0.024516984894530200	0.21407780691925400	-0.041303601558317300	0.03991029970082870	0.3007855085634570	-3.1051912662484200	0.08370003192033130	95.38496741486210	17.609990449344100	-0.3576063583062770	-10.241534989300300
T30_12	10.71	105.8	0.44523924917495500	-0.7677798942804420	-0.5362278836183490	-0.3847399112959390	-0.01433075013398290	0.2438402988838680	0.4818398424501160	-2.9074225345643400	0.08898293795797670	92.49010684399080	15.104277512632400	-0.2155352333779640	-9.805973728646110
T30_13	9.741	87.82	0.4053938844843490	-0.23264172385902100	0.0276213806390576	-0.30896719600978100	-0.41411368106553500	0.03455249559153410	0.2918452206536420	-2.95300159353073	0.2867362592856470	95.00997514808930	13.192219149103900	-0.6719668066030190	-9.366223072691560
T30_14	9.004	104	0.3618470476281580	-0.2363789819648090	-0.6195713451173680	-0.7656378336243650	0.1802494571606700	0.09190965358246690	0.5561604363918870	-2.7945742548226000	0.017432314705589000	94.04897716178920	10.982406484547100	-0.3196738621243250	-8.839546089761010
T32_1	11.61	96.22	0.39059878315937600	-0.18108516800926900	-0.26425176309242500	-0.8312033175948920	-0.23874087998516400	0.08239552314177450	0.34904931837258000	-2.7430922914198600	0.17499658101144600	91.07502689117690	8.391439465278760	-0.36198382064964600	-8.180706638656680
T32_2	8.479	69.03	0.40636097176419500	-0.015757739214973700	0.031163741857919000	-0.5297717840629160	-0.3957345543546810	-0.024330043512969100	0.2855554814667280	-2.8549785325627100	0.2509319748442190	92.56383124958940	9.39378375507811	-0.9180989232271210	-8.487367303406390
T32_3	11.31	75.54	0.6108988350353400	-0.3679732972168080	-0.11275930111450000	-0.5807484326933610	-0.14033577902052600	0.08275934429588670	0.34847814295486600	-2.8504058238104800	0.16621802551143800	94.90222595555880	11.93807278873260	-0.4174804884671660	-9.106766300543600
T32_4	10.46	81.72	0.4574090399568690	0.04483791713312480	0.24349515566859600	-0.036160524862171500	-0.2560565880972000	-0.08734782246527180	0.2298808360846240	-3.0340586267272100	0.18859445210401700	95.73195843213290	15.03969357107320	-0.6896352533694450	-9.70608695881348
T32_5	10.01	74.58	0.4547913962129930	-0.004292116226421310	0.005646137772368740	-0.7616302810165170	-0.364689728644131	-0.02905950841585510	0.29575987249205800	-2.7795395525409600	0.23825961805136500	92.58584301528970	8.441212591107260	-0.7479304507259140	-8.235968256017700
T32_6	11.21	84	0.5088937618305710	-0.43919782958882000	-0.044284013126355200	-0.10499757188264600	-0.25905778374932100	0.09815905320178210	0.3112228224569130	-3.01118255950812	0.22432272710716200	95.86121205626760	15.578627022969400	-0.46912593842594400	-9.876113645802730
T32_7	13.03	71.24	0.5471199031070460	-0.03388890470989010	0.03605523858940420	0.6410831507778350	0.05596009753547240	-0.044105417466371400	0.2888935412524760	-3.233883199787070	0.019423406478202400	94.4973196791532	23.69766576117250	-0.20056209150506100	-11.319659315894800
T32_8	8.826	78.01	0.5841651392299290	0.1632610317869880	-0.5111179795829330	-1.0620001453569600	0.06826324835143750	-0.07354023317723260	0.5690588345107130	-2.6955164029447200	0.08825835016317480	95.05732667253450	5.888372753155610	-0.7388737678413480	-7.495032847667290
T32_9	9.058	81.96	0.5393717713482950	-0.07687358000023890	-0.163639273027202	-0.7467417113566850	0.09744289701079250	-0.019069989501291300	0.39005926584579900	-2.789286440455350	0.05406368158015730	94.44606836192050	10.075170584045300	-0.5681623645790930	-8.664007878382710
T32_10	10.62	66.03	0.3395477104453380	0.15284377506006100	-0.08209000865600110	-0.17044831180553300	-0.35777796860646500	-0.06490410868561470	0.338950807389221	-2.969786886692590	0.2239990643462080	94.38637769262070	11.12410258781590	-0.8941921588087670	-8.792461703404940
T32_11	12.17	79.16	0.4802295228066490	-0.44283624815682300	-0.5538622135246550	0.4534147764131360	0.04243803705748080	0.1850560954132810	0.4694933830289850	-3.172595002424820	-0.0024565386589372300	88.03821121496500	19.717079255643800	-0.4038674063514420	-10.648359401223300
T32_12	10.95	76.42	0.36375012057728000	-0.4561297565560750	-0.8098064245491900	-0.4858526583614440	0.18769196698279400	0.17628337102527700	0.6137298756342790	-2.860406588125770	-0.03328455625481240	90.71450858019400	11.233258467455800	-0.3846260459535900	-8.935947400893000
T32_13	9.665	112.8	0.33417522021228000	0.003442585733084820	-0.6363843089214810	-0.6177545232083390	0.09502831632519370	0.03931775405604330	0.5474882176051490	-2.83620881962642	0.0351678262215091	93.13840052283010	10.850660843895700	-0.3608066851445200	-8.688898373335990
T32_14	9.921	106.5	0.32307656423555600	-0.3100653714862440	-0.5247013694309500	-0.4492893162808900	0.030293860139285300	0.1396432058578920	0.46622105296617000	-2.867179338324930	-0.000669520108221508	89.4981006238543	17.313531930466700	-0.11348029224819300	-10.181704456828200
T32_15	11.9	69.57	0.7005679999113330	0.18280419379246100	-0.3209220939085330	-0.605244752903742	0.10809098115185200	-0.08529559430923130	0.4813685278342860	-2.842250669304120	0.058028150832581200	95.59939421145230	7.328047629268210	-0.6705873855038420	-7.931017160091860
T34_1	9.724	103	0.4653001739063790	-0.15431637807964000	-0.25743159226508800	-0.4475801557039940	-0.10069831740314300	0.03339875609566050	0.3985117765943370	-2.877156736780560	0.11458182068994600	93.73509704783270	10.456167928763800	-0.6371220371102200	-8.747679927989400
T34_2	11.55	75.4	0.3816590381267140	-0.09964405111616550	-0.08638198617351430	-0.29214836339000400	-0.20257247772622600	-0.009435471227639130	0.34931279960272300	-2.942491817478160	0.1888253962693460	96.03736807223800	11.313746171464500	-0.6362972582707540	-8.921381668593480
T34_3	9.555	92.31	0.43707448764080600	-0.2946657202520140	-0.19624749235940800	-0.3838310723516770	0.0007498504269160470	0.07132767577429080	0.37013340585162900	-2.9118642808270800	0.09355640482180810	94.6617563460184	14.415693789572300	-0.40474259466387400	-9.643512170746090
T34_4	11.72	89.68	0.5036876351106520	-0.12653845302820300	-0.070322113896373	0.003723449944795140	-0.051951003655642500	-0.009165534851909080	0.35903009565725100	-3.038446565225540	0.11802835009998300	96.6338313664814	13.796788381995100	-0.5263610884813710	-9.454923919329820
T34_5	11.14	95.55	0.5911953197259070	-0.18089248067227800	-0.21656771201859700	-0.5883995572844570	0.04255367046025660	0.037783344821333700	0.3943995194054760	-2.8256120397152100	0.05696545018374420	94.0966986988929	10.613711318366500	-0.3608397904127510	-8.774260264660950
T34_6	13.54	76.57	0.5017460609104300	-0.0476175257695324	0.2209247838033840	0.6287035278575750	0.21886780483076600	-0.04435091863713480	0.22448481313122900	-3.2472432207281500	0.0005336028182914570	96.48665173378570	27.21767694511970	-0.01794931818645000	-11.91067483148930
T34_7	10.36	129.8	0.36236097324649400	-0.35253890255520600	-0.46551099668395400	-0.9197966358095470	0.27783425757373100	0.12005877721538000	0.4758450205237160	-2.7324665891521600	-0.04438777389544600	92.36926799844100	11.497909909978900	-0.10113451464837200	-8.962919965101590
T34_8	10.36	96.89	0.3454300322757160	-0.09852361472301820	-0.010896727584547100	0.21334594440959500	-0.25773346218473400	0.025213968718830500	0.27159483729513100	-3.1034730275369200	0.15257297423491200	91.31939347137710	18.0296918689914	-0.5631355568838950	-10.327714473405700
T34_9	12.85	126	0.39240658957888600	-0.0517154271860489	-0.19539967045956800	0.4475875514826010	-0.12938609872378300	-0.002512483597616940	0.38172600366971500	-3.1585205005484600	0.06862230710146470	92.96583535116470	15.339588739498200	-0.19778442017191800	-9.713331716445950
T34_10	12.7	87.38	0.5549722760084720	-0.04960360739397160	0.001560079032232440	0.2726202535648800	-0.06945063893953690	-0.03777055478130380	0.3374431911147930	-3.1485128263242700	0.14043548579892600	97.2061121159487	16.537975866926800	-0.3864069903644290	-9.993586150680970
T34_11	13.17	87.16	0.40589730868173300	-0.5082202354582300	-0.6531249257983390	0.2572862240643090	-0.007637243801460800	0.20428005647723000	0.5094503515835840	-3.098811954226220	0.018358732814500700	89.37087542147210	16.502010265920000	-0.2652620564830300	-10.011231782621200
T34_12	10.44	92.39	0.4621724765731150	-0.019191859571637400	0.08787510510516340	0.06171198369199940	-0.08314891550355980	-0.03265595677324610	0.2686511761882750	-3.0574991264061300	0.09624579874866710	94.49721980168520	17.13665958912100	-0.5263033756027460	-10.078752145428000
T34_13	10.8	88.38	0.4213622266737770	-0.41603144277543400	0.1538174138366600	-0.6225981702205830	-0.635121385094753	0.10141097840801600	0.20621563870186900	-2.840590960291080	0.35683845702261400	91.2362367822109	11.752179153353300	-0.5312759495534990	-9.054890162036820
T34_14	11.53	74.45	0.5940918790025080	-0.17924831458219000	-0.40859737644404200	-0.6478192069283680	-0.08733638687205280	0.06082625528831100	0.4671649384314490	-2.8305415470896300	0.13386912095644900	94.6130743337895	9.150788520258780	-0.47898933253124000	-8.416332501225900
T36_1	12.17	119.6	0.387078027146739	0.028361313406831400	-0.8530892094110900	0.07139895278726830	-0.06053908833019730	0.05161613215801640	0.6209585681383150	-3.0084046410965900	0.0021184857783870800	88.7756316874807	8.919158538104700	-0.6474670806138660	-8.166475201680910
T36_2	8.852	99.66	0.36676899803307800	0.12242641689012600	0.318962032285673	0.38397282429300100	-0.1384867298827060	-0.08944738303517810	0.18083829100763200	-3.1595745635905600	0.1253468501119110	94.41379330749860	19.158097951306800	-0.6204687469307670	-10.556519027469100
T36_3	10.54	102.8	0.44781696263433000	-0.008033722290066360	-0.10747942313445800	-0.6629920671942000	-0.33351406246035300	-0.012426328905062300	0.34037873676401700	-2.816580322521370	0.23460511988240600	93.51373322077080	8.68024606623237	-0.6558498595260140	-8.284105184666300
T36_4	8.702	94.82	0.4141625406692940	0.3996788898911860	-0.06798623211319590	-0.611448160767506	-0.26238658786306400	-0.16113502499440600	0.3812573475627630	-2.8593297791178900	0.2508107978936910	96.74011946596420	7.304705561301620	-1.0288852326069600	-7.909220151298580
T36_5	9.408	115.2	0.3083199317751780	0.30828822662182100	-0.2669101688060100	-0.13593208639084900	0.1978890625691180	-0.06256288203867850	0.38444951461709500	-2.9816824405188100	-0.048375376991136900	89.82142639588900	13.818898649910500	-0.4476711474019730	-9.30682472876721
T36_6	11.36	103.7	0.4599892141490600	-0.15202672574203500	0.03237238188895140	0.4825464016068530	-0.06331285995160470	0.020441778340739800	0.2723044174019370	-3.1825819743746700	0.05150592998396510	92.69911280839350	23.647334520382900	-0.24086799147845400	-11.305706624639400
T36_7	11.15	141.9	0.3224807127595290	-0.1284411739637070	-0.377090910346052	-0.2889512300161920	-0.19805977975243000	0.04683476827236360	0.4513524636726580	-2.9493970667639600	0.17684352625122700	95.16678754384970	9.840293845524010	-0.4572365148853150	-8.54955744398037
T36_8	10.42	92.92	0.42717953195998100	-0.06517397006832520	-0.20415168704969300	-0.28909015234 18770	-0.15995345568931400	0.010226152188212500	0.3744409099708580	-2.9392627038305100	0.14145013444560300	93.79832088314960	10.735681102521400	-0.7180468485015870	-8.784623118543690
T38_1	9.769	151.8	0.29030051742075400	-0.45584654368611600	-0.7710570666586580	-0.8093277717988530	0.15051039188686700	0.17589407454422100	0.5864847067731260	-2.758315996727160	0.016464360616015500	92.25717246678040	9.653744133011960	-0.20453500359682400	-8.513031920004480
T38_2	10.89	137.7	0.384818272422356	-0.11047558961166100	-0.3705444693139440	1.0418413266804800	0.581238074508938	0.03825985254130970	0.4545901078846420	-3.436335803105180	-0.07932777688179970	94.95868088935310	37.96340858832370	0.10602845466764800	-13.655409375461300
T38_3	13.76	81.92	0.4696237854923550	0.11214982123547300	0.11547425687945800	1.0257546188021300	-0.2759603832257560	-0.08193636012134510	0.275472070541598	-3.3727412835489500	0.1776751199327470	94.06500161812000	27.299574230917500	-0.01895926156524520	-11.952452076860000
T38_4	12.05	78.24	0.4178574640388070	-0.14557578438536700	0.331508529978893	0.5678022249930630	-0.36741319657431700	-0.015166460143687700	0.1670560936866950	-3.2273092376704300	0.22438782808088600	94.48961947456960	25.955346608567100	-0.24628203545581700	-11.733702963516900
T38_5	9.991	114.2	0.42026024245808400	0.04969354134666480	-0.7354000224540620	-0.9299274532096740	0.06418077931342680	0.02877784548844340	0.5862808128058940	-2.740090434043630	0.09159732777211620	94.25483100678430	6.51723582918996	-0.4823682001972060	-7.616143033603720
T38_6	12.86	92.96	0.493421686471447	0.29133117107187800	0.13460780746334400	0.3307870344893690	-0.16977586982856900	-0.1698571475495580	0.3011384342030300	-3.162649766187630	0.1834509674046010	97.16512169212920	13.565062880734300	-0.4369286357898090	-9.338844961766140
T38_7	10.91	139.4	0.36837636109434900	0.198928499290231	-0.5824489010505080	-0.44396442375192900	-0.035250192107098100	-0.04849474142323780	0.5593986017480300	-2.881954138097100	0.08046964026033960	94.42343064630790	6.091390033845700	-0.7527783310469890	-7.548873136438210
T38_8	10.84	124.6	0.5221663929878550	0.04714916978698520	-0.261508956469105	-0.2022452461319000	-0.048539068952284700	-0.029878706813700300	0.4152437538724010	-2.9690412526793800	0.09627809343818140	95.00528024333090	11.715388366258000	-0.5629427312547400	-8.916382582109930
T38_9	10.33	116.7	0.41019869966696400	0.07950134836442880	0.03220777070450170	-0.06589959522362150	-0.1006815559532850	-0.06791832860662710	0.3058346706104030	-3.045689775096030	0.18063744460753800	97.3503821967171	16.04773022788650	-0.48950493637031200	-9.829667772427320
T38_10	11.14	140.4	0.3492485120716800	0.04914944427796900	-0.2632031613979560	-0.4285956884814660	0.17885993965709100	-0.014655384352589800	0.39133932607059100	-2.885794155172630	-0.02778793947461940	92.68767697804350	14.601054101410000	-0.027279718778126200	-9.499555047933170
T38_11	11.84	91.21	0.5089195986042970	0.018243754956025400	0.040657294736189100	-0.20625446697519100	-0.3417292620737390	-0.045315233564265800	0.29599471363332900	-2.9820710079173400	0.24763804713914500	95.77671469169270	12.004000611725800	-0.6336664233265310	-9.014400144053660
T38_12	11.39	87.34	0.369433962677426	0.19900518853019900	-0.014601107586209700	0.5120529348307920	-0.2110985771309590	-0.09496103415924850	0.33269785813656900	-3.2127418606342800	0.1588166851599570	94.99601909796520	17.443436022830000	-0.5131685474954200	-10.199979165932700
T38_13	11.69	87.41	0.4340268475941830	0.4918738610688990	-0.29432498252356000	-0.0567554325571884	-0.31547735842434700	-0.1720185267079830	0.46537432274230600	-3.0211798621486200	0.21146587484850200	95.3213739792887	7.93486430926158	-0.9779992575499040	-7.953564842104870
T38_14	9.811	74.85	0.38992177676621100	0.6638968544836850	-0.000267111341263543	-0.5488874765430810	-0.7031244335089080	-0.22719663319711500	0.32946653822677900	-2.8743544036959300	0.41021643645468900	94.79589066022800	6.955021615591020	-1.2950843303436500	-7.701579492556440
ODA1_T22_1	11.88	20.65	0.4338242841806640	0.1349250522630680	0.8777239658682190	1.3634518484417200	-0.4794066825351800	-0.15028347495032600	-0.027267721144793900	-3.515052038801170	0.300127828396586	93.89022668349110	45.694713744650200	-0.21846364600822300	-14.750275924151800
ODA1_T22_2	10.82	26.5	0.44474048246383500	0.055128356140990500	0.5597869787430930	1.1255277656180300	-0.2805185184258680	-0.09491661203650150	0.08713607063125230	-3.4296217881421200	0.1919644798987940	94.41422590266970	39.90417356972270	-0.2562499119586870	-13.933161145081100
ODA1_T22_3	12.84	27.83	0.42065281248683100	-0.2806512201137700	0.6388878992420640	1.258926519931980	-0.09714083976315310	-0.0004168024578078860	0.029542430818039000	-3.4687542935199200	0.12585201509125000	95.08189891678370	29.765782752655600	-0.4034163124542440	-12.457742031916300
ODA1_T22_4	12.88	25.33	0.35421827107759200	-0.11891962839896300	0.6542924152646030	1.2182338841577300	0.13371152681156300	-0.05953685305036660	0.045084379764463800	-3.4517367529375300	0.013971180785402300	95.38933611323250	41.00917852673590	-0.18712810713246700	-14.087559833786300
ODA1_T22_5	12.29	35.35	0.3316978284725290	0.10098678843287000	0.6064900754055070	1.9295438018019300	0.4208643265266780	-0.06077492824080310	0.0011575568374615500	-3.722430833982190	-0.08264154577738280	85.22954566937230	29.823930082464800	-0.3946230739026180	-12.466429129137400
ODA1_T22_6	10.58	22.15	0.32995232912543800	0.2917004000927990	0.5781828034665880	1.0051874258531700	-0.31917399967681700	-0.1728265625481960	0.10111113363680300	-3.3995074333800700	0.23289074582929100	94.73660710120180	37.40767541662340	-0.2809468745925690	-13.56406270662850
ODA1_T22_7	12.56	18.24	0.48243157788057700	0.2324819152371810	0.7837163677429350	0.8687912060387700	0.34155398171364000	-0.18198360757795200	0.026412832942986900	-3.344194320796460	-0.0636504166143105	95.36751221417390	36.12158792944620	-0.21609986200525400	-13.364387609515800
ODA1_T22_8	13.59	20.74	0.49705382220260900	0.3188766324131620	0.5890039576324030	1.5524963732674200	-0.14638094351489600	-0.19962407663444900	0.11644782894116500	-3.56001392692175	0.12943542339558300	92.99677441032920	43.82501955486230	-0.1620328773338700	-14.483278864314100
ODA1_T24_1	13.59	18.98	0.45453342054000200	-0.0717394580432093	0.8207567009659290	2.331923142473600	0.16416356352148800	-0.05228636273742430	-0.05651746643233840	-3.836653336793970	0.03431049740571430	89.46795271834460	30.049202832984800	-0.4172520298778870	-12.506388958222700
ODA1_T24_2	10.18	41.89	0.358296434003974	0.1125475348242310	0.8351440054852860	1.5278147306402300	0.25095014864731800	-0.11319106266159200	-0.04790285951970020	-3.604228968123040	0.0540540976960438	97.54682492861850	55.3463288031687	-0.07214427348714300	-16.033568793618800
ODA1_T24_3	10.14	26.36	0.4674008770054650	0.16477045109102900	0.33203573862575200	0.6081833783428990	-0.1655219276353280	-0.11080715046732900	0.17807398599957600	-3.2624523663605600	0.15071080993158300	94.98479852462530	26.60596701968790	-0.44594367229099000	-11.854904835542400
ODA1_T24_4	11.65	43.9	0.37660873389429800	-0.3905557456614940	0.25369687839731600	0.10532239185633000	0.177965366555612	0.07875387440078320	0.16320465423462300	-3.114897038228800	0.04964224769631040	96.63484495994150	31.39396418123520	-0.058277880089358200	-12.718273110633600
ODA1_T24_5	13.74	36.11	0.4523452011855310	-0.46169475136815000	0.2500803774186470	0.8144142745974880	0.5261396500952530	0.1271182016048340	0.13345121337624	-3.3207647001845700	-0.1266232029464440	94.32948556360370	40.62945228251970	0.06891612463836370	-14.125017399754800
ODA1_T24_6	11.45	36.61	0.3792421539873710	-0.709926413682904	0.4040015443291340	0.2053772905362170	1.1270486384896100	0.2101514451014890	0.029294762878971000	-3.161983547511540	-0.3126931534111540	92.39918317315630	36.11743800782910	-0.0562808556797273	-13.491212141765600
ODA1_T24_7	7.436	30.46	0.36853749314879800	0.4301860043075800	0.2464528012773240	0.22338473903592100	-0.18836381224236800	-0.1747057883067070	0.2265345611806370	-3.125700935994710	0.1376902108224110	93.41647106521560	15.48894230347790	-0.8205169115605090	-9.795447366811470
ODA1_T24_8	12.24	43.81	0.32182594378953700	-0.12355905046253400	0.9010823463517500	2.282844798466880	0.7947645316749470	-0.01054287421825790	-0.14723421981010200	-3.8464156792230800	-0.21291973294050600	84.30275415662910	29.778939889845500	-0.3665492490903520	-12.456540152234400
ODA1_T24_9	9.942	39.2	0.4968415052359920	0.42459299529793300	0.44659692751170000	0.15731389759409200	1.114912252257380	-0.19887039884775700	0.15036057467600400	-3.130537688922930	-0.33985244894458000	94.30517148659120	29.78095685882030	-0.0910213521064767	-12.48036558973990
ODA1_T24_1	9.852	20.05	0.41546492766616400	0.39783766216216600	0.7398516711399600	1.231303300784520	-0.10873942632044100	-0.23093062098000800	0.04449569966770940	-3.4450925072295900	0.09494081407247150	92.55277485735400	40.052780823252000	-0.2703300676452380	-13.953642040128600
ODA1_T24_1	11.25	17.38	0.3692669029986580	0.44895253524786900	1.0056237427894700	1.245846920601080	-0.05735656685276140	-0.25058227328274900	-0.06975737737788310	-3.4484264092148200	0.07881581440818890	92.71581866932360	39.82411136635780	-0.2421884662150470	-13.916520507758000
ODA1_T26_1	10.92	32.95	0.41673768117871400	-0.26523975198237100	-0.03678919603945090	0.5001333984671080	0.02285715725560940	0.05029553592949600	0.31349370640308300	-3.213075877761950	0.06955919664112740	94.8154451617636	20.66196386898290	-0.6138020817519020	-10.859826068127100
ODA1_T26_2	10.78	30.89	0.4024676643182440	0.0816236125793545	0.3930274398794340	0.8790823970235530	-0.01572659556967010	-0.09704076125242290	0.1629420472760960	-3.36281183344321	0.1293070746788980	96.89778614314600	35.28393607026860	-0.26546902809390900	-13.239307124768500
ODA1_T26_3	12.6	27.36	0.3668543534997670	0.19484861848316100	0.33978214101187300	0.9569283043976050	0.20117717503003400	-0.11527561069733000	0.17944841309445900	-3.3514654353233000	-0.023174170867420300	94.40641962075290	35.115459428353300	-0.17930373405653900	-13.206830076657400
ODA1_T26_4	12.18	60.85	0.4196415001629530	-0.06504703946579640	0.632181430819869	1.3648721465134000	-0.21704213299959800	-0.05349894986796880	0.03642456626759880	-3.49988549346279	0.1470480550298720	93.99699450272130	45.066578040413500	-0.07942081703862500	-14.65367118602340
ODA1_T26_5	10.78	65.77	0.400053519246138	0.11638075859940000	0.5149395336900640	0.37310257737287300	-0.16949803655481600	-0.10281144797672600	0.10040582516154300	-3.1806391140978200	0.16070387226951500	95.22782701645320	25.263501669892500	-0.34741345956405900	-11.602310474332600
ODA1_T26_6	11.74	55.67	0.5045430853174180	-0.3077594403444550	0.49236276786929700	0.9101110236666050	-0.106485460220338	0.028285106523092200	0.07847508085875600	-3.354270027070300	0.12966309395935300	95.78541483691270	35.449210321770400	-0.21757591445031200	-13.274101553779700
ODA1_T26_7	10.79	57.18	0.4778835190037930	0.21463277162820700	0.49526122121646900	1.1171154540237000	-0.18584335035172800	-0.13957648110266800	0.12708127309983200	-3.430127868557160	0.169108709586466	95.11272412099280	37.905579347899400	-0.20653884425066400	-13.633299350873700
ODA1_T28_1	10.88	52.69	0.4141241726913700	-0.2074516327360880	0.3378702720352510	-0.007633771888004550	0.03824926200990690	0.03645263477498560	0.11608846932881200	-3.0719635515416600	0.10964598223998600	95.55181093004830	30.31279197373900	-0.08804762887472000	-12.503563109400200
ODA1_T28_2	13.25	61.38	0.389022174023444	-0.39489748762377200	0.6977054483073100	1.636309901436350	-0.11389723188808500	0.061193227530538000	-0.01670788048484210	-3.6299304755771800	0.20399281969098000	94.78696647010310	30.665918016660000	-0.21142589183775200	-12.587578550594100
ODA1_T28_3	10.51	53.28	0.4647822320051440	-1.0993565404384000	0.008946871727688110	-0.019705785546167300	0.17276050900673600	0.40742648356576500	0.11530206895696700	-3.0975260559000400	0.11139988364779500	88.1745339843284	32.61429475011940	-0.07597110475057840	-12.954778831686300
ODA1_T28_4	11.02	44.22	0.2860700771271530	0.36233601994972300	0.7279596541890490	0.4548647874684610	0.3101345744691220	-0.16063151151841900	0.00076243086230066	-3.2783364294229600	0.0716098805220941	92.62109836615050	41.76862789229320	-0.0386503775612020	-14.247561139730600
ODA1_T28_5	13.26	48.51	0.3410934594117300	-0.0883103310011254	0.5760929919499240	2.0139841278133000	0.3464518460036266	-0.02555711164461450	0.0258009919393978	-3.7479186621467760	-0.02737599365823430	89.70869124931930	30.4788101143993	-0.2759003320161280	-12.562569824624900
ODA1_T28_6	12.09	27.59	0.4800006668781810	0.0968579850613172	0.3584007804985980	0.7252041969339060	-0.0852220231463420	-0.0978593881870730	0.1659225283237190	-3.2849237336894100	0.1108854868412630	95.0636317014010	27.697221743218100	-0.3837153082894200	-12.039175126008000
ODA1_T28_7	11.25	38.24	0.4494131168854190	-0.0498193055356313	0.4225400005913060	1.0550098986272000	0.0919287514978230	-0.0655566846960589	0.1598731383322000	-3.4238772131571700	0.0730674374187773	96.7925937245147	36.70400621225910	-0.2427211050195270	-13.457352781941300
ODA1_T28_8	12.6	30.07	0.3927636102316660	0.3757842476532910	1.0128075614028800	1.0839904272371700	-0.3209975401763200	-0.2261067265348980	-0.0808620694655300	-3.4060975372942000	0.2187092920214430	93.2886755546820	37.89005612306490	-0.2125932176618740	-13.631837645311200
ODA1_T28_9	11.02	41.99	0.469559172469201	-0.0659634309805998	0.8255914793629730	1.265492293399550	-0.0685599301872600	-0.0813548746794308	-0.0294286235958812	-3.483927938182277	0.1353562457055800	96.5801380117660	43.8159249467314	-0.1911356628258340	-14.487201625654400
ODA1_T28_1	10.82	40.56	0.3349243984971070	-0.1947772932943270	0.0246448571676564	0.1694492142378020	0.9936595960446170	0.0728725412818926	0.2311179013617490	-3.1369287991801400	-0.2806173046586650	92.7213802511992	30.0340952669585	-0.0584114861025355	-12.528432552482290
ODA1_T28_1	13.25	40.8	0.5602506628140880	-0.2666802751961630	0.6866076011628240	1.4836394008638700	-0.1577616834577200	-0.00414890094575546	0.01203028420270000	-3.5492710341833400	0.1729477319302610	95.4610682453487	29.6948001379523	-0.3234344843830760	-12.437904596441100
ODA1_T28_1	11.81	51.74	0.3572588968711890	-0.5446356928073620	0.2765064861936190	0.4873081530774260	0.5005062768977650	0.1499849510297010	0.1122505158242840	-3.2466983393526700	-0.0808691428606699	95.2680149156010	39.6397847607185	0.0228023145363210	-13.9945752029815
ODA1_T28_1	12.67	28.9	0.4265398550804170	-0.0127574029848300	0.4425339968062900	1.0743025389173700	-0.1314594534746310	-0.0623482892932739	0.1333459740587270	-3.391569079252130	0.1121231768606300	94.5122731055123	34.5038532166880	-0.2608109506212550	-13.12599708604860
ODA1_T28_1	13.06	30.42	0.4614660387645970	-0.3854984652640580	0.5050306747993630	1.3761128797690500	-0.1894286510341080	0.0521403038935775	0.0786831275107809	-3.4927650534479900	0.1390365788102400	92.9676628067149	29.7291502007279	-0.3831462468123400	-12.449570197980600
ODA1_T28_1	10.54	34.31	0.4913291735117400	0.0119761155498114	0.2089223868852580	0.8025006860899230	-0.2536769355164490	-0.0432565597298690	0.2130761635339340	-3.3030264315027400	0.1400465099684120	92.5625308633349	30.4765308265853	-0.3455005965763260	-12.488592685739600
ODA1_T28_1	11.46	41.62	0.5486801869374720	-0.0150815641806020	0.4989018357687010	1.7114337989180500	-0.0971161114415925	-0.0528167863028804	0.0817720847088062	-3.6077516931795600	0.1011430310476600	93.5237404446907	49.4890815584604	-0.1190949478362000	-15.2544408664744
ODA1_T28_1	9.918	32.47	0.4549999074497700	-0.2783425906889450	0.8490270309106930	1.3990564122636500	-0.3887453039174100	-0.0248419992189912	-0.0495804729412748	-3.5103934565489480	0.2372175132165610	93.3269201132531	30.7361514170102	-0.4618799725565700	-12.6232797291025
ODA1_T28_1	10.52	41.62	0.4589927130111990	0.0840160706686940	0.1020604892628390	0.2914144059591100	-0.3761816668607110	-0.0577998574485175	0.2667308679121270	-3.1481595062164000	0.2425487529031190	95.2069971938207	16.1158793277650	-0.7411053261391340	-9.94917991685532
ODA1_T28_1	11.49	35.31	0.5214723291235710	-0.2887367887742190	0.0563573680002890	0.4753639405869500	-0.2375598945080000	0.0676835053330485	0.2520418661690600	-3.2015917714816000	0.2051920985901170	95.7240653176347	24.1302022884033	-0.4532985073020210	-11.43034517818890
ODA1_T28_2	9.988	39.94	0.4743156227737570	0.3787873216836840	0.0114971236472520	0.5232511521264960	-0.1477869807991320	-0.1691419135093120	0.3512654829254800	-3.2156930284174800	0.1100249139149570	92.8507012790611	19.9589841301307	-0.5946342429408500	-10.668639238049800
ODA1_T30_1	13.62	33.71	0.4539621076076140	-0.1528861546766800	0.3386346602488070	1.1709978149326100	0.0085002143137545	-0.0133410975246425	0.1667308514658780	-3.4389095782402600	0.0897742945189550	95.6079760420419	38.0164829621482	-0.1487629456657330	-13.646959699535800
ODA1_T30_2	13.53	26.53	0.4786284815427980	-0.1386770750419200	0.2645775280407750	1.1426891785645400	0.1556533566682640	-0.0138984418024818	0.2001450464444400	-3.4222956191671000	-0.0086361908185196	94.3423457535108	38.8186344561345	-0.1475950097064470	-13.7639885452903
ODA1_T30_3	10.7	51.26	0.5835000673549053	-0.1164175337329200	0.1948371618582120	0.0321109706980200	-0.0602801174302890	-0.0176578029687870	0.2304858979482010	-3.0657152385164300	0.1236535335884190	95.8194353210069	20.0773403273805	-0.4776354187074000	-10.663344269149000
ODA1_T30_4	14.12	60.27	0.3854124961262200	-0.6526986558414250	0.6431746772576680	0.3911310275004750	0.2170388412452390	0.2164790122740800	0.1502000000000000	-3.2548789083080610	-0.1003079272280390	92.1729982443896	38.4421888476710	0.2364272174594340	-13.809839712103800
ODA1_T30_5	10.66	61.27	0.3025273399590200	-0.0905331702737870	0.7447413643385570	1.0277818158514200	0.7242454014169700	-0.0148457059772790	-0.0757252663670577	-3.4213262906461000	-0.1957334998076700	94.7118254768687	50.6661519140647	0.0139430421017503	-15.498955188087700
ODA1_T30_6	11.05	32.86	0.4655418278245850	-0.0053003396092801	-0.0477164557115317	0.2126327016181920	-0.1383847012000140	-0.0294307603133435	0.3314069684938000	-3.0973165210592100	0.0872703629371057	92.7415938424274	15.8943201221178	-0.7525072396148790	-9.902088675331590
ODA1_T30_7	10.07	63.22	0.2849247946798800	-0.1003040476835800	0.4784243201309690	0.7588853741748590	0.9257577170314980	0.0247820276758280	0.0243395187715930	-3.3299056858040500	-0.2793920941140840	91.9286379671600	45.0820071444576	-0.0057679222308118	-14.758376273891500
ODA1_T30_8	12.02	47.5	0.3931273398184360	-0.3480663386961680	-0.6001360503922180	-0.3187717194385780	-0.0728943292522000	0.1397152353047230	0.5209588236015890	-2.9291849585748500	0.0960110960063459	92.1177338561005	16.4526015641430	-0.1524789531009770	-9.998640530074200
ODA1_T30_9	12.05	56.62	0.3736799162026090	-0.2164238494537240	0.0865368175548863	0.7468502627678460	0.5236257853616770	0.0523822765971468	0.2221928110603310	-3.2922061184254400	-0.1582120869440590	93.3551101585063	38.5312928863091	0.0470555991780992	-13.7958251596619
ODA1_T30_1	12.51	28.83	0.4846247125682200	0.0022951434423045	0.3066977235904640	0.6457354732746700	0.1808267804520870	-0.0664203374369984	0.1967893516634170	-3.1826612062208100	-0.1586982043922130	86.8808047858179	19.5431261997215	-0.5984518506012600	-10.638295126402100
ODA1_T30_1	11.36	40.98	0.4598005988197300	-0.2397817581560360	0.8286191631148460	1.9586121335143100	-0.4064927421478100	-0.0314260447135660	-0.0453414894466577	-3.7006633070532780	0.2631567738546990	91.1726449279085	29.9322043093477	-0.4132254744389101	-12.486255796661900
ODA1_T30_1	11.69	32.91	0.3990919694446700	0.3152111272591510	0.3396543659289070	0.5054148028870970	-0.3150259373561460	-0.1500082686011820	0.1827676040543190	-3.3519302066867400	0.1567468087449600	93.9386594845262	19.5920559163787	-0.5880433188023700	-10.615673757502900
ODA1_T30_1	11.02	35.23	0.4447697763256100	0.1972882179349130	0.6502957663171220	1.1243884320184900	-0.1645540424283700	-0.1210558116615000	0.0316862296440095	-3.4359083966528200	0.1788769674487030	95.0209633005520	42.0056340646480	-0.1685199706197810	-14.228172117586600
ODA1_T32_1	12.2	36.36	0.4269735568320200	0.2161738471158810	0.4308172912899180	1.3430657162153300	-0.2928628551840500	-0.1182553604741390	0.1346396796522410	-3.5010176882194200	0.2311857414570490	93.8837644516429	39.5148026120751	-0.1965952457046110	-13.871477403634600
ODA1_T32_2	10.76	65.47	0.3731730705647220	0.0874180967616503	0.3671367833006110	0.0530349364876662	1.0283867584914400	-0.0558476415826282	0.1130856298738820	-3.0781157918789300	-0.3489650637710400	92.6272259852508	31.3298174568490	0.0017782486884551800	-12.736827287875480
ODA1_T32_3	12.71	70.61	0.3630631420290430	-0.4264889054946160	0.0843238852150521	0.6032226771739120	0.8213424336798140	0.1128959981117660	0.2219201477607880	-3.2979741078997300	-0.1816299507526810	97.2635532213270	39.1338097096181	0.1524158483789290	-13.917697771089000

ODA1_T32_4	14.07	32.02	0.4387464081731300	-0.028978194876415600	0.6546156656906590	1.7779917683008000	-0.15557330957591400	-0.04533300279416950	0.01289881919501910	-3.6534180252040000	0.1549406023402150	91.46775567773120	29.80731746373480	-0.3225693628878090	-12.456439958791600
ODA1_T32_5	11.89	37.1	0.49007783902342100	-0.21429111335140900	0.36529684454624400	0.8685394757541790	-0.1691251805502260	0.012376920221346900	0.13710306481820500	-3.3303143731567600	0.1388624403389750	95.13817916234690	31.5624861449832	-0.3132753441567740	-12.675057241713800
ODA1_T32_6	9.452	36.06	0.35137009657228200	0.38897597096668000	0.6067114430423470	0.3789169680953880	0.04026925145289350	-0.21998866822569200	0.11542789004250300	-3.1954899750480600	0.11696919328148000	96.86395411554490	23.036779382419900	-0.514167636304422	-11.226837642185800
ODA1_T32_7	10.46	24.82	0.3993689638207820	1.0393473537912600	0.8698875784190750	0.648406370339406	0.0030491389511131300	-0.4182256761221040	0.020913299852239500	-3.2464570794139600	0.03942834028925740	89.769007166987	25.319501981957700	-0.436966030386518	-11.616653084308600
ODA1_T32_8	10.05	40.64	0.41584139470261600	-0.05065923585047150	0.7875277652939420	1.3466110437240300	-0.06140830172492400	-0.08686454387754100	-0.020658803792845600	-3.4896138676228600	0.10994002262032400	95.47372621369000	43.282022004101100	-0.22198405383988700	-14.414039389287400
ODA1_T32_9	12.07	34.92	0.46611152617937000	0.25400640293987300	0.6252222631172420	0.7018161664962670	-0.079751359830921	-0.16948195708045600	0.08198527106431910	-3.240310126460610	0.026801120613075400	90.54356253900430	28.624053495529000	-0.33076278542622800	-12.18390339332110
ODA1_T32_1(	14.57	28.67	0.363995603791893	0.14982224334761900	1.2018460568385000	2.0501353233596700	0.1263433133837390	-0.1426745459027800	-0.19011756215159900	-3.7469090956153900	0.043329041332454900	89.8068178165219	29.74889369767420	-0.3172496439314090	-12.446262034889400
ODA1_T32_1	12.68	51.76	0.5530976837802450	0.018760481851341200	0.5655325244246650	1.5621978611113100	-0.025239760605813400	-0.07519619109527380	0.06508248508354480	-3.560423639276560	0.08429614684797100	94.86301990944060	46.100455607739500	-0.05167266845612150	-14.794243181235100
ODA1_T34_1	11.61	78.34	0.3943675563113490	-0.7516384532600000	-0.3678043529069830	-0.2735137201215450	0.5454894697645430	0.25574804193560800	0.37345196293089600	-2.9498006514342400	-0.14363431469049700	92.52193886967110	22.77355408564680	0.042669369494761400	-11.287515264880700
ODA1_T34_2	11.72	64.52	0.5184944947756700	-0.13143786073794800	0.38248938475467700	0.6907055445968670	-0.13320331836197500	-0.03270604049177280	0.15511977059354200	-3.2807145973398100	0.14819199123935800	96.43644355200110	27.275506100520600	-0.315940177177441300	-11.979823102952900
ODA1_T34_3	11.97	58	0.5810088979941310	-0.05666493189095100	0.27553526361150500	0.40829786108513400	0.027122894163361800	-0.04562022926461480	0.20543939563465400	-3.175440614346130	0.05397683969306790	95.25378145814120	24.525802463677900	-0.3182513594681250	-11.472534881364200
ODA1_T34_4	12.72	46.82	0.515272681940742	-0.2858923876806710	0.7269740979054770	1.0801009089771900	-0.0384943783837073	0.00047952099842605100	-0.008632762463956730	-3.412250671126460	0.10836569393884400	95.9540351530062	29.686617913818300	-0.32867279891209000	-12.437059261593300
ODA1_T34_5	11.95	58.67	0.5160798120552150	-0.09846347343671240	0.6207914309114130	0.8057851671291730	-0.1454870426331950	-0.050927574489164800	0.050070265934716300	-3.339052547529670	0.177907687907573	96.80947286826240	36.244837893465700	-0.16596266291816800	-13.383438716274900
ODA1_T34_6	12.8	31.11	0.3735627384052250	0.3149423900089060	1.207262830998880	1.9124155166468700	-0.27323698841018600	-0.2100469295862110	-0.17304454690371800	-3.6612042919576600	0.30387606545175600	91.91251885542380	29.779109225754500	-0.39056939975792500	-12.45860083838230
ODA1_T34_7	13.23	35.42	0.4546747562748580	-0.360828980140527	0.4128604710241350	0.7815138792840140	-0.04353283459838400	0.04915196094074940	0.10574342020984100	-3.319075503487070	0.11542380798841800	96.09120672146470	32.99309367281990	-0.24644427171617200	-12.899505912656900
ODA1_T34_8	11.9	29.81	0.3812360447321050	0.4787201827454300	0.5357934802636810	2.292685017744060	0.708245298670821	-0.2386259918223430	0.12111001540624600	-3.787924488369720	-0.2477504500939490	91.43163801583630	55.09967198501530	-0.06529982533239140	-15.993062168194900
ODA1_T34_9	13.16	50.86	0.4562091163506530	-0.13134010934846300	0.9453024376689920	2.5429193525093200	-0.2555812709908740	-0.045635973329686900	-0.1144063991243900	-3.905647797411310	0.2156844513984260	95.31109107378410	64.53021790318420	-0.006982495087282610	-17.129771848088100
ODA1_T34_1(	11.93	51.61	0.3458531951756560	-0.12191091551005400	0.5052374676161770	0.5145712005863460	-0.23200147461475600	-0.025571297571321200	0.08288019126927260	-3.2054573737366800	0.14204901071496600	93.14621445801370	27.943392893914400	-0.2958728347167140	-12.068879486340100
ODA1_T34_1	11.19	29.64	0.3418133308125670	0.3734488429235830	0.4431762039793160	0.5329078976296730	-0.046196813951140700	-0.19794717444695500	0.17088096153639100	-3.2201837486962300	0.09650782607718190	95.14317847250130	19.35447523651920	-0.6226481601378050	-10.573564666035700
ODA1_T34_1:	12.49	51.12	0.5044440543729950	-0.14652647828273800	0.32151905576653300	0.8311859911797530	-0.0355994551101346	-0.015440323005211400	0.17234381144590500	-3.3191694327787200	0.0829299655189801	95.17917341344670	30.84880668888870	-0.23137697422166200	-12.547658247811300
ODA1_T34_1:	10.02	52.32	0.5547049091245190	-0.14330486246446300	0.04758205693425840	-0.3816772388791940	0.1038484675557720	-0.014754292171224900	0.3149573470170130	-2.940447943368490	0.08118080160947570	96.45869709730320	14.414002361243200	-0.5266658211316010	-9.665007209257940
ODA1_T34_1(	12.56	19.21	0.428125014238867	0.6176480933947830	0.17596169126766100	1.8774031616835900	0.35788738757285700	-0.2285603070373940	0.25594163313078500	-3.6590636242582900	-0.07724427564058190	90.66898114590150	47.73183508512330	-0.10025561879399000	-15.030775504958700
ODA1_T36_1	10.92	33.01	0.41705735452289600	-0.27224743395896200	-0.03762113546600280	0.4992261080115710	0.011037732257267100	0.052279604648230800	0.31373870456015100	-3.211997731776870	0.07244081626227040	94.73453262532630	21.00960517581200	-0.6052553084011110	-10.925811764482800
ODA1_T36_2	10.78	30.89	0.4051056567938260	0.08219642079364160	0.3901227418818560	0.8788393109165530	-0.018540926861996300	-0.09700090974743340	0.16417228284472500	-3.362404520554690	0.12969011253748200	96.86761524170140	35.2134989792327	-0.2667390038961150	-13.228607092523600
ODA1_T36_3	12.6	27.18	0.3681020322508550	0.20941180574747200	0.33936880654392900	0.9769771872476610	0.1980619876587310	-0.11979858446854500	0.18099636501552800	-3.35832247879756	-0.022310745948403900	94.33716874361510	35.461545243538000	-0.17673927528937300	-13.260049139285900
ODA1_T36_4	12.18	60.82	0.4166403642415200	-0.06597764935194600	0.6342946073677450	1.372768660145620	-0.22095765650161700	-0.05323699406660490	0.03532208865027210	-3.5022840574978900	0.14816881627429700	92.62517760239620	29.904549489087000	-0.3079825992511230	-12.471106810929100
ODA1_T36_5	10.78	65.65	0.4049295810460430	0.10973829709321400	0.5108993583052720	0.38007270976702900	-0.16373427587997400	-0.10066589488583800	0.10151559617323900	-3.182678744511110	0.15779068800463900	95.24366249800870	25.454365687329300	-0.3429896375170040	-11.63515948748650
ODA1_T36_6	11.74	55.71	0.5009140971549770	-0.30278428745778400	0.4951501889030170	0.9929505253384390	-0.13602495976740500	0.02639793707825850	0.07770007303270640	-3.383838813230890	0.14757949402277400	95.99034848277780	36.661238176783200	-0.2066456403631490	-13.457185863953200
ODA1_T36_7	10.79	57.08	0.47448868943004800	0.21378007788551400	0.4963737799387060	1.1147805552874800	-0.18463044830012100	-0.13911686025266000	0.12621349332941600	-3.4289377135152700	0.1676648406738930	95.08424884673460	37.91237449941720	-0.20626719812190600	-13.634220836788600
ODA1_T36_8	10.36	43.44	0.39319904824820200	-0.3034153471629790	0.6941960238297150	0.5608417496078200	0.20612805713275400	0.0018415721817463900	0.01420102139487840	-3.2559870032493700	0.03699075014726520	97.05250377510300	35.50804346456	-0.22113042939676700	-13.309659747417600
ODA1_T38_1	12.18	30.9	0.343263355430254	-0.07598445357741590	0.3122607626324200	0.664574667407557	0.06644571626692480	-0.06345394026988540	0.21155705814377000	-3.265388717391750	0.05407718709124500	93.71846844267950	23.745299919704100	-0.5095187257420510	-11.42583763428630
ODA1_T38_2	11.54	57.56	0.4468434055691770	-0.08180857652723580	0.7233660038547120	1.4608458790400000	0.008584324779138670	-0.06884367684953820	0.01200963878553360	-3.5281638978840400	0.058919528279394100	95.21948125402330	44.6971425721817	-0.12273907456467300	-14.604706275030900
ODA1_T38_3	11.09	57.66	0.438909367402197	-0.06798186661594070	0.5149640089601600	0.41817085503769000	-0.11832181517031500	-0.05559184745849060	0.10165031499303800	-3.201203819667710	0.15617300131438700	96.41668102258480	25.507785543178000	-0.38265386299215700	-11.667009875387100
ODA1_T38_4	10.85	68.29	0.45159582219466200	-0.4809193398204280	0.2944388250638440	0.4945138840730270	-0.12769112717290400	0.08932001225967030	0.16420755502669200	-3.228722224992500	0.16674826736239400	96.53751582710930	25.10461573205380	-0.439217013835583	-11.662474521940500
ODA1_T38_5	9.977	54.54	0.3277327654891720	0.452341760346132	0.6773453776473870	0.6618396243370870	-0.2654641199691130	-0.23612912987142000	0.07921502526594890	-3.2736816615765900	0.18435244073616300	93.4073650630229	30.743865560212500	-0.3182634912160720	-12.527560272224300
ODA1_T38_6	10.23	65.81	0.4773421859272380	-0.08945477702091330	-0.021561621788462900	-0.16294738236692600	-0.39425444368968200	-0.00823752955529439	0.32463491982873200	-2.9955343075028100	0.2568355330326470	95.21769160939270	13.41505279244670	-0.7839020880954200	-9.370583592483580
ODA1_T38_7	10.91	50.56	0.3724665653285540	0.20402245941780200	0.9556889324362080	1.1805386130793200	0.047670163682840900	-0.18581790419208100	-0.046625908018443500	-3.4638109700516700	0.09975923805048050	96.20162628090280	43.55272937047480	-0.16423646639843900	-14.446710650443400
ODA1_T38_8	9.983	60.85	0.4186411338828120	0.06820022705207910	0.52751139353363	0.15294727436618100	-0.1976519688526670	-0.12305683672313500	0.13311788176905000	-3.100829386488520	0.1726490995829260	95.15703684068720	16.977326094676800	-0.6929851538838770	-10.126800192281400
ODA1_T38_9	11.73	59.19	0.4351381392893720	-0.25348227230686200	0.3501447420301980	0.39865745077907500	-0.07077454592374560	0.019515141154155800	0.1403527457751530	-3.173687937233580	0.09952152156548300	94.4464030444407	25.75319904824900	-0.33029162498706100	-11.702324249715900
ODA1_T38_1(	13.08	38.14	0.39504039574688500	-0.012822212732316100	0.15218681128099600	0.28905376410700600	0.20704661611423900	-0.061684654162884000	0.2753228636157870	-3.147510046750220	0.006636145886731730	95.95041301724560	21.20723061340130	-0.37197050329644300	-10.855667069195100
ODA1_T38_1	14.55	51.66	0.39899194557944900	0.05081272022958350	1.1793632427340400	2.0041674825720800	-0.09910806572868980	-0.12415732329864100	-0.17086377898164400	-3.750961023097620	0.15635688171312200	88.40683649421290	30.60859868029550	-0.1443676248891730	-12.572829540526700
ODA1_T38_1:	14.4	53.33	0.544039198639949	-0.5617326286480260	0.43879249082415	1.0774437234232400	-0.11095737411458500	0.11291312351191700	0.08790036614389380	-3.4127452006869100	0.146570307361487	96.0683816436665	30.609897163691800	-0.14790213823305300	-12.573313686403400
ODA1_T38_1:	11.68	50.9	0.36961532856474500	0.12567112065963000	0.6690834328008720	1.1890603410896400	-0.19949775508223000	-0.12329714284913300	0.04727200476590480	-3.4542245990139600	0.17637011667943800	94.99593605368150	42.16646153806600	-0.1445089127894920	-14.249253683972600
FIC1_1	12.1	97.01	0.39272633310523500	-1.1893828892848700	-0.30031042279463000	-0.7046034707536200	0.39140672142879400	0.4007078705063040	0.31747151814768400	-2.8292033207149100	-0.02301088875764310	90.12857670065200	19.069773819844800	0.16616663595905500	-10.598703727554600
FIC1_2	11.15	97.43	0.4525033469750000	0.19167062158409400	-0.2974660635392370	-0.7382841272204350	-0.2507164666981130	-0.06449489853812310	0.44772909768930100	-2.805544700892680	0.21091340922384700	94.76344040512090	5.923498570066590	-0.7137197353112000	-7.502188967619750
FIC1_3	12.91	72.57	0.4622950333055840	-0.5703204699309610	-0.1750219974866090	0.33863799449243900	0.19969302506412900	0.2074990168132020	0.28229293322497600	-3.152994838790730	-0.026085794032346900	92.0541525120371	34.07542248960910	0.2063190835887430	-13.137675531593600
FIC5_1	13.66	39.17	0.43073579792845800	-0.37226605397593100	0.08067805111049270	1.0597980187784300	0.6770876282276400	0.08224223724760450	0.25509178042924900	-3.4098355976295000	-0.18286025732176600	95.33068047645780	44.86036662338060	0.1907032226071910	-14.712854181853900
FIC5_2	13.63	34.03	0.4875820948026850	0.1659918999728140	0.1373753465451880	0.8712379201033880	-0.09596295524092880	-0.1314963420396200	0.3165515311619780	-3.3472728878066000	0.14198630350114700	95.83147458267830	27.391483212280000	-0.17515345743653300	-11.968332641162800
FIC5_3	13.64	34.59	0.5093344068300050	0.12398609806740800	0.03407935221852830	0.7305060552613760	0.29045547084306200	-0.1152407498577010	0.36207728120672000	-3.3020925649187200	-0.01759054222070930	96.10397271313180	27.676415793616700	-0.10620280527664200	-11.991746639078000
FIC10_1	12.91	23.36	0.49496881541955300	0.10338754832858300	-0.22094755378379100	0.8112579792571370	0.1068562752536880	-0.036609533650792800	0.3941646279716540	-3.3036532766891500	-0.0073371632911500100	92.00161970853940	30.442348198565700	-0.23087483190781400	-12.463490410524300
FIC10_2	14.07	15.94	0.47508014819782900	0.2550567404347000	-0.19692153731553000	0.9716657194549040	0.017112407636688300	-0.09442831955996120	0.405425945332696	-3.359792562322790	0.031090862352833600	91.65241694184530	31.58979256464520	-0.23425376366374700	-12.65016641896470
FIC10_3	12.86	23.05	0.44233553688151400	0.24802160361361400	-0.026876881957027100	0.7150045263949470	0.15136144005414100	-0.10361540129466600	0.3299546443497250	-3.283741955226910	-0.006853514278162620	93.43124369923800	30.000081473685200	-0.24395328445919900	-12.391445747389700
FIC10_4	11.66	14.87	0.3909637048815060	0.11876010448806300	-0.12356383764966500	-0.37964331330530300	-0.4286140345266770	-0.04872119645051460	0.35868926859823400	-2.885156875998600	0.17299380641377400	89.31296196513720	12.419604570041600	-0.8052960602330260	-9.030726314920270
CA2_1	9.391	87.59	0.38086463281453300	-0.32628895750373700	0.2567481504580100	-0.5352467779012880	0.29345576726614100	0.06480400481872530	0.1566152723490730	-2.905085154725650	0.04098711003758340	95.45105905286720	22.203840946087600	-0.11491758930302500	-11.184014351421200
CA2_2	10.92	99.42	0.41493763612323000	-0.48101282671922500	0.33603444312157800	0.5659310104835870	0.44664630805956600	0.110539925920608	0.10253207896691500	-3.246298795677490	-0.07048771469245920	96.35782065708820	39.74443178185200	0.07821973612063440	-13.998486697103700
CA2_3	7.931	98.23	0.48516618476287700	-0.6393248278978880	-0.4208337775072840	-1.2186501804416500	0.29971875726683300	0.21787764244743900	0.4171355294075120	-2.653979565198450	0.00165750647095661	91.0732270897287	11.523669648566500	-0.32665056451495800	-9.00601651333308
CA2_4	14.32	89.12	0.36980256642024000	-1.5696421402188300	-0.6798333069621390	-0.031119215605978500	0.8054450654117860	0.5531652617501470	0.4285547024410500	-3.011042432313620	-0.3077707286929480	84.4135788163881	29.562224214643400	0.45565169147151300	-12.462826186153800
CA2_5	15.07	67.22	0.5193648625694320	-0.6342883052065040	-0.36784389460658200	-0.17489928731585700	0.015832499869540300	0.19846933722991900	0.40600551573393800	-2.9815253929077300	0.07385413364053210	94.06423909395090	15.63851612856700	0.07143271232447050	-9.879804240650270
CA2_6	15.22	66.09	0.5333279064941710	-0.533826632631293	-0.4012995379112640	-0.02804404081707380	0.04873529569609	0.1744181775601340	0.411867642190698	-3.0262197812565300	0.04686545084789180	93.58431633832180	19.378335687073700	0.1931567171547060	-10.563554211174100
CA2_7	11.12	92.88	0.4473890430157320	-1.4414167685740900	-0.5671170336210680	-0.23650434558973700	0.7314835535605890	0.5031757476021280	0.3951980953296380	-2.9736937441848300	-0.20044195934091300	87.88201099805130	25.827263188219200	0.025365342883658900	-11.843974071506700
CA2_8	13.23	93.98	0.49235298111746200	-1.3314654957307600	-0.047935213021533400	0.45549686010620900	0.2830484072699030	0.4099478082364580	0.19809362037533600	-3.207147476767460	0.000321346603886288000	93.37929279397530	33.093683540474700	0.1550042547757820	-13.023257316783000
CA2_9	12.83	84.98	0.46759152754364400	-1.4960719460833400	-0.41389870756789200	-0.013846762938239900	1.0086500996184900	0.49544295927692700	0.33148230527519300	-3.026254430078330	-0.3481613170416370	87.0795335114423	30.101806765664300	0.17406050438553200	-12.555225355513400
CA2_10	15.41	84.23	0.4143331341483410	-0.49772585964591000	-0.04981783817260160	0.4311493888453160	0.06387047022625990	0.12425500408783500	0.29087954605149800	-3.1737486828367000	0.016866447494684400	93.63579013201720	28.20924173599400	0.4458382722236230	-12.0555055558118
CA2_11	11.14	91.26	0.5095897200599100	-1.2288673376167500	-0.3734467282020260	-0.06420038313293400	0.3878421471907840	0.4016240941801700	0.3505193458008600	-3.0253529002234100	-0.06102796156829320	91.4424341454782	25.85129614256620	-0.025844804379297400	-11.848681897000800
CA2_12	14.53	78.73	0.4063222067710490	-1.1772944327079700	0.13264773126390200	0.6762674862608590	0.4985378924918100	0.35260384386622700	0.12070897177376500	-3.2731628996675900	-0.11169305978513600	93.3982559831194	40.42934983459110	0.28778864548769600	-14.12338798208100
CA2_13	12.63	101.4	0.39172114163068100	-1.1107769872975100	0.039784157650572	0.4513098207275860	0.4131754602254970	0.32956499671732300	0.17360343292383000	-3.214365846320690	-0.05383760061779080	94.37245719194870	36.39517543404320	0.15405024791010000	-13.530171341169600
CA2_14	13.23	93.59	0.3406934928789600	-1.3281716572987800	-0.03731390449416860	0.4580833864541860	0.2702112429022220	0.4070746283773990	0.19531707904660800	-3.2085477151027000	0.006471567538482370	93.49251602905670	32.96964404866790	0.14938828205053700	-13.003527958589300
CA2_15	12.83	84.09	0.47841081326179400	-1.4804024346431800	-0.4194827519388710	-0.0022725845180703000	0.7171387474065390	0.48940292269854800	0.33540126750823900	-3.030965369251200	-0.2198145630707420	88.72410591396430	28.868908835076200	0.15123597104528500	-12.353410331441700
CA2_16	12.83	84.09	0.47841081326179400	-1.4804024346431800	-0.4194827519388710	-0.0022725845180703000	0.7171387474065390	0.48940292269854800	0.33540126750823900	-3.030965369251200	-0.2198145630707420	88.72410591396430	28.868908835076200	0.15123597104528500	-12.353410331441700
TAX_1	12.57	79.18	0.17924471560691000	1.564018793383360	0.12870224777165500	0.0905365457079632	-0.3981146291678420	-0.5038784518949930	0.329031601890994	-3.0823614539847400	0.2567570788764560	93.71014341549840	3.6015165217402800	-1.5410067141857000	-6.538195519766720
TAX_2	12.61	77.47	0.1554606608636150	2.0103105768098300	-0.08097022019254140	-0.12209237473390500	-0.7090009258498690	-0.6108199048010840	0.3961753878133910	-3.021210776093220	0.3774401285118500	93.3596692230944	1.699455738132530	-2.1416353621752700	-5.765817774935640
TAX_3	10.92	81.73	0.17921930005373100	1.8597212567570200	-0.05885264206315440	-0.5809310913452000	-1.1372616919520900	-0.6185715912435730	0.4790517927794550	-2.8514618389356600	0.5308782877632620	94.20020930619000	2.4955830432700600	-2.0976004332478200	-6.113521853249640
TAX_4	11.73	113.6	0.1333073136510930	2.737769444187950	-0.7090442433677480	-0.4024992525177140	-1.1394315493606700	-0.8475993033493840	0.7645996663292220	-2.9015435323238600	0.5284697108889030	92.87003594790490	1.8829419421040900	-2.068815073665500	-5.7638597288115400
TAX_5	11.77	126.4	0.1339452142522450	2.4917615692209000	-0.7698438614756180	-0.5260619707745750	-1.3386059700418200	-0.7848381359309720	0.811896559677287	-2.8579600888608800	0.5985498298964990	95.03591306858890	-0.7031639161048610	-2.604612153959570	-5.192547439683620
TAX_6	12.13	131.9	0.11857817647575500	2.240652278654350	-0.5816985586135790	-0.11678589443217100	-1.2966702351599900	-0.690595590105007	0.6777446575559140	-2.9838303632207100	0.578748459689786	92.20050886524830	0.9195670497211950	-2.2603099613655300	-5.3285492329684700
TAX_7	11.94	132	0.1507146249652250	2.545262664417560	-0.6578094871401020	-0.9108749254851620	-1.5517350371313600	-0.7759526536024590	0.7111911841228350	-2.693802059037000	0.7101807064140940	93.36649870339350	0.5004744911202290	-2.726743620373050	-4.997232369837760
TAX_8	11.3	119.5	0.21475500791635400	0.9648828258976780	-0.649535269437506	-1.5287145945408700	-1.0768026776079200	-0.2752908787559200	0.6283446809943680	-2.5825521424881100	0.636617152319159	95.71101156976340	1.5289175684198700	-1.491342405263500	-5.654064517975730
TAX_9	13.01	67.57	0.13366012554475100	2.0004094327841900	0.49915891424355300	-0.594755176307811	-1.2369123826359100	-0.6903035256304460	0.2119025617473940	-2.8044265556820000	0.6028576925337340	89.21618714524400	1.7778604280867400	-2.122141279133210	-5.794551024334090
TAX_10	11.25	127.9	0.14075735927941300	2.405179610966330	-0.7934093737472710	-0.19346297077717100	-0.9895950780694940	-0.7249250343104220	0.7565958587449090	-2.9582105631184800	0.4386972246457750	91.25883080559210	0.8255721976673660	-2.613746856176430	-5.32372286292113
TAX_11	12.27	80.27	0.2219832402472470	1.8566886618680800	-0.25504758763057000	-0.11307395874278000	-1.102786851999740	-0.6132873003657740	0.5435262991871420	-3.010984467056740	0.5617038229013100	93.09750801442550	2.5746809759368200	-1.8647211123573100	-6.1375319547178400
TAX_12	13.24	161.9	0.13771309668607900	1.4092702255886900	-0.06164648561926490	1.2240257156958700	-0.8719973958174200	-0.4595212468347760	0.4074846794885070	-3.4072915665077500	0.3616512363926790	78.96517522647340	13.415527097440900	0.042721067696591100	-9.256953273501510
TAX_13	12.13	119.8	0.18661771257366700	2.135404896245970	-0.3545800905606630	-0.5389779752677920	-1.240506000368900	-0.7038252105983310	0.6151553906386460	-2.8386037224308300	0.5484297684468410	91.08795249897660	1.3361191222235100	-2.091733996564210	-5.58298115290543
TAX_14	12.1	122	0.18820499515096400	2.2899323767864100	-0.6811373203603310	-0.27805077753391600	-1.1508372464254900	-0.7289872927216010	0.7579387633236630	-2.9371909323542800	0.5183221769109610	91.99887298625690	1.0847676458249000	-2.2265221790158900	-5.446797406820940
TAX_15	10.53	165.3	0.10850936621732400	2.6426781985139700	-0.6036869357888180	0.236913540161711	-0.8254587397411700	-0.8056931872177910	0.6966412426207310	-3.0665145759715800	0.2683634841918340	89.01019858180460	0.6228434732162	-2.8296190640770400	-5.172024218468080
TAX_16	10.78	142.6	0.07803636472617960	2.9761225141378400	-0.5448147999313760	-0.28203805445006000	-1.1649243656169000	-0.9579357916436050	0.7377292770621740	-2.9669831398676100	0.5697550208795560	91.56801196005660	0.6103714776814980	-2.887083032162850	-5.169754516943290
TAX_17	10.75	131.6	0.15202819615440400	2.1805560590835900	-0.5432653572536910	-0.3276979772565390	-0.8647389067044350	-0.6772674399717930	0.6560077880378410	-2.9035311611846300	0.36106260186088400	92.56927441499280	1.1649022862197000	-2.467152915928450	-5.537661320292970
TAX_18	12.66	115.7	0.1261735518406790	1.7448122955641800	-1.6331758774359000	0.46394509878175800	-1.0898675781219400	-0.45685480467082900	1.0848822626097400	-3.099696851840610	0.3096060427332360	70.9631459640385	0.9603488552427450	-2.1760524790474100	-5.366098586778700
TAX_19	11.01	123.1	0.1256830159820040	2.544967113539290	-1.168223236587390	0.16226930050175100	-1.290751236543660	-0.7409151032328800	0.9211940289836870	-3.057843714102120	0.5380858494943320	79.21971399153140	0.5711473948185420	-2.9618300536174600	-5.168296416481690
TAX_20	10.28	106.2	0.1913617865513980	2.0028685999419000	-0.7418717046223910	-1.6616078032290600	-1.1998614057535900	-0.5409623461070360	0.6543103962554410	-2.5111739000457100	0.6333360978741580	94.5973842345271	0.5981103356106500	-2.772624314772120	-4.913234388338540
TAX_21	10.5	87.23	0.20294290566505600	1.6687747384743300	-0.2622283602786230	0.11912299438155600	-0.9722842538448460	-0.5308677011290940	0.519047042156518	-3.0846333415499100	0.4726211861375560	91.3530061579752	3.7307802510392400	-1.7540044118726200	-6.617486723900460
TAX_22	9.941	86.86	0.15050200557482200	2.3754923164747700	-0.517302169447264	-0.6017145453825350	-1.1076707868884400	-0.7157411759111840	0.6339133542080880	-2.835134925369090	0.5201360498943190	93.61311545446230	1.0440253118842100	-2.8504143218043800	-5.550974766424740
TAX_23	11.65	132.6	0.110629421572775	1.8684745453687600	-0.8329786144789490	-0.5870504012243880	-1.6395659829358500	-0.5546157353083920	0.7243569594855770	-2.8236721684504600	0.6991339355681880	87.63988478412190	0.8971461884184300	-2.4143730754029800	-5.344265676450850
TAX_24	11.82	115	0.16260225480434300	2.377433241849270	-0.7905774412446530	-0.8179047173078380	-0.9026379792924850	-0.6902847394413570	0.742663506642169	-2.7580431749910700	0.41282163809580000	94.58167614843640	0.6367371369941950	-2.7908202881088800	-4.860712035085930
TAX_25	10.6	132.2	0.2172999514141340	1.9866945647576900	-0.35140203568786800	-0.7341212423821240	-1.0040374143083100	-0.6350566542282810	0.5765986875917130	-2.7697128979150600	0.46327537199017600	94.80978358475940	1.3984006930292800	-2.3732514322319300	-5.646885578405400
MBO2_1	10.88	37.22	0.8994718769863710	-0.2167567120218030	0.21614820203874400	-0.3375924419483430	0.05917254936638990	0.0029114252475419600	0.23183993323352200	-2.9658041827653800	0.11886053684669900	96.80829103995050	17.665747923626200	-0.4571634264636790	-10.337924293308100
MBO2_2	10.56	43.37	0.7667339907336510	-0.7973100106252470	0.04635663924338120	-0.8144776782826320	0.13767108645648300	0.20900691301073000	0.24938831256969400	-2.8058415347202000	0.0843825172092734	93.57835630562450	17.203475371702400	-0.2914533722951690	-10.256516301298400
MBO2_3	7.875	31.19	0.6644771206764050	0.122998234833473	0.28445158510397700	-1.1494634748407100	0.2611616199997960	-0.10600496580393700	0.21905128050455600	-2.699035257340000	0.042056685499321200	93.63003413424630	12.439942979341500	-0.4037474791055040	-9.204113152839270
MBO2_4	10	23.29	0.6564778903250600	-0.35119067441421900	0.054472595071832700	-0.9391817177448000	0.5478141683324340	0.053428998698414000	0.2884555300217260	-2.7368044245501900	-0.14728550822233000	91.71732382851090	15.521484958495500	-0.2916783150070470	-9.895894189658930
MBO2_5	9.578	41.22	0.7068402860785230	-0.1397714573313220	-0.22954360011677700	-0.6197119278745010	0.14113401098719500	0.0029622707694901000	0.43942419092289200	-2.872708111250370	0.08826805921169180	97.14054748120960	12.72530710632460	-0.459165296458029	-9.283017192907370
MBO2_6	7.624	24.03	0.7827483220862350	0.14475366644933300	0.09824724501631480	-1.3205660822182700	0.6056186204937550	-0.11986950712283600	0.33063285681328400	-2.6330487643215300	-0.12901000866331200	92.2303967880994	10.827712365230000	-0.4237288930863950	-8.837308285010890
MBO2_7	8.503	40.64	0.6623030089154300	0.010968356215054900	-0.1378308938666230	-1.319917562954520	0.4423740129647350	-0.075209964255244400	0.4409597582053460	-2.6622186735385330	0.007407608300200100	95.49362393805550	9.11209524404422	-0.4692100996947260	-8.408787760215750
MBO2_8	9.654	36.45	0.7292509628293070	0.3467395245300840	0.01244546983572230	-0.9811037447737530	0.2331255007412700	-0.18706227220425900	0.3983659712916300	-2.7650948495111100	0.0758169294404200	96.67945634027850	9.00160225758960	-0.5460521003151050	-8.348030095654860
MBO2_9	10.62	44.19	0.7229944610631450	-0.5589649252369380	0.2533946674176080	-0.9106967054967860	0.4855305741795990	0.09292044014547470	0.2116784645323700	-2.7666434414840900	-0.0752474925988468	92.77693348082230	17.082084863648900	-0.2581402491003020	-10.228851687289900
MBO2_10	8.016	18.71	0.8252439073338690	-0.3817159914243230	-0.2208171860830860	-1.2055141232121400	0.30322835209149100	0.07132076749350280	0.4212048654574010	-2.7123371357217200	0.0954946011324170	96.13085186640860	11.151837367092000	-0.4312816115632320	-8.9268769062687530
MBO2_11	9.358	21.29	0.8401188351367390	-0.3066718019179380	-0.1315961301894730	-1.0475644546877800	0.2543769497849700	0.04669413752218200	0.3857032063782770	-2.7363420132576100	0.05361165733150450	95.569724342038510	11.957503942256900	-0.4054399726686600	-9.110331951168790
MBO2_12	9.428	38.21	0.7091361678102820	0.2005289031386510	-0.04960825703628200	-1.1118716649318800	0.0751714365608050	-0.1220864192878310	0.3897337289753470	-2.7274824290343420	0.1712142929608290	97.08782909901010	7.976736173507740	-0.5972900502391660	-8.093042268740370
MBO2_13	9.718	18.09	0.9712633494558180	-0.3586998021287640	-0.1279021537632600	-1.1189410327149500	0.5416186287807090	0.0628805688930060	0.3790971986699090	-2.71077788581850	-0.07192631827828050	94.52230799280700	12.876955543418100	-0.3522807891608710	-9.349296712430220
MBO2_14	8.972	33.03	0.8012255908289960	0.013334360507590400	-0.07350554854269610	-1.4382420494528800	0.0802912274363470	-0.07123723303373240	0.3982105889837270	-2.6323596892580000	0.2043755547876300	95.76374063229020	7.556758018138700	-0.5830602341256720	-7.993113556273390
MBO2_15	9.399	20.68	0.9248868177163520	-0.5074448142218320	-0.1846287867964050	-1.2905070595777900	0.5547399552472650	0.1043327387872600	0.4118312380615230	-2.6638471538486000	-0.05432266147781400	93.99082971747420	11.750462770510800	-0.3781953063205570	-9.071638901330780
MBO2_16	8.391	40.77	0.6724504488759800	0.024271883291203500	-0.05651476438481440	-1.3672406233354500	0.3362524641085110	-0.07326915854443220	0.2850659483983010	-2.634226676276151560	0.04336002973119880	94.62344457463360	8.649652311304800	-0.5075191528845310	-8.287468461380830
MBO2_17	10.24	19.92	0.8900041422977360	-0.4753922217426810	-0.20791595045861160	-1.1318741395553500	0.3534252887632430	0.1015493627725350	0.4111519991085880	-2.713144988833040	0.0259142860612090	95.15540195603210	12.04338870262650	-0.3751491664608200	-9.135301148129780
MBO2_18	7.484	37.94	0.7668616508066430	0.0030780694902300900	0.04915327776687980	-1.0095392840549700	0.2673648146676320	-0.056740469114789900	0.3256034921555950	-2.7534640218358400	0.05421466006893900	95.8360352956866	11.756267295517600	-0.4174353147208600	-9.047093325738470
MBO2_19	10.91	38.44	0.7808615991686960	-0.7391856995296420	0.006964947426298850	-0.7474137312180570	0.2509326857066690	0.1756257318205950	0.294788954774649	-2.8329889170396790	0.04303372650048130	94.82654511565540	17.39306205688250	-0.2775323006047180	-10.293291766596400
MBO2_20	9.155	23.38	0.8274662253744270	-0.5795462092436480	-0.0361891872361620	-1.195316679795640	0.008943638904597000	0.1464783059414000	0.4469850638417700	-2.7085948761262900	0.02790138717335080	95.0938358092150	10.72643069494900	-0.45187161215978400	-8.829263624720880
MBO2_21	9.604	37.61	0.80455543992032	-0.2059011932826700	-0.05427364345526500	-1.2977375221358300	0.3344678619574870	-0.002984343448424770	0.37562011767099	-2.67346977747750	0.12068238786994200	96.6250751786730	10.539775914238400	-0.391031725261066	-8.771929770890170
MBO2_22	10.2	21.69	0.9450374728864150	-0.7345217319409750	0.2046938986218300	-0.6025648848850310	0.5201747122687960	0.1732439881990600	0.18571762344475600	-2.8696994900869000	-0.09950072728252010	93.3949202890051	22.25542559740440	-0.2655005617822230	-11.22530990638100
MBO2_23	10.95	45.7	0.5966180091977540	0.2017747389252450	0.17714432133006900	-0.4626468893195140	0.2358388372650130	-0.1404248028122900	0.2973198360249270	-2.9384188445524200	0.07663856596815390	97.92758939096330	16.876395276176000	-0.2928688059540140	-10.047191778235600
MBO2_24	8.77	22.92	0.8101633369289660	-0.36823706634717300	-0.13682475387890800	-1.0470824379558200	0.4206330676582460	0.07468059781347800	0.3749234007607050	-2.732995558094750	-0.031754334581319200	94.48344679912390	13.514941310840700	-0.3471217983020220	-9.4636856350385
MBO2_25	9.098	33.16	0.7032043003038330	0.1836860714282160	-0.0373467444153180	-1.026427672366550	0.16778490380756000	-0.11836028409654400	0.38465755091334800	-2.7595866628580000	0.126913890902714	97.32233984561530	10.00514702548130	-0.4937118705528480	-8.614646439972200

**MBO2_26**	9.111	23.78	0.7838759058576270	-0.41509094710217300	-0.05750522415027370	-0.929783905697079	0.31365368427510700	0.06500580379222370	0.36351286518548200	-2.7815788907524700	0.03531907210167770	95.55472719389550	13.697522838847600	-0.3791063366794990	-9.511787786904660
**MBO2_27**	9.427	38.23	0.7799908651775340	-0.35152267655714800	0.11036742465335700	-0.839653485563341	0.2858759353032170	0.04192425723912620	0.2782202468524520	-2.834577340572260	0.09951780019325060	97.31193200120670	16.06234086353500	-0.32871382703952300	-10.017397820979300
**MBO2_28**	9.941	19.68	0.9821808037261300	-0.13816244200153400	0.04226100406664680	-1.060539126393980	0.2526284767212150	-0.02045901151556980	0.32637611855202700	-2.738065213035540	0.06810080487697730	96.06697204246550	12.248547451584100	-0.4022028224438320	-9.174309287892680
**TPG1_1**	13.62	44.29	0.707551426290921	-0.04864583198547320	-0.007127136157835750	-0.08805736080275200	-0.09192970857622330	-0.02921096211069920	0.331506383193897	-3.0385148135928100	0.16698126879376500	97.28970598072350	13.262422234360300	-0.581639006594858	-9.33928358834182
**TPG1_2**	11.52	43.17	0.3158041543732950	0.7799707301558740	-0.17689814287386500	0.522635005762876	0.05705550051366570	-0.24890146516114600	0.42617287414660500	-3.200853148932650	-0.028983829470299500	88.72414890129730	8.436695819854900	-1.126553032745290	-8.119018268541070
**TPG1_3**	11.59	46.95	0.35963410957615800	0.5978279327611020	-0.07460097626838040	0.09911506439888250	-0.01111621270100410	-0.21891357407107200	0.3950212083641190	-3.1066975459685700	0.11939350662997500	96.11513353019090	11.303224120468500	-0.8964202796764180	-8.853349000316490
**TPG1_4**	11.58	44.6	0.3199846662648010	0.5840406280618330	-0.07014304274281470	0.12156710283001800	-0.18917145953591700	-0.19956275735477800	0.36810482335991300	-3.1062449084216300	0.18246233633665500	95.305318695852	9.902313409309110	-0.998360127568026	-8.505827455085830
**TPG1_5**	10.54	41.71	0.3378249953554860	0.39063119622174000	-0.4115288972343880	0.15310783837779700	-0.05969826466591880	-0.12083413510197000	0.5039415455686600	-3.12175147322219	0.15869632724744400	94.66669862440390	9.281143275706130	-1.108287890376240	-8.353980395661020
**TPG1_6**	10.53	41.64	0.3017651864760510	0.7282510768308550	-0.2790485054011800	0.15245824524228000	0.018104236230405000	-0.2425514719719640	0.47973264666583800	-3.1290458575887000	0.1389907546740300	95.124648736779	10.397310854772600	-1.0256266140664500	-8.640090410451310
**TPG1_7**	11.67	41.21	0.7445172469193190	0.369338005493509	0.11954118611566100	-0.28566267574945800	0.10404117535897700	-0.19357936618685300	0.33841120127228200	-2.987429613133420	0.11107514306186500	97.70272983003290	13.890303256663200	-0.6017593399471480	-9.348708981538700
**TPG1_8**	11.12	44.52	0.23643018924975800	1.3462979366101700	-0.480664313767893	-0.07855680494907010	-0.27576879920395300	-0.39851040082656200	0.5756553769397200	-3.017187360255150	0.18408625505678700	94.03667043295210	3.996699310060760	-1.7899643769831000	-6.731588235408690
**TPG1_9**	11.01	42.33	0.2211886433481030	1.0648464632474400	-0.6578181200885150	0.10696930176828300	0.010769042189310500	-0.2742991277406520	0.592452826591267	-3.0657249130374600	0.017901357896793000	90.1940319012553	4.383948563321760	-1.7183120928729600	-6.874763805400930
**TPG1_10**	10.99	43.34	0.34225343996961900	1.0402434556544000	0.06780945803367390	0.5577173774270150	-0.2745000994398770	-0.36632363861501900	0.3590032552308850	-3.263793701219670	0.2436082943554950	93.67021013349910	8.716460528219050	-1.130025117628310	-8.199168707580300
**TPG1_11**	10.13	53.36	0.34856722225431500	0.493720186652315	-0.2314666190671250	-0.6105696309570730	-0.603676651894036	-0.1909940302396600	0.47801840973638100	-2.8743813354375300	0.3884851442785650	97.04211654576020	8.336929773739190	-1.0291225194606300	-7.996865451281240
**IDA3_1**	13.13	76.07	0.23052940654109600	-0.7280918059628950	-1.0733330830544500	-0.4307940549578430	0.36047513696923600	0.3186029573357370	0.6570095747567280	-2.866091200022180	-0.15028761504260200	85.11396742678790	17.013029394787600	0.19583117447100000	-10.164031912430900
**IDA3_2**	13.15	76.54	0.1856773782694450	-0.7980417368583450	-1.1044997765932300	-0.5054696048337910	0.5605040683193500	0.33570721512576800	0.6765194494649010	-2.8557863086061100	-0.1831063815464440	87.988156889599	15.61244545843000	0.18499287820988	-9.877786200449800
**IDA3_3**	13.11	76.23	0.24496181979765900	-0.8448187907879670	-1.1382192600816400	-0.4438443937940410	0.6185438348086880	0.3608762994256920	0.6759897362114990	-2.878148165495920	-0.21453579014239400	85.81128217739030	17.09834392742270	0.1991809903869200	-10.188578851437700
**IDA3_4**	9.742	102.1	0.18879011044024200	1.6223893312560500	-0.08669935492077000	-0.2386798400796220	-0.9243996180159780	-0.5621447275078000	0.4910745870244730	-2.95270839654492	0.44240950084169000	93.5627398651386	3.695064634612020	-1.8006984296599900	-6.60879580903372
**IDA5_1**	12.92	39.72	0.4123440239241630	0.39381746442296400	0.6896135641910850	0.6960780515045610	-0.2826753210707910	-0.20322318182189500	0.046925879083378300	-3.291123902491930	0.2108235299214050	93.79951266705440	33.858938585456600	-0.1770402947599520	-13.009656671504400
**IDA5_2**	10.84	35.24	0.37766125044054800	0.9251180367119370	0.6778487397198640	1.1384323712305600	-0.17558457688958000	-0.3801594738492410	0.09930937252746420	-3.445372227922230	0.17404473272248700	91.71392576609300	38.836253313635400	-0.2101176820910180	-13.767418710696800
**IDA5_3**	11.02	41.24	0.40165873994822300	0.3848296868003730	0.3513778257340470	0.10367068234474300	0.08290072018436190	-0.18521889486561700	0.19612741477473500	-3.086582893324230	0.045749914083516700	94.79265156129410	16.33267351990020	-0.6601953488459220	-9.933078212762220
**IDA5_4**	9.466	44.2	0.4807146850476120	0.6429204165580320	0.4787854909741020	0.5718084921499460	-0.2549640080548540	-0.26759606368225200	0.15764982909912100	-3.259321693018670	0.21961726824251000	94.70542577428620	24.248780118706500	-0.4733386512727840	-11.43986873836240
**IDA5_5**	9.42	33.19	0.4310572283243580	0.6870363439071700	0.6375352071384880	0.6884464008744700	-0.3196539600180710	-0.3029695358066070	0.10757832515266300	-3.289432259550460	0.2368098297301310	93.68308740145380	27.638515733416900	-0.428540113976597	-12.028166427794500
**IDA5_6**	10.48	40.22	0.47362900393207500	0.23407223281145900	0.31990767168405800	0.007658117554973970	-0.1387797647857770	-0.13029981458955900	0.20246785218809300	-3.0420868442487500	0.11367844950468000	93.95952771368590	16.862183480301900	-0.6617716895286240	-10.043264262956700
