# Supplementary material for: Ciliary beating patterns map onto a low-dimensional behavioural space
Source: Nat Phys. Author manuscript; Available in PMC 2025 Jul 10. (PMC12242449; doi:10.1038/s41567-021-01446-2)
Supplement: Supplimentary Info [file NIHMS2088797-supplement-Supplimentary_Info.pdf]

nature portfolio

https://doi.org/10.1038/s41567-021-01446-2

Supplementary information

Ciliary beating patterns map onto a low-dimensional behavioural space

In the format provided by the authors and unedited

SUPPLEMENTARY MATERIAL FOR "CILIARY BEATING PATTERNS MAP ONTO A LOW-DIMENSIONAL BEHAVIORAL SPACE"

Variability of shape parameters, within and between axonemes

We analyzed the variability in frequency, amplitude and wavelength within and between axonemes. To deterine the within variablity (single axoneme level) we devided time series that contained at least 50 beat cycles into parts of 5 beat cycles. These parts were analyzed by Fourier decomposition and the frequency, mean-amplitude and wavelength of the fundamental mode were extracted .

To decide how many parts are nessesary to approximate the variance well we plotted the variance as a function of the number of parts in each time series. In Figure S1, we performed this analysis on 35 wt axonemes (mean and SEM of the variance is shown). We find that using 10 parts (50 beat cycles) the variance reaches saturation ($>80\%$ of the maximum value measured from the longest timesereies of 175 beat cycles).

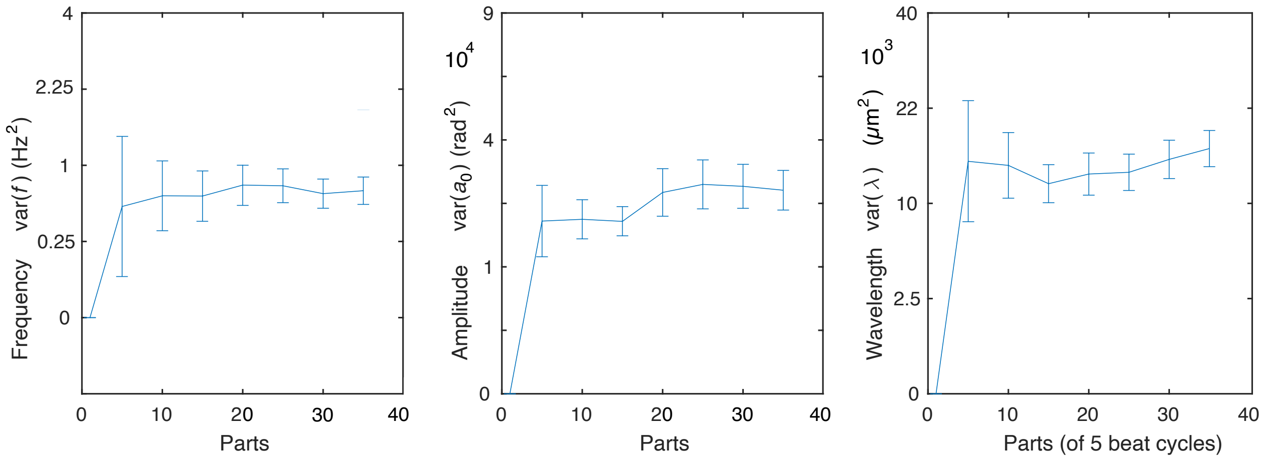

Figure S1. *Variance of shape parameters for single axonemes* Variance of the frequency, mean-amplitude and wavelength and their standard deviations for a population of 35 wt axonemes (1mM ATP, 24°C). On the x-axis the number of analyzed parts (containing 5 beat cycles) is shown.

The variance analysis was performed on axonemes with more than 50 beat cycles. This allowed us to unambiquiously compare the variation in and between axonemes. The data is summarized in Table S1. We find that the within variance, the variation on the single axoneme level (here, mean of the single axoneme variances) is lower than the in between variance, the variation between axonemes (variance of the mean parameters) for all parameters and in all conditions. We quantified this for the different shape parameters by the ratios of the between/ within variance, and report the median values over all condition which are approximately 30 for the frequency, 5 for the amplitude and 30 for the wavelength.

Description of appended data file

The appended file `analysis_data.csv` contains the post-processed data used in this work. The first row contains descriptors of each column, whereas the subsequent 498 rows correspond to the axonemes analyzed in this work. The descriptors are

- `Labels`: a unique label for each axoneme containing information of the experimental conditions (e.g. `ATP_66_4` corresponds to the fourth axoneme under ATP concentration of 66 μM)
- `Length`: the total length L of the axonemes in μm
- `Frequency`: the frequency f in s^{-1}
- `Amplitude`: the mean amplitude a_0
- `PcAmplitude1`: the first principal component of the amplitude a_1^{pc}

- `PcAmplitude2`: the second principal component of the amplitude a_2^{pc}
- `PcPhase1`: the first principal component of the phase φ_1^{pc}
- `PcPhase2`: the second principal component of the phase φ_2^{pc}
- `LegAmplitude1`: the first Legendre coefficient of the amplitude a_1
- `LegAmplitude2`: the second Legendre coefficient of the amplitude a_2
- `LegPhase1`: the first Legendre coefficient of the phase φ_1
- `LegPhase2`: the second Legendre coefficient of the phase φ_2
- `FitScore`: the fitting score R^2
- `k`: the (dimensionless) shearing stiffness k
- `Beta'`: the (dimensionless) instantenous curvature response β'
- `Beta''`: the (dimensionless) instantenous curvature response β''

Pre-processed data, along with scripts to perform the analysis in this work, will be made publicly available on-line upon acceptance of the manuscript.

Strain or Condition	N	Frequency between Var. Var(mean), (Hz²)	Frequency within Var. mean(Var), (Hz²)	Amplitude between Var. Var(mean), (rad²)	Amplitude within Var. mean(Var), (Hz²)	Wavelength between Var. Var(mean), (μm²)	Wavelength within Var. mean(Var), (μm²)
ida5	4	13.58	2.92	0.0035	0.0014	1.50	0.041
mbo2	5	36.01	0.049	0.0028	0.0021	0.26	0.013
***oda1* 24ºC**	5	25.38	0.39	0.0021	0.00067	0.41	0.012
***oda1* 30ºC**	4	68.35	3.05	0.0052	0.00083	0.59	0.048
***oda1* 32ºC**	5	45.90	0.15	0.0020	0.00032	1.35	0.017
***oda1* 34ºC**	3	50.50	0.24	0.0058	0.00043	0.0638	0.0288
***oda1* 38ºC**	5	29.62	2.50	0.0034	0.00051	1.18	0.011
***wt* 24ºC**	18	85.49	1.94	0.0093	0.00066	1.22	0.014
***wt* 26ºC**	12	207.83	4.12	0.0046	0.001	0.88	0.017
***wt* 28ºC**	13	254.40	3.91	0.0069	0.0013	0.99	0.032
***wt* 30ºC**	14	203.58	5.53	0.0078	0.0011	1.38	0.029
***wt* 32ºC**	8	240.86	0.85	0.014	0.00073	0.87	0.0085
***wt* 34ºC**	11	271.28	6.95	0.0088	0.0013	0.97	0.040
***wt* 36ºC**	5	244.65	30.92	0.0046	0.0012	0.59	0.083
***wt* 38ºC**	11	717.91	48.80	0.0050	0.0020	0.58	0.071
***wt* 1000μM ATP**	38	271.13	4.72	0.019	0.00091	0.70	0.027
***wt* 750μM ATP**	30	502.24	18.64	0.016	0.0030	1.93	0.11
***wt* 500μM ATP**	11	318.38	9.8	0.0075	0.0011	1.60	0.033
***wt* 370μM ATP**	14	210.68	8.73	0.0086	0.0033	2.89	0.053
***wt* 240μM ATP**	6	74.32	3.11	0.0087	0.0021	2.001	0.030
***wt* 100μM ATP**	3	11.51	1.89	0.0013	0.0029	0.29	0.036
***wt* 66μM ATP**	7	21.76	1.96	0.012	0.0030	0.79	0.046
***wt* Calcium**	15	112.09	9.25	0.0037	0.0021	3.19	0.088
***wt* Taxol**	16	700.26	391.12	0.0014	0.00061	1.41	3.66
**Median (Mean) Ratio (between/within)**		30 (88)		5 (6)		30 (39)	

Table S1. *Variance analysis of shape parameters* The variances of the frequency, mean-amplitude and wavelength are evaluated for axonemes traces containting 50 beat cycles or more. Shown is the Var(mean), the variance of the population of axonemes within a condition (between variance) and the mean(Var) of single axonemes within one condition (within variance). Mean ratios are the column means, weighted by the sample number.
